# Dermacozine N, the First Natural Linear Pentacyclic Oxazinophenazine with UV–Vis Absorption Maxima in the Near Infrared Region, along with Dermacozines O and P Isolated from the Mariana Trench Sediment Strain *Dermacoccus abyssi* MT 1.1^T^

**DOI:** 10.3390/md19060325

**Published:** 2021-06-03

**Authors:** Bertalan Juhasz, Dawrin Pech-Puch, Jioji N. Tabudravu, Bastien Cautain, Fernando Reyes, Carlos Jiménez, Kwaku Kyeremeh, Marcel Jaspars

**Affiliations:** 1 Marine Biodiscovery Centre, Department of Chemistry, University of Aberdeen, Old Aberdeen AB24 3UE, UK; r01bj16@abdn.ac.uk; 2 Departamento de Biología Marina, Universidad Autónoma de Yucatán, Km. 15.5, Carretera Mérida-Xmatkuil, A.P. 4-116 Itzimná, Mérida 97100, Yucatán, Mexico; dawrin.j.pech@udc.es; 3 School of Natural Sciences, Faculty of Science and Technology, University of Central Lancashire, Preston PR1 2HE, UK; JTabudravu@uclan.ac.uk; 4 Fundación MEDINA, Centro de Excelencia en Investigación de Medicamentos Innovadores en Andalucía, Avda. del Conocimiento 34, Edificio Centro de Desarrollo Farmacéutico y Alimentario, Parque Tecnológico de Ciencias de la Salud, 18016 Granada, Spain; cautainbastien@gmail.com (B.C.); fernando.reyes@medinaandalucia.es (F.R.); 5 Centro de Investigacións Científicas Avanzadas (CICA) e Departmento de Química, Facultade de Ciencias, AE CICA-INIBIC, Universidad da Coruña, 15071 A Coruña, Spain; carlos.jimenez@udc.es; 6 Marine and Plant Research Laboratory of Ghana, Department of Chemistry, School of Physical and Mathematical Sciences, University of Ghana, Legon-Accra P.O. Box LG 56, Ghana; kkyeremeh@ug.edu.gh

**Keywords:** deep sea natural products, Mariana Trench, *Dermacoccus abyssi* MT 1.1^T^, ^13^C-NMR chemical shift linear and multiple regression, (DFT)-UV-Vis spectral calculation, phenoxazine, dermacozine, absorption maxima in the near infrared region

## Abstract

Three dermacozines, dermacozines N–P (**1**–**3**), were isolated from the piezotolerant Actinomycete strain *Dermacoccus abyssi* MT 1.1^T^, which was isolated from a Mariana Trench sediment in 2006. Herein, we report the elucidation of their structures using a combination of 1D/2D NMR, LC-HRESI-MS^n^, UV–Visible, and IR spectroscopy. Further confirmation of the structures was achieved through the analysis of data from density functional theory (DFT)–UV–Visible spectral calculations and statistical analysis such as two tailed *t*-test, linear regression-, and multiple linear regression analysis applied to either solely experimental or to experimental and calculated ^13^C-NMR chemical shift data. Dermacozine N (**1**) bears a novel linear pentacyclic phenoxazine framework that has never been reported as a natural product. Dermacozine O (**2**) is a constitutional isomer of the known dermacozine F while dermacozine P (**3**) is 8-benzoyl-6-carbamoylphenazine-1-carboxylic acid. Dermacozine N (**1**) is unique among phenoxazines due to its near infrared (NIR) absorption maxima, which would make this compound an excellent candidate for research in biosensing chemistry, photodynamic therapy (PDT), opto-electronic applications, and metabolic mapping at the cellular level. Furthermore, dermacozine N (**1**) possesses weak cytotoxic activity against melanoma (A2058) and hepatocellular carcinoma cells (HepG2) with IC_50_ values of 51 and 38 μM, respectively.

## 1. Introduction

**Figure 1:**
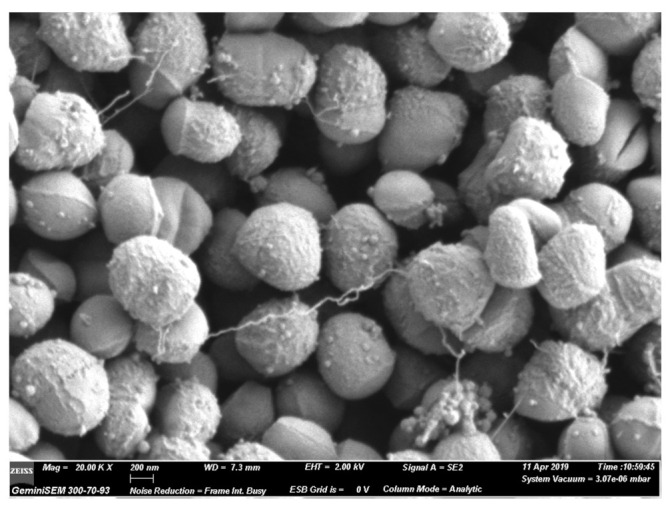
Scanning electron micrograph of the strain *Dermacoccus abyssi* MT 1.1^T^ (Zeiss Gemini SEM 300).

Deep sea habitats have been shown to be an invaluable source of novel bacterial species [1]. Extreme environments (e.g., hyper-arid deserts, bathyal and hadal zones, hot volcanic lakes etc.) are capable of genetically segregating organisms due to their physical properties. Evolutionary adaptations to these extreme environments have generated novel biosynthetic pathways in extremophiles, giving novel structures that may find use in treating diseases [2,3,4]. *Dermacoccus abyssi* MT 1.1^T^ (Figure 1) is a piezotolerant Actinomycete isolated in 2006 from a Mariana Trench sediment, collected at a 10,898 m depth from the Challenger Deep by the remotely operated submersible *Kaiko* in 1998 [5].

**Figure 2:**
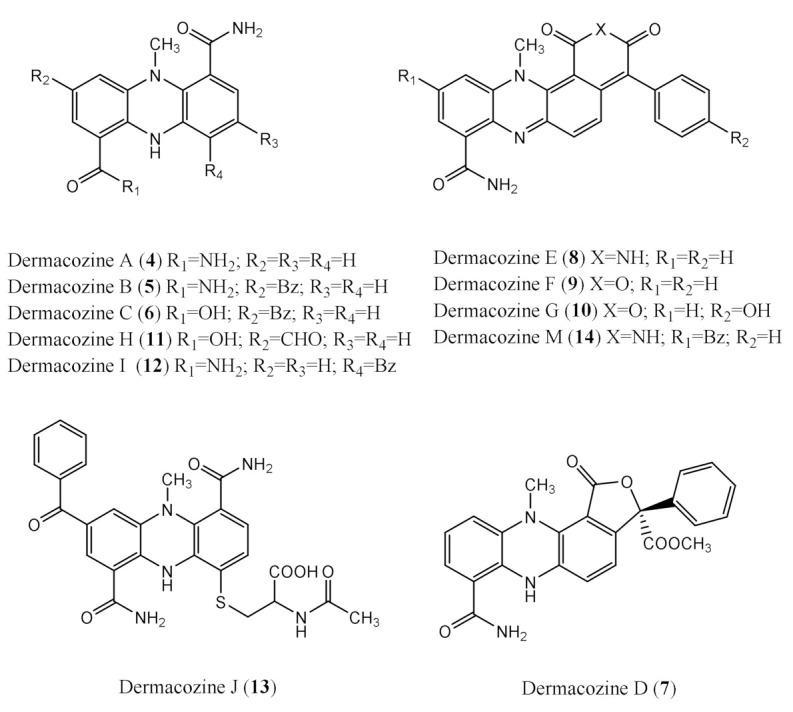
Chemical structures of known dermacozines (**4**–**14**) previously isolated from *Dermacoccus abyssi* MT 1.1^T^ and MT 1.2.

Seven novel phenazines, dermacozines A–G (**4**–**10**), as novel phenazines originating from *Dermacoccus abyssi* strains MT 1.1^T^ and MT 1.2 were reported by our group in 2010. Subsequently, another four new derivatives: dermacozines H–J (**11**–**13**) and dermacozine M (**14**) were isolated and reported with the contribution of our group in 2014 and 2020, respectively (Figure 2) [6,7,8].

These highly pigmented dibenzo annulated pyrazines has kept our interest piqued toward finding further unknown derivatives. Properties of the previously discovered dermacozines include their radical scavenging ability, cytostatic activity against the K562 leukemia cell line, and theoretically calculated non-linear optical behavior [6,7,8,9]. Their IC_50_ cytotoxic activities were reported to be in the range from 7 to 220 μM against the K562 chronic myelogenous leukemia cell line; structure–activity relationship studies revealed a strong connection between the phenazine core linked to a cyclic carboxylic anhydride with their increased activity, but no positive correlation was found in relation to the carboxamide, lactone, or benzoyl moieties [10]. Recently, a synthetic study revealed that the modulation of dermacozine-1-carboxamides, especially with electron releasing substituents (e.g., chloro- and methoxy groups) increases the in vitro anti-tubulin activity of dermacozine-1-carboxamide derivatives comparable to or even superior to the nocodazole control. This evidence gave us reason to further investigate this strain for additional bioactive dermacozine derivatives [11].

## 2. Results and Discussion

*Dermacoccus abyssi* MT 1.1^T^ was phenotypically characterized following the initial isolation from the Mariana Trench sediment; the strain is capable of growing in the presence of 7.5% NaCl, which makes the strain halotolerant [5]. The production of new secondary metabolites has been reported by several authors when salt was added to the culture medium of the organisms capable of living in those conditions [12]. Xie et al. reported the isolation of a new sesquiterpene, ascotrichic acid, when the marine-derived fungus *Ascotricha* sp. ZJ-M-5 was cultivated in 33 g/L ocean salt containing medium [13]. In our recently published article on the full genome sequence of *Dermacoccus abyssi* MT 1.1^T^ and the isolation of dermacozine M (**14**), the strain was cultivated in a GYE seed culture medium initially, then subsequently large-scaled in 35 g/L ocean salt containing ISP2 medium [8]. Altering the preculture conditions has been shown to change the productivity of the strain when dermacozines H–J (**11**–**13**) were isolated [7]. Herein, we report on the isolation of further three dermacozines (**1**–**3**) produced by the strain *Dermacoccus abyssi* MT 1.1^T^ when a seed culture is grown in ISP2 medium containing 20 g/L NaCl, followed by a 35 g/L ocean salt supplemented ISP2 large-scale culture to approximate the deep-sea salinity of 34.7‰ [14].

### 2.1. Structure Determination of Dermacozine N (**1**)

Dermacozine N (**1**) was isolated as a pink amorphous powder. The molecular formula of **1** was established as C_21_H_15_O_3_N_5_ from LC-HRESI-MS^n^ of the [M + H]^+^ ion at *m/z* 386.1247 (calculated *m/z* 386.1248, *∆* = −0.3 ppm) and its ^13^C-NMR spectral data, corresponding to 17 degrees of unsaturation. 

The analysis and the comparison of the molecular formula and calculated DBE with the previous metabolites reported from the same species along with its intense color suggested that **1** must be a dermacozine-like molecule. The presence of the characteristic phenazine substructure (**ABC** rings) in **1**—bearing two carboxamide groups in this particular case—present in most dermacozine structures, was deduced from the NMR data analysis and comparison to those of the reported dermacozines. 

Thus, the ^1^H-NMR spectrum of **1** showed the presence of an N-methyl group at *δ*_H_ 3.68 (3H, s, H-23), which has been a canonical part of the dermacozine structures thus far. The ^1^H-^13^C HMBC correlations from the H-23 methyl hydrogens at *δ*_H_ 3.68 to *δ*_C_ 134.4 (C-4a) and *δ*_C_ 135.5 (C-5a), confirmed the position of the N-methyl group in the pyrazine ring (**B** ring) of the phenazine core. 

**Figure 3:**
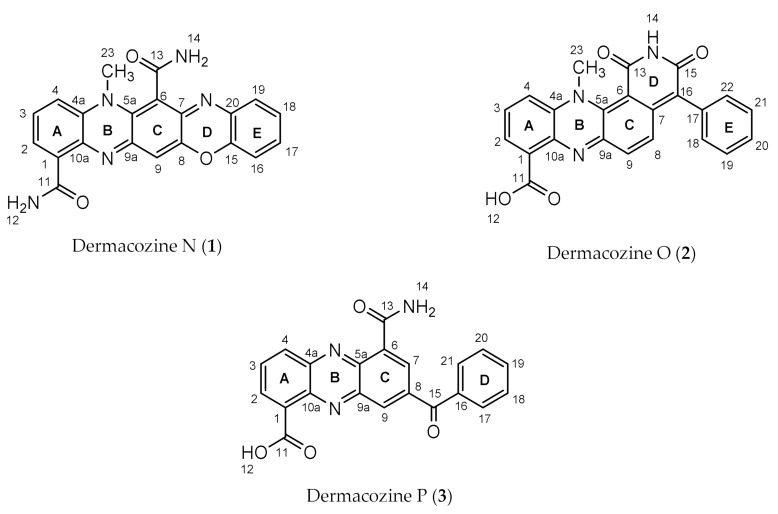
Atom numbering for dermacozine N (**1**), dermacozine O (**2**), and dermacozine P (**3**).

In accordance with other dermacozine structures bearing the phenazine substructure, the ^1^H-^1^H COSY of **1** revealed two characteristic aromatic signals corresponding to the aromatic **A** and **C** rings. The spin system with resonances at *δ*_H_ 7.88 (1H, dd, *J* = 7.6 and 1.3 Hz, H-2), 7.47 (1H, td, *J* = 8.3, and 7.6 Hz, H-3), and 7.55 (1H, dd, *J* = 8.3 and 1.3 Hz, H-4) were indicative of a trisubstituted **A** aromatic ring. The aromatic proton signals of the **A** and **C** rings were correlated to their corresponding carbon resonances at *δ*_C_ 125.9 (C-2), 128.4 (C-3), 118.0 (C-4), and 105.9 (C-9), respectively, through a ^1^H-^13^C HSQC experiment. ^1^H-^13^C HMBC correlations from H-2 at *δ*_H_ 7.88 to *δ*_C_ 166.3 (C-11), *δ*_C_ 118.0 (C-4), and *δ*_C_ 135.1 (C-10a); from H-3 at *δ*_H_ 7.47 to *δ*_C_ 128.9 (C-1) and *δ*_C_ 134.4 (C-4a); from H-4 at *δ*_H_ 7.55 to *δ*_C_ 125.9 (C-2) and *δ*_C_ 135.1 (C-10a) allowed us to fix the positions of the methines at C-2, C-3, and C-4 in relation to the non-protonated carbons at C-1, C-4a, and C-10a confirming the structure of the **A** ring (see ring system in Figure 3).

**Figure 4:**
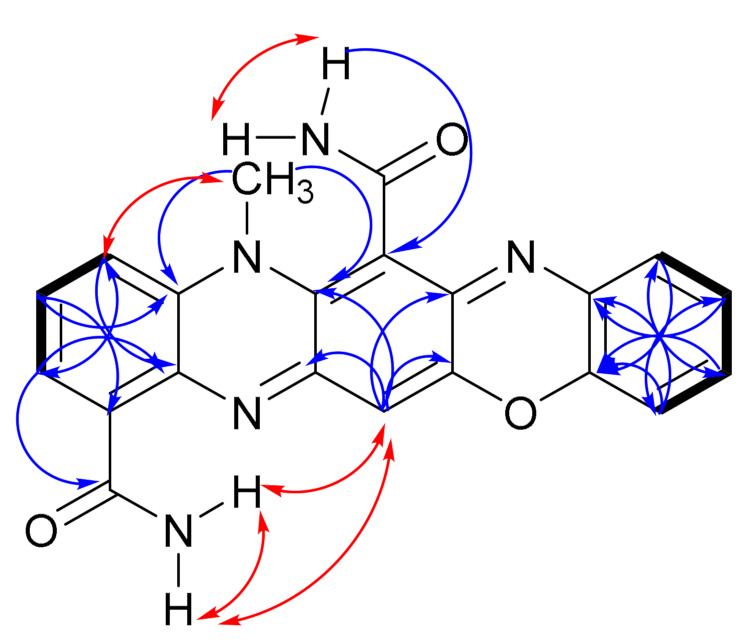
Key 2D NMR COSY (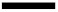), NOESY (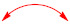) and HMBC (H 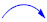 C) correlations of dermacozine N (**1**).

On the other hand, a sharp singlet at *δ*_H_ 6.79 (1H, s, H-9), correlating to its corresponding carbon resonance at *δ*_C_ 105.9 (C-9), by a ^1^H-^13^C HSQC experiment, suggested the presence of a penta-substituted aromatic ring assigned to the **C** ring. The long range ^1^H-^13^C HMBC correlations from H-9 hydrogen at *δ*_H_ 6.79 to *δ*_C_ 151.6 (C-9a), *δ*_C_ 135.5 (C-5a), and *δ*_C_ 149.8 (C-8) were pivotal for the structure elucidation of the **C** aromatic ring as they were consistent with the linear annulation of the phenoxazine structure (Figure 4).

The lack of proton resonances attached to C-7 (present in structures **4**, **5**, **6**, **11**, **12**, **13**) and C-8 (present in structures **4**, **5**, **6**, **7**, **8**, **9**, **10**, **11**, **12**, **13**, **14**) in the ^1^H-NMR of **1** suggested that positions C-7 at *δ*_C_ 148.1 and C-8 at *δ*_C_ 149.8 of the **C** ring must be substituted at these positions.

The presence of the two carboxamide groups in **1**, as in most dermacozine structures, was deduced from a ^1^H-^15^N-NMR HMBC experiment showing two amide nitrogens at *δ*_N_ 112.6 and 118.7 that correlate to their corresponding ^1^H-NMR signals at *δ*_H_ 7.70 (brs, NH-12a)/9.31 (brs, NH-12b) and *δ*_H_ 7.65 (brs, NH-14a)/7.98 (brs, NH-14b). However, only the C-11 amide carbonyl carbon at *δ*_C_ 166.3 could be detected in the ^13^C-NMR spectrum of **1**. The ^1^H-^1^H COSY correlations between the two NH_2_ hydrogens corresponding to each carboxamide group and the characteristic N–H stretch signal at 3439 cm^−1^ detected in the IR spectrum of **1** confirmed the presence of the two carboxamide groups in **1**. The two carboxamide groups were linked to C-6 and C-1 positions in **1** shown by the ^1^H-^13^C HMBC correlations from the NH-14b hydrogen at *δ*_H_ 7.98 (brs) to C-6 at *δ*_C_ 109.8 and from the H-2 hydrogen at *δ*_H_ 7.88 (dd) to the carbonyl of the carboxamide C-11 at *δ*_C_ 166.3, along with the ^1^H-^1^H NOESY correlation from NH_2_-12 to H-9.

Once the phenazine substructure of **1** was confirmed, further NMR analysis allowed us to establish the remaining part of the molecule. 

Eight aromatic carbons were found to be connected to heteroatoms. Four aromatic carbons at δ_C_ 134.4 (C-4a), 135.5 (C-5a), 151.6 (C-9a), and 135.1 (C-10a), linked to nitrogen atoms, were already located in the **B** ring and the two aromatic carbons—at δ_C_ 149.8 (C-8) linked to an oxygen atom, and 148.1 (C-7) linked to a nitrogen atom—were placed in the **C** ring. The locations of the two remaining aromatic carbons connected to heteroatoms with δ_C_ values of 143.3 (C-15) and δ_C_ 134.8 (C-20), attached to oxygen and nitrogen atoms, respectively, were determined as follows.

The ^1^H-NMR spectrum of **1** also displayed the presence of an *ortho*-substituted benzene ring (**E** ring) by the aromatic proton resonances at *δ*_H_ 7.30 (1H, dd, *J* = 7.6 and 1.6 Hz, H-19), 7.15 (1H, ddd, *J* = 7.6, 7.4, and 1.5 Hz, H-18), 7.19 (1H, ddd, *J* = 7.6, 7.4, and 1.6 Hz, H-17), 7.12 (1H, dd, *J* = 7.6 and 1.5 Hz, H-16), which were correlated to their corresponding carbons at *δ*_C_ 127.2 (C-19), 125.3 (C-18), 127.9 (C-17), and, 115.0 (C-16), respectively, demonstrated by the ^1^H-^13^C HSQC spectrum of **1**. The non-protonated carbons at *δ*_C_ 143.3 and *δ*_C_ 134.8, assigned to the C-15 and C-20 carbons, respectively, completed the *ortho*-substituted benzene substructure of the **E** ring. Theoretical chemical shift increment values are consistent with an sp^3^ oxygen atom substitution at C-15, whereas the C-20 chemical shift supports an sp^2^ nitrogen atom substituent [15]. The carbons C-16 and C-18 are *ortho* and *para* position to the nearby oxygen atom, respectively, which possesses two lone pairs of electrons and participate in the positive mesomeric effect, resulting in the shielded carbon (and hydrogen) atoms at these positions. Key ^1^H-^13^C HMBC correlations from *δ*_H_ 7.12 (H-16) to C-15 (*δ*_C_ 143.3), C-20 (*δ*_C_ 134.8), and C-18 (*δ*_C_ 125.3); from *δ*_H_ 7.19 (H-17) to C-15 and C-19 (*δ*_C_ 127.2); from δ_H_ 7.15 (H-18) to C-16 (*δ*_C_ 115.0) and C-20; and from *δ*_H_ 7.30 (H-19) to C-15 and C-17 (*δ*_C_ 127.9) confirmed the *ortho*-substituted benzene ring. 

The 106 unit mass loss observed in the LC-HRESI-MS/MS of **1**, which matched with a loss of a (-ON(C_6_H_4_)-) fragment agrees with the presence of this *ortho*-substituted aromatic **E** ring in **1** (Figures S2 and S3A). The presence of an electron rich π-electron configuration in the vicinity could explain why the C-6 carbon in the structure of **1** at *δ*_C_ 109.8 is shielded in relation to the other dermacozines lacking that ring.

According to the literature in polycyclic aromatic hydrocarbons when five benzene rings are annulated to each other in a linear fashion like in pentacene, the UV-Visible absorption maxima in the visible electromagnetic spectrum compared to the angularly fused benzo[*a*]anthracene shows bathochromic shift [16,17]. The UV–Vis spectra of dermacozine E (**8**), F (**9**), G (**10**), and M (**14**)—exhibiting angular annulation of four rings as seen in benzo[*a*]anthracene—displayed absorption maxima at 576, 566, 580, and 590 nm in the visible electromagnetic spectrum, respectively [6,7,8]. Consequently, the bathochromic shift displayed by the UV–Vis absorption maxima of **1** at 729 and 660 nm supports the annulation of its five aromatic rings. Thus, dermacozine N (**1**) shows a higher extended conjugation than that of dermacozine E (**8**), dermacozine F (**9**), dermacozine G (**10**), and dermacozine M (**14**), which were the dermacozines with the longest visible absorption maxima in their UV–Vis spectra observed to date. Based on this UV–Vis spectral comparison, the core phenazine structure of **1** must be annulated with the (-ON(C_6_H_4_)-) substructure. The correlations observed in the ^1^H-^1^H NOESY spectrum of **1** were crucial confirming this result. The calculated ca. 2.8 Å distance between H-9 and NH_2_-12a and NH_2_-12b hydrogen atoms in the molecular model enabled the linear fusion of the **A**/**B**/**C**/**D**/**E** rings (Theme S1).

In order to support the proposed structure for **1**, we carried out a linear regression analysis and (DFT)–UV–Vis spectral simulation as follows. 

**Figure 5:**
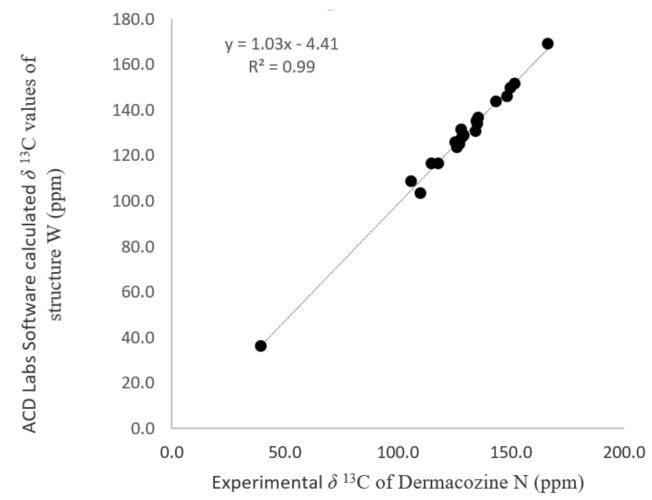
Correlation between experimental ^13^C-NMR chemical shift values (ppm) of dermacozine N (**1**) and those of structure **W** (ACD Labs software (ACD/Structure Elucidator, version 2019.2.0, Neural Network Algorithm, DMSO-*d*_6_).

Twenty-three possible structures of **1** (**A**–**W**) could be drawn in ACD Labs (Figures S34–S38), satisfying the molecular formula of C_21_H_15_O_3_N_5_, the ^1^H-NMR splitting pattern, 2D NMR, ^15^N-NMR, and the presence of the (-ON(C_6_H_4_)-) substructure connected to the core phenazine in an angular or linear fashion. The phenazine biosynthesis occurs through the Shikimic acid pathway and with this as the suggested route of dermacozine biosynthesis, ten structures containing more heteroatoms in the phenazine core than N-5 and N-10 corresponding to **B**, **C**, **F**, **L**, **M**, **O**, **R**, **S**, **T** and **U** were excluded as possible solutions to the current structure. The remaining thirteen possible structures (**A**, **D**, **E**, **G**, **H**, **I**, **J**, **K**, **N**, **P**, **Q**, **V**, **W**) were subjected to statistical analysis. Their ^13^C-NMR chemical shifts were calculated using the ACD Labs software simulation with the Neural Network Algorithm and then the obtained values were subjected to linear regression with the experimental ^13^C-NMR data of **1**. Linear regression between experimental and ACD Labs software predicted ^13^C-NMR chemical shifts has been shown to be an effective method in predicting the correct structures of natural products [18,19,20]. The strongest correlation was observed for structure **W** (12-methyl-12*H*-quinoxalino[2,3-*b*]phenoxazine-8,13-dicarboxamide) with R^2^ = 0.99 (Figure 5), which matched the proposed structure of dermacozine N (**1**), as shown in Figure 4.

**Figure 6:**
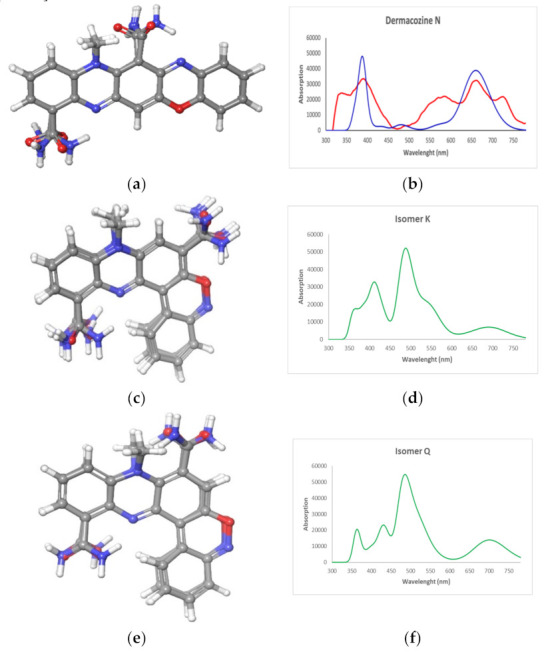
Conformers obtained from the conformational search for (**a**) structure **W** and structures (**c**) **K** and (**e**) **Q**; DFT calculated UV–Vis spectra of structures (**b**) **W** (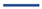) (**d**) **K** and (**f**) **Q** (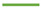) and experimental UV–Vis spectrum (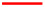) of (**b**) dermacozine N (**1**).

Additionally, we carried out (DFT)–UV–Vis spectral calculations with the proposed structure of **1** and its second and third most likely isomer structures based on the aforementioned linear regression model (structures **K** and **Q**, R^2^ = 0.92 and 0.91, respectively). Time-dependent DFT approach (TDDFT) calculations were used for generating the UV spectra. First, a conformational search of structures **K** and **Q** was performed in the Macromodel module implemented in Maestro Quantum mechanical software (Schrödinger). Using a 4.0 kcal/mol energy threshold from global minimum, ten and eight conformers were found, respectively. The geometry of all these conformers was optimized and their corresponding frequencies were calculated by using a density functional theory (DFT) method at the HSEH1PBE/cc-pVDZ level (see Section 3.4). The resulting UV spectra were combined by Boltzmann weighting to give the composite spectra displayed in Figure 6 [21,22,23].

The TD-DFT calculated UV–Vis absorption maxima of structure **W** at λ_max_ 358 and 659 nm were in excellent agreement with that of the experimental data and these independent calculations support the proposed structure for dermacozine N (**1**). Whereas the absorption maxima at λ_max_ 412 and 488 nm for structure **K** and λ_max_ at 364, 431, and 486 nm for structure **Q** were clearly different from the experimental curve of **1**. Therefore, on the basis of the combined data including 1D/2D NMR, LC-HRESI-MS^n^, MS/MS, UV–Vis spectroscopy data, TD-DFT calculated UV–Vis spectrum, and linear regression made between the calculated and experimental ^13^C-NMR data of **1**, we can conclude that the structure of dermacozine N (**1**) is 12-methyl-12*H*-quinoxalino[2,3-*b*]phenoxazine-8,13-dicarboxamide.

To the best of our knowledge, the framework of dermacozine N (**1**) is unprecedented in natural product chemistry. A synthetic compound bearing the same oxazinophenazine skeleton present in dermacozine N (**1**) was reported by Fischer and Hepp in 1895. The skeleton of this compound, referred to as *“triphenazinoxazine”* in the 19th century was synthesized through nucleophilic substitution of 2-amino-3-phenoxazinone with *ortho*-phenylenediamine [24]. The solvatochromic behavior of the oxazinophenazine and its derivatives in different solvents and the spectacular bathochromic color change upon addition of strong acids and a subsequent hypsochromic shift when this solution was mixed with glacial acetic acid has been described by various authors in the 19th century [24,25,26,27,28]. Fischer and Hepp observed dark red fluorescence of the “*triphenazinoxazine*” in “alcohol solution” and the aforementioned color change from red to violet upon making the solution acidic with mineral acids. In those times, induline derivatives with fluorescent properties were listed under the collective name of “*Fluorindine*”-s [24,25,26,27,28]. In 1978, G.B. Afanas‘eva et al. reported the synthesis of the same oxazinophenazine skeleton when they carried out nucleophilic substitution of 2-ethoxy-3-phenoxazinone with *ortho*-phenylendiamine [29]. Given the compound absorption maxima of **1** in the near infrared spectrum (NIR), its fluorescence spectroscopic investigation would be intriguing in the future.

Fluorophores from nature have gained particular interest among scientists in visualizing physiological processes at the cellular level as the example of one of the most notable discoveries in this field—the isolation of the green fluorescent protein (GFP)—shows [30]. Phenoxazines were found to generate second harmonics (SHG, i.e., frequency doubling) whilst being investigated for non-linear optical properties [31]. Utilizing the opto-electronic features of phenoxazines is widespread in the literature. Benzo[*a*]phenoxazines have been reported to possess useful NIR absorption and emission spectral features as well as solvatochromic effects, thus making them useful in biosensing chemistry (e.g., in pH sensing, glucose sensing, organic biomolecule labelling, and in vivo cellular metabolic mapping [32,33,34,35,36,37,38,39]). Utilizing the long wavelength absorption and emission maxima in visualizing biological processes at the cellular level with fluorogenic probes has gained extreme importance in recent years [37,38,39]. The absorption maxima of the current benzo[*a*]phenoxazine probes is just about or under 700 nm whereas dermacozine N (**1**) at 729 nm possesses the longest absorption maximum in the visible electromagnetic spectrum among the phenoxazines currently being utilized for this purpose [36]. Biosensor molecules exhibiting red or NIR wavelength absorption and emission maxima, as in the case of dermacozine N (**1**), are required for the study of body fluids such as blood, serum, and urine where the matrix may possess components with long wavelength absorption maxima that can interfere with spectroscopic measurements [33].

The cytotoxic activity of dermacozine N (**1**) was investigated against a panel of five human tumor cell lines: human melanoma (A2058) and hepatocellular human carcinoma cell lines (HepG2) exhibiting weak activity, with IC_50_ values of 51 and 38 μM, respectively. Certain phenoxazine derivatives (e.g., Nile Blue analogues were shown to be useful in vitro in photodynamic therapy (PDT) against human bladder carcinoma cells (MGH-U1) acting via the singlet oxygen (^1^O_2_) pathway [40]. In addition to its cytotoxicity, the NIR absorption maxima of dermacozine N (**1**), which would interfere less with the tissue absorption maxima as detailed above, suggests that it may be a suitable compound for further research in the field of photodynamic therapy.

### 2.2. Structure Determination of Dermacozine O (**2**)

Dermacozine O (**2**) was isolated as an ink-blue amorphous powder. The LC-HRESI-MS^n^ measurement gave an *m*/*z* of 398.1125 for [M + H]^+^, consistent with a molecular formula of C_23_H_15_O_4_N_3_ (calculated *m*/*z* 398.1135, *∆* = −2.5 ppm), giving 18 degrees of unsaturation. Interestingly, this molecular formula matches that of previously reported dermacozine F (**9**).

Comparison of the NMR spectra of dermacozine O (**2**) to those of dermacozines E and F (**8** and **9**) showed the same **A**, **B**, **C**, and **E** rings for the three compounds. Indeed, the spin systems identified in the ^1^H-^1^H COSY spectra of **8** and **9** had high similarity to the one in **2**.

The first spin system belonging to the **A** ring (see ring system in Figure 3) was assigned to the H-2, H-3, H-4 hydrogens, which were correlated to their corresponding carbons according to the ^1^H-^13^C HSQC experiment with *δ*_H_/ *δ*_C_ resonances at 7.87 (1H, dd, *J* = 7.5 Hz, 1.1 Hz)/126.6 (C-2); 7.78 (1H, td, *J* = 8.5 Hz, 7.5 Hz)/ 131.3 (C-3); 7.97 (1H, dd, *J* = 8.5 Hz, 1.1 Hz)/120.3 (C-4). The structure of the **A** ring was confirmed by the following ^1^H-^13^C HMBC correlations: from H-2 at *δ*_H_ 7.87 to C-11 at *δ*_C_ 167.2, C-4 at *δ*_C_ 120.3, and C-10a at *δ*_C_ 135.3; from H-3 at *δ*_H_ 7.78 to C-1 at *δ*_C_ 129.6 and C-4a at *δ*_C_ 134.0; and from H-4 at *δ*_H_ 7.97 to C-2 at *δ*_C_ 126.6 and C-10a at *δ*_C_ 135.3. Furthermore, these correlations clearly confirmed the C-1, C-2, C-3, and C-4a, C-10a positions of the **A** ring in relation to the neighboring pyrazine moiety (**B** ring).

A methyl group that resonates as a sharp singlet at *δ*_H_ 3.67 (3H, s, H-23) in the ^1^H-NMR spectrum of **2**, correlated by ^1^H-^13^C HSQC to *δ*_C_ 45.9 (C-23), which was assigned to the characteristic N-methyl attached to the pyrazine ring (**B** ring). The position of this N-methyl group relative to the pyrazine ring was confirmed by the ^1^H-^13^C HMBC correlations from H-23 at *δ*_H_ 3.67 to C-4a at *δ*_C_ 134.0 and C-5a *δ*_C_ 139.5.

The second spin system located in the **C** ring was found to be composed of the H-8 and H-9 hydrogens at *δ*_H_ 7.21 (1H, d, *J* = 9.7 Hz) and 7.24 (1H, d, *J* = 9.7 Hz), which were correlated to *δ*_C_ 134.5 (C-8) and *δ*_C_ 129.7 (C-9), respectively, by the ^1^H-^13^C HSQC experiment. The positions of the H-8/H-9 spin system relative to the C-5a, C-9a, C-6, C-7 in the **C** ring were confirmed by key ^1^H-^13^C HMBC correlations from H-9 at *δ*_H_ 7.24 to C-5a at 139.5 ppm, from H-8 at *δ*_H_ 7.21 to C-9a at *δ*_C_ 150.6, C-6 at *δ*_C_ 100.4, C-7 at *δ*_C_ 139.6, and C-16 at *δ*_C_ 122.6.

**Figure 7:**
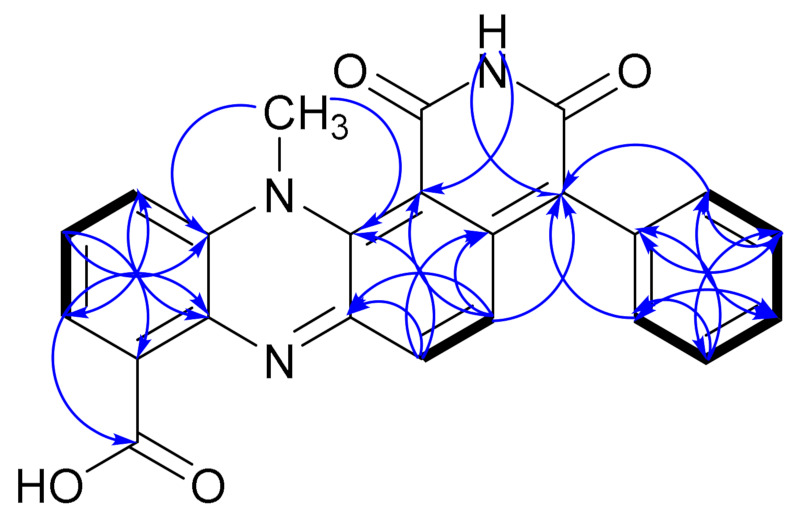
Key 2D NMR COSY (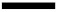) and HMBC (H 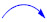 C) correlations of dermacozine O (**2**).

A monosubstituted benzene ring was assigned to the third spin system comprising the five hydrogens corresponding to the **E** ring: H-18/22 at *δ*_H_ 7.30 (2H, dd, *J* = 7.4, 1.3 Hz)/*δ*_C_ 131.3 (C-18/22); H-19/21 at *δ*_H_ 7.47 (2H, td, *J* = 7.4, 1.3 Hz)/ *δ*_C_ 128.2 (C-19/21); and H-20 *δ*_H_ 7.41 (1H, td, *J* = 7.4, 1.3 Hz)/*δ*_C_ 127.9 (C-20). The key ^1^H-^13^C HMBC correlation from H-18/22 at *δ*_H_ 7.30 to C-16 at *δ*_C_ 122.6 ppm allowed us to confirm the connection of the monosubstituted benzene **E** ring to C-16 of the probable **D** ring consisting of a cyclic carboximide (Figure 7).

**Figure 8:**
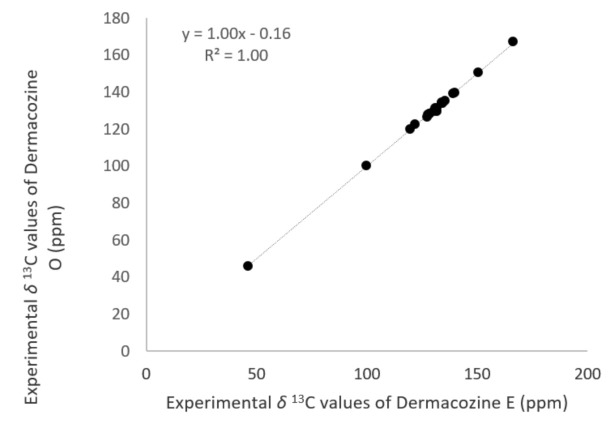
Correlation between the experimental ^13^C-NMR chemical shift values (ppm) of dermacozine O (**2**) and the experimental ^13^C-NMR chemical shift values (ppm) of dermacozine E (**8**).

Since the ^1^H-^1^H COSY spectrum of **8**, **9**, and **10** showed considerable similarity to the ^1^H-^1^H COSY of **2**, statistical comparison was made between the corresponding carbon chemical shifts of these dermacozines. We found that based on the two-tailed *t*-test and multiple regression analysis of the corresponding experimental *δ*_C_ values, the most similar structure to dermacozine O (**2**) is dermacozine E (**8**) (*p* = 2.19 × 10^−8^). Figure 8 shows the linearly correlated experimental ^13^C-NMR resonances of **2** and **8** (see multiple regression in Figure S43).

The most important differences between the NMR spectral data of dermacozine O (**2**) to those of dermacozines E and F (**8** and **9**) were found related to the carbonyl functionalities at position C-11.

The ^1^H-NMR spectrum of **2** showed the presence of a broad singlet at *δ*_H_ 11.27 (1H, brs, NH-14), which matched the cyclic carboximide group present in dermacozine E (**8**). The *δ*_N_ 170.2 resonance observed in the ^1^H-^15^N-NMR of **2** is in agreement with the presence of that functionality and this chemical shift is in the range of ^15^N-NMR chemical shifts of the cyclic carboximide nitrogens present in the structures of dermacozines E (**8**) and M (**14**) [6,8]. Unfortunately, the ^13^C-NMR signals of the C-13 and C-15 carbonyl carbons of **2** were not observed. However, upon comparison of the ^13^C-NMR chemical shifts at position C-6 and C-16, one C–C bond distance from those carbonyl carbons, it was observed that they were closely correlated to the ^13^C-NMR chemical shifts in similar dermacozine structures (**8**–**10** and **14**), providing indirect evidence that C-13 and C-15 must be carbonyl carbons [6,8]. The key ^1^H-^13^C HMBC correlations from H-14 at *δ*_H_ 11.27 of the cyclic carboximide group to C-6 at *δ*_C_ 100.4 and C-16 at *δ*_C_ 122.6 allowed us to confirm the structure of the **D** ring.

On the other hand, even though H-2 at *δ*_H_ 7.87 showed a ^1^H-^13^C HMBC correlation to C-11 at *δ*_C_ 167.2, the lack of signals in the ^1^H-NMR spectrum of **2**, corresponding to the NH_2_ group observed in dermacozines E and F (**8** and **9**), was indicative of the absence of the C-11 carboxamide group in **2**. This information, along with the fact that dermacozine F (**9**) has the same molecular formula as compound **2**, is indicative that the C-11 carboxamide group present in **9** was substituted by a C-11 carboxylic acid in **2**.

Upon assembling the **A**–**E** rings, the structure of dermacozine O (**2**) was determined to be a constitutional isomer of dermacozine F (**9**) and the biosynthetically more closely related C-1 carboxylated derivative of dermacozine E (**8**) with the IUPAC name of 12-methyl-1,3-dioxo-4-phenyl-1,2,3,12-tetrahydropyrido[3,4-*a*]phenazine-8-carboxylic acid. Dermacozine O (**2**) is the first isolated compound bearing a carboxylic acid in its structure among the dermacozine E (**8**), F (**9**), G (**10**), and M (**14**) type of structures.

Due to the absence of some experimental ^13^C-NMR signals, we carried out an ACD Labs simulation using the Neural Network Algorithm, which provided the chemical shift carbon values for the carbonyl resonances of the cyclic carboximide moiety of **2** at *δ*_C_ 163.6 (C-13) and *δ*_C_ 163.1 (C-15), in perfect agreement with the C-13 and C-15 carbon shifts of the aforementioned resembling dermacozine structures.

Dermacozine O (**2**) displayed no cytotoxic activity when tested against A549 (lung carcinoma), A2058 (metastatic melanoma), MCF7 (breast adenocarcinoma), MIA PaCa-2 (pancreatic carcinoma), and HepG2 (hepatocyte carcinoma) cell lines.

The compound as discussed above resembles the structures of **8**, **9**, and **10**, which have been found to exhibit the strongest non-linear optical properties in computational chemistry studies among the previously isolated dermacozines [9].

Following the initial isolation of the dermacozines from the producing *Dermacoccus abyssi* MT 1.1^T^ and MT 1.2, their function in nature has not yet been determined [6,7,8]. Since the survival capability of bacteria that produce the phenazine type of compounds is greater, these compounds are proposed to have defensive functions that protect the producing organism [6,41]. By looking at the structures of dermacozine N (**1**) and dermacozine O (**2**) alongside the previously published dermacozines E (**8**), F (**9**), G (**10**), and M (**14**), the quinonoid **C** rings resemble the quinones playing important roles in electron shuttling in the respiratory process of the cell. Quinone derivatives are found in almost every living organism’s lipid membrane with rare exceptions [42]. Certain phenazine derivatives have been reported to be able to mediate electron transfer from NADPH to molecular O*_2_* [43]. The function of dermacozines in nature is still unknown, but based on this observation, their participation in redox reactions helping the strain to survive (e.g., in microaerobic conditions) is potentially possible. This seems to be supported by the fact that the genome sequence of the bacterium revealed the existence of cytochrome *d* oxidase, which exhibits high affinity to O*_2_* and operates under low oxygen concentrations and multiple copies of succinate dehydrogenases, which can be used as electron donating systems in oxygen deprived conditions [8].

### 2.3. Structure Determination of Dermacozine P (**3**)

Dermacozine P (**3**) was isolated as a purplish amorphous powder. The LC-HRESI-MS^n^ of **3** showed a protonated [M + H]^+^ ion at *m*/*z* 372.0990 [M + H]^+^, consistent with a molecular formula of C_21_H_13_O_4_N_3_ (calculated *m*/*z* 372.0979, *∆*= 3.0 ppm), possessing 17 degrees of unsaturation.

The ^1^H-^1^H COSY spectrum displayed the presence of three spin systems. The first spin system assigned to the **A** ring comprises the three aromatic hydrogens at *δ*_H_ 8.74 (1H, dd, *J* = 7.0 and 1.3 Hz, H-2), 8.21 (1H, td, *J* = 7.0 and 8.6 Hz, H-3), and 8.55 (1H, dd, *J* = 8.6 and 1.3 Hz, H-4). Key long range correlations in the ^1^H-^13^C HMBC spectrum of **3** from H-2 at *δ*_H_ 8.74 to C-10a at *δ*_C_ 141.2; from H-3 at *δ*_H_ 8.21 to C-1 at *δ*_C_ 131.5 and C-4a at *δ*_C_ 142.4; and from H-4 at *δ*_H_ 8.55 to C-10a at *δ*_C_ 141.2 and C-2 at *δ*_C_ 134.9, defined the H-2/H-3/H-4 spin system relative to quaternary C-4a and C-10a carbon atoms, completing the substructure of the **A** ring (see ring system in Figure 3).

**Figure 9:**
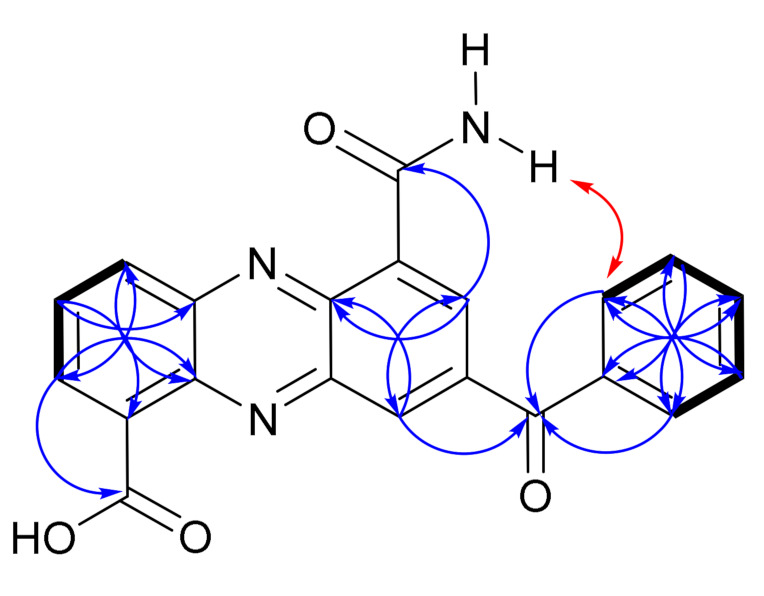
Key 2D NMR COSY (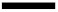), NOESY (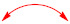) and HMBC (H 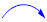 C) correlations of dermacozine P (**3**).

A second spin system corresponded to the aromatic hydrogens H-7 and H-9 with resonances at *δ*_H_ 8.65 (1H, d, *J* = 1.9 Hz) and 8.95 (1H, d, *J* = 1.9 Hz). The ^4^*J*_H7/H9_ coupling constant of 1.9 Hz, indicative of a *meta* relationship between these hydrogen atoms, along with ^1^H-^13^C HMBC correlations from H-7 to C-5a at *δ*_C_ 140.7, C-9 at *δ*_C_ 135.1, C-13 at *δ*_C_ 166.6 and from H-9 to C-15 at *δ*_C_ 194.4, C-7 at *δ*_C_ 131.3, and C-5a at *δ*_C_ 140.7 allowed us to determine the substructure of the **C** ring (Figure 9).

The aromatic hydrogens H-17/21, H-18/20, and H-19 at *δ*_H_ 7.95 (2H, dd, *J* = 7.6 Hz and 1.3 Hz); 7.66 (2H, td, *J* = 7.6 Hz and 1.3 Hz), and 7.78 (1H, td, *J* = 7.6 Hz and 1.3 Hz) of the third spin system were assigned to a monosubstituted benzene corresponding to the **D** ring. Those hydrogens were correlated to their corresponding carbons at *δ*_C_ 130.0 (C-17/21), *δ*_C_ 128.7 (C-18/20), and *δ*_C_ 133.5 (C-19) by a ^1^H-^13^C HSQC experiment of **3.** Finally, C-16—the connection point of the monosubstituted benzene at *δ*_C_ 136.3—was identified by the ^1^H-^13^C HMBC spectrum.

In contrast to all the dermacozine structures (**4**–**14**) reported thus far, the ^1^H-NMR spectrum of **3** showed no evidence of the characteristic N-methyl group attached to the pyrazine ring (**B** ring). Three out of the four carbon atoms of the **B** ring were assigned next to nitrogen: *δ*_C_ 142.4 (C-4a), *δ*c 140.7 (C-5a), and *δ*_C_ 141.2 (C-10a).

When the ^1^H-^13^C HSQC and ^1^H-^13^C HMBC spectra of **3** were overlaid, the presence of three carbonyl carbons at *δ*_C_ 165.5 (C-11), *δ*_C_ 166.6 (C-13), and at *δ*_C_ 194.4 (C-15) was revealed. One of them, C-13, was assigned to a carboxamide substituent from the characteristic primary amide protons that resonate as two broad singlets at *δ*_H_ 8.03 (1H, brs, H-14a) and *δ*_H_ 9.47 (1H, brs, H-14b), both correlated in the ^1^H-^1^H COSY spectrum. The IR band at 3440 cm^−1^ corresponding to a NH stretching confirmed the existence of that substituent. The ^1^H-^13^C HMBC correlation from H-7 at *δ*_H_ 8.65 to C-13 at *δ*_C_ 166.6, along with enhancement of the H-17/21 aromatic protons at *δ*_H_ 7.95 by selective NOE irradiation at the NH-14a at *δ*_H_ 8.03, allowed us to link the carboxamide substituent to position C-6 of the **C** ring.

The **C** ring of the phenazine moiety was connected at C-8 to a monosubstituted benzene **D** ring at C-16 through the C-15 ketone carbonyl group at *δ*_C_ 194.4, confirmed by the ^1^H-^13^C HMBC correlations from the H-9 and H-17/21 protons at *δ*_H_ 8.95 and 7.95, respectively, to C-15. The already mentioned *meta*-coupling (^4^*J* = 1.9 Hz) between H-7 and H-9 of the **C** ring also supports the attachment of C-15 to C-8 (Figure 9).

The ^13^C-NMR chemical shifts of C-6, C-8, and C-9a in **3** were not detected, so these missing values were calculated with ACD Labs software, with the Neural Network Algorithm as *δ*_C_ 129.8, 135.6, and 144.0 ppm, respectively. Upon assembling the structure of **3**, we had one more carbonyl group left with a *δ*_C_ of 165.5 ppm (C-11). Taking into consideration the molecular formula, it was assigned to a carboxylic acid functionality. The connection of this –COOH group was deduced from the ^1^H-^13^C HMBC correlation from H-2 at *δ*_H_ 8.74 to C-11 at *δ*_C_ 165.5. The carboxylic OH-12 was not observed in the ^1^H-NMR spectrum of **3** in DMSO-*d*_6_, therefore we applied a similar approach as in the case of **1** and **2** to confirm the proposed structure for **3**.

**Figure 10:**
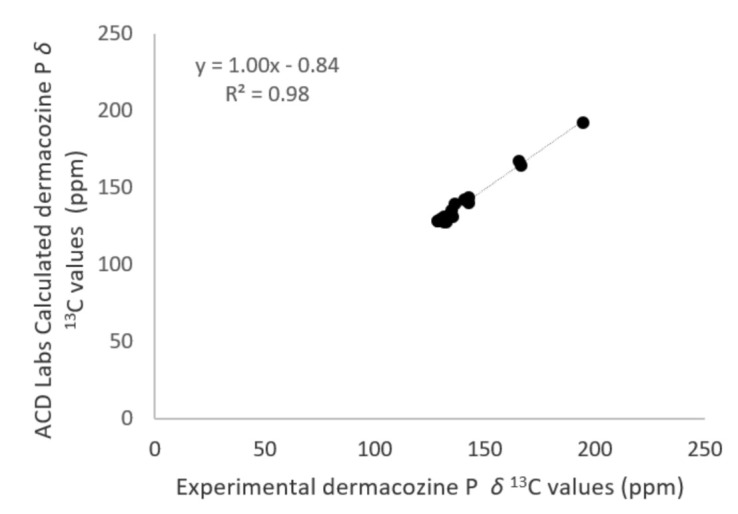
Correlation between the ACD Labs calculated (version 2019.2.0, Neural Network Algorithm, in DMSO-*d*_6_) and experimental ^13^C-NMR chemical shift values (ppm) of dermacozine P (**3**).

We modeled dermacozine P (**3**) with the ACD Labs software (Neural Network Algorithm, DMSO-*d_6_*) and the linear regression curve was obtained between the calculated and experimentally observed ^13^C-NMR chemical shifts. The statistical analysis provided an R^2^ = 0.98 (Figure 10).

**Figure 11:**
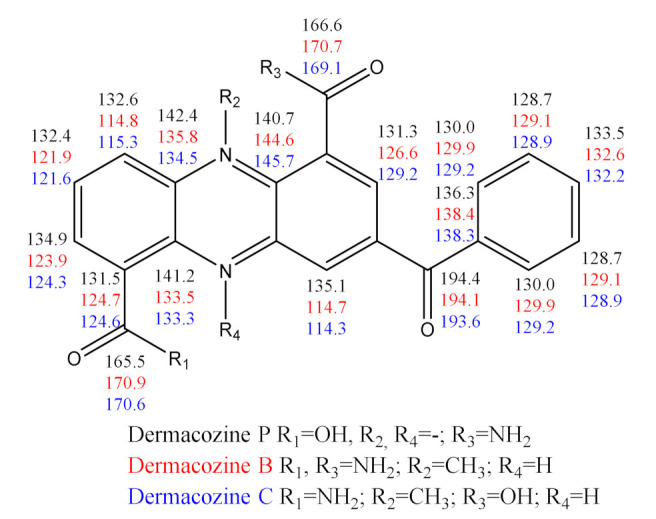
Experimental ^13^C-NMR values (ppm) of dermacozine P (**3**), dermacozine B (**5**), and dermacozine C (**6**).

Upon comparison made between the experimental ^13^C-NMR shifts of **3** versus those chemical shifts observed in the case of **5** and **6,** we could observe a negative mesomeric effect due to the carboxylic group at *ortho*- and *para* positions relative to that functionality in the case of **3** (Figure 11). 

This trend of the ^13^C-NMR chemical shifts provides additional evidence that the substituent at C-1 is more electron withdrawing compared to the one in the case of **5** and **6** and keeping with the carboxylic acid substitution at position C-1. Due to the oxidized phenazine substructure of **3,** the carbon atoms at C-7 and C-9 are more deshielded because of the nitrogen atoms conjugated to them three (N-5) and two bonds (N-10) away, respectively, as opposed to the more shielded aromatic carbons in the case of the more reduced dermacozine B (**5**) and C (**6**) structures (Figure 11). Comparison was made between the UV–Vis spectra of **3**, **5**, and **6** in order to obtain further evidence that the structure of **3** was correct. We found that the electronic structures of **3** and **6** possessed more similarity with the measured *∆*λ_max_ = 5 nm in the visible electromagnetic spectrum, as opposed to the electronic structures of **3** and **5** based on the *∆*λ_max_ = 46 nm measured difference between their visible absorption maxima [6]. This indicates that the 3-benzoyl-phenazine substructure of **3** must be substituted in similar fashion with electron withdrawing functional groups at C-1 and C-6, just like in the structure of **6**. Nevertheless, the hypsochromic shift of **6** of 5 nm compared to the one observed in **3** is in keeping with the more electron deficient phenazine core and the consequently higher degree of conjugation of **3**. To satisfy the observed ^13^C-NMR chemical shifts of **3**, the fit of the linear regression analysis between the experimental and the ACD Labs software calculated (Neural Network Algorithm, DMSO-*d*_6_) ^13^C-NMR shifts of **3** as detailed above, the biosynthetic route considerations (the carboxamide and carboxylic acid functionalities occur at positions C-1 and C-6 in the shikimic acid biosynthetic pathway of dermacozines) as well as the comparison of the UV–Vis absorption maxima in the visible electromagnetic spectrum of **3**, **5**, and **6**, the carboxylic and carboxamide groups are needed to be positioned in the opposite way between the C-1 and C-6 carbon atoms as opposed to the positioning in dermacozine C (**6**). This assignment agrees with the result of the selective NOE experiment, thus placing the –CONH_2_ group at position C-6.

The MS/MS fragmentation data of **3** agreed with our proposed structure, as shown in Figure 9. We were able to identify, among other molecular fragment ions of **3**, the C_20_H_11_N_2_O_2_^+^ fragment (measured *m*/*z* of [M]^+^ as 309) that agrees with the loss of an –OH and a –CONH_2_ fragment keeping with the proposed –COOH group at position C-1. The benzoyl substituted core phenazine fragment molecular ion with a molecular formula of C_19_N_2_O^+^ (measured *m*/*z* of [M]^+^ as 283) following the loss of the –COOH and –CONH_2_ fragments was also apparent in the spectrum (Figure S25).

Therefore, dermacozine P (**3**) is the 5N-demethylated and 6-carboxylated, oxidized analogue of dermacozine B (**5**) and as such, it was chemically identified as 8-benzoyl-6-carbamoylphenazine-1-carboxylic acid.

Dermacozine B (**5**) and C (**6**) showed antioxidant activity in the DPPH assay as well as activity against the K562 leukemia cell line as previously described following their isolation [6]. Given the structural similarity of dermacozine P (**3**) to these substances, further investigation of **3** in anti-tumor and radical scavenging studies would be interesting, especially taking into consideration that the synthetic modulation of the related dermacozine-1-carboxamide derivatives led to increased biological activity in anti-tumor assays as mentioned earlier (see Introduction).

## 3. Materials and Methods

### 3.1. Microorganisms

Pure colonies of *Dermacoccus abyssi* MT 1.1^T^ were provided on an ISP2 agar plate by the School of Biology, University of Newcastle.

### 3.2. Fermentation and Initial Partitioning

A seed culture of *Dermacoccus abyssi* MT 1.1^T^ was prepared as follows: 25 mL of ISP2 medium (yeast extract 4 g, d-glucose 10 g, malt extract 10 g, MilliQ water 1 L, pH 7.0) was supplemented with 20 g/L NaCl. The seed culture incubation was carried out in a 50 mL Falcon tube at 28 °C and 150 rpm for five days. The large-scale fermentation was done in six 2 L Erlenmeyer flasks, each of them containing 1000 mL ISP2 medium (yeast extract 4 g, d-glucose 10 g, malt extract 10 g, MilliQ water 1 L, pH 7.0) supplemented with 35 g/L ocean salt (H2Ocean + Pro Formula with trace elements, The Aquarium Solution Ltd., Hainault Industrial Estate, Ilford Essex, UK). Each flask was inoculated with 1500 μL of the first stage seed culture incubated at 28 °C with agitation at 150 rpm for 14 days in an incubator with a transparent glass cover.

The fermentation broth (6 L) was harvested by the addition of 50 g/L Diaion HP20 resin (>250 μm, Alfa Aesar by Thermo Fisher Scientific, Heysham, Lancashire, U tation for 24 h in a shaker at 28 °C and 150 rpm. The HP20 resin was eluted with methanol (3 × 500 mL) and then with dichloromethane (3 × 500 mL). The successive methanol and dichloromethane extracts were combined and concentrated under reduced pressure yielding the crude material (10,256 mg). The crude material was subjected to liquid–liquid partitioning with the Kupchan method previously used during the isolation of dermacozine M (**14**) [8]. The crude material was suspended in 500 mL H_2_O and extracted three times with an equal volume of dichloromethane in a 1 L separation funnel. The resultant H_2_O layer was then extracted with 3 × 500 mL 2-butanol providing the water fraction (WF; 3498 mg) and the 2-butanol fraction (WB; 2295 mg). The dichloromethane layer was dried under reduced pressure. The dried material was dissolved in 300 mL 9:1 methanol:H_2_O solution and extracted with an equal volume of n-hexane. The n-hexane fraction was dried under reduced pressure resulting in the n-hexane fraction (FH; 65 mg). The remainder 300 mL 9:1 methanol:H_2_O solution after separating it from the n-hexane layer was adjusted to 1:1 methanol:H_2_O solution with 120 mL 100% MilliQ water then extracted with 420 mL dichloromethane three times, which gave the dichloromethane fraction. The dichloromethane layer was separated and dried under reduced pressure (FD; 379 mg). The remainder of the methanol:H_2_O layer was dried providing the methanol fraction (FM; 4019 mg). The dichloromethane fraction (FD; 379 mg) was further separated with silica gel chromatography (Fluorochem, 60A 40-63U MW = 60.083) with a 9:1 dichloromethane:methanol mobile phase as follows: a Quickfit XA42 29/32, 19/26, 19 mm × 500 mm (Fisher Scientific, Loughborough, UK) glass column was used, packed with 200 g silica (~550× of the mass of the FD fraction), with a flow rate of ~0.8 mL/min, collected into 10 mL vials. Bands were collected based on their visible colors but they also showed fluorescence under UV light (360 nm). In the silica chromatography of the FD fraction, 35 fractions were obtained (S1–S35). These fractions were combined based on TLC (thin layer chromatography) experiments and purified further, as detailed in the Section 3.5 (please see the TLC plate of the FD fraction with dichloromethane:methanol 9:1 mobile phase and the colorful bands in Theme S2).

### 3.3. Instrumentation

High performance liquid chromatography: Semi-preparative Gradient Agilent HPLC apparatus (1100 series, Agilent—Santa Clara, Cequipped with binary pump, diode array detector (G1315B, Agilent—Santa Clara, CSunfire C_18_ reversed phase column (5 μm, 10 × 250 mm), and ACE HL C_18_ reversed phase column (5 μm, 10 × 250 mm).

Nuclear magnetic resonance spectrometers: For the structure determination of **1** and **2**, a Bruker 800 MHz NMR spectrometer (Bruker Biospin—Billerica, Merating with a 5 mm TCI He Cryoprobe was used. 1D ^1^H-NMR and magnitude 2 D NMR experiments were run apart from the ^1^H-^13^C HMBC of **1**, where an additional phase sensitive experiment was conducted. In the case of compound **3**, 1 D ^1^H-NMR and magnitude mode 2 D NMR experiments were run including a selective NOESY experiment with a Bruker 600 MHz NMR spectrometer (Bruker Biospin—Billerica, MCE III HD operating with a N_2_ Cryoprobe. HSQC experiments were obtained using 2D H-1/X correlation via the double inept transfer phase sensitive using Echo/Antiecho-TPPI gradient selection; with decoupling during acquisition; using trim pulses in inept transfer; with multiplicity editing during selection step, shaped pulses for inversion on f2—channel for matched sweep adiabatic pulses; for HMBC, experiments were obtained with 2D H-1/X correlation via heteronuclear zero and double quantum coherence, phase sensitive and magnitude mode in the case of **1**, and magnitude mode for **2** and **3**, using Echo/Antiecho gradient selection with two-fold low-pass *J*-filter to suppress one-bond correlations with no decoupling during acquisition [44,45,46,47,48,49].

Liquid chromatography-mass spectrometry: **1** and **2** were analyzed with a Bruker MAXIS II qTOF LC-MS instrument. LC-qTOF utilizes a Phenomenex Kinetex XB-C18 column (2.6 μm; 100 × 2.1 mm) with a mobile phase of 5% acetonitrile + 0.1% formic acid to 100% acetonitrile + 0.1% formic acid with a run time of 11 min. **3** was analyzed with a Thermo Instruments MS system (LTQ-XL Orbitrap Discovery) coupled to a 1290 Infinity Agilent UHPLC system, utilizing a Phenomenex Kinetex XB-C18 column (2.6 μm; 100 × 2.1 mm) with a mobile phase of 5% acetonitrile + 0.1% formic acid to 100% acetonitrile + 0.1% formic acid in 25 min. The analytes were diluted to 0.01 mg/mL concentration with methanol.

Infrared spectra were recorded in ethanol with a Perkin Elmer Spectrum Two FTIR spectrometer Perkin Elmer, MVis Spectroscopic data were recorded in ethanol using a Thermo Scientific Evolution 201 UV–Visible Spectrophotometer Thermo, M. Computational Calculations

A Macromodel module implemented in the Maestro Quantum mechanical software (Maestro Schrödinger LLC, New York, N was used for conformational searches [21]. The calculations were carried out by using an OPLS 2005 force field with ethanol as the solvent. A torsional enhanced sampling with 1000 or 10,000 steps was fixed using an energy window of 4.0 kcal/mol. Molecular geometry optimizations were performed at the DFT theoretical level using the Gaussian 09W package with a HSEH1PBE/cc-pVDZ auto for energy and frequency calculations. After removing redundant conformers and those with imaginary frequencies, theoretical Boltzmann energy population-weighted UV–Vis spectra were calculated by using PBEPBE/6-311++g(3d,2p) with 24 states [22]. The open software SpecDis V.1.71 (Berlin, Germany, 2017, https:/specdis-software.jimdo.com, (accessed on the 7th y 2020 and on the 14 April 2021)) was used to obtain the graphical theoretical UV–Vis curves [23].

### 3.4. Isolation of Compounds

Dermacozine N (**1**) was obtained with further purification of the initial silica fractions detailed in Section 3.2 as follows: the first fraction (S1) of the initial FD fractionation with silica chromatography gave dermacozine M (**14**) as reported by Abdel-Mageed et al. [8]. The S2–S3 deep purple colored fractions of the initial silica chromatography as described in Section 3.2 were combined based on TLC chromatography, dried in a N_2_ drier (57.3 mg), and further partitioned with silica glass column chromatography. A Quickfit CR 20/30 19/26 glass column was used, packed with ~20 g silica (~350× of the mass of the initial mass of the combined S2–S3 silica chromatography FD fraction), and the mobile phase was 9:1 dichloromethane:methanol also in this case. In this experiment, fifteen (SA1–SA15) fractions were collected, each into 7 mL vials with a flow rate of ~0.4 mL/min. The SA9–SA11 pink colored fractions of this experiment were combined based on TLC. Keeping this fraction in a cold room at 4 °C, a pink precipitate appeared, which was centrifuged at 10,000 rpm for 15 min. Upon removal of the supernatant and drying the substance in N_2_ drier, the substance appeared to be a pure compound (1.6 mg) according to the ^1^H-NMR and the LC-HRESI-MS^n^ measurements (Figures S1 and S6).

Dermacozine O (**2**) was obtained with further purification of the initial silica fractions detailed in Section 3.2 as follows: the S5–S8 red colored silica fractions (28.2 mg) were combined based on TLC chromatography, dried in a N_2_ drier, and subjected to HPLC purification. High performance liquid chromatography purification of dermacozine O (2) was carried out isocratically with a methanol/H_2_O 60%/40% + 0.1% trifluoroacetic acid solvent system over 40 min with 1.8 mL/min flowrate, and with Sunfire C_18_ reversed phase column (5 µm; 110 × 250 mm). Peak detection and UV–Vis trace analysis was carried out at the wavelengths of 254 ± 5 nm, 280 ± 5 nm, 330 ± 5 nm, 350 ± 5 nm, and 530 ± 25 nm. Material collection was automated based on UV–Vis peak appearance versus time. The HPLC fraction with RT = 31.9 min yielded (3.1 mg) the pure compound (see Figures S13, S16 and Theme S3).

Dermacozine P (**3**) was obtained with further purification of the initial silica fractions detailed in Section 3.2 as follows: the initial S4 (7 mg) blue colored silica fraction of the dichloromethane Kupchan fraction (FD) was dried in N_2_ drier and subsequently repurified with High performance liquid chromatography. The HPLC purification of dermacozine P (**3**) was carried out with a gradient from the initial solvent ratio of 0% methanol and 95% H_2_O + 5% methanol + 0.05% trifluoro-acetic acid to 100% methanol and 0% H_2_O over 40 min with 2 mL/min flowrate, and with an ACE C_18_ HL reversed phase column (5 µm; 110 × 250 mm). Peak detection and the UV–Vis trace analysis was carried out at the wavelengths of 250 ± 5 nm, 280 ± 5 nm, 500 ± 25 nm, 550 ± 25 nm, and 600 ± 25 nm. Material collection was automated based on UV–Vis peak appearance versus time. The HPLC fraction with RT = 15.9 min yielded (3.4 mg) the pure compound (see Figures S24, S26 and Theme S4).

### 3.5. Cytotoxic Activity of Dermacozines against Human Tumor Cell Lines

The cytotoxic activity of dermacozines N (**1**) and O (**2**) against five different human cancer cell lines, namely A549 (lung carcinoma), A2058 (metastatic melanoma), MCF7 

(breast adenocarcinoma), MIA PaCa-2 (pancreatic carcinoma), and HepG2 (hepatocyte carcinoma) (obtained from ATCC, Manasas, V [50] was studied based on the MTT (3-(4,5-dimethylthiazol-2-yl)-2,5-diphenyltetrazolium bromide) assay [51]. The compounds were tested as a 10-point dose-response curve (½ serial dilutions) starting at a concentration of 20 µg/mL in triplicate. IC_50_ values were determined as previously described [50]. Dermacozine P (**3**) was not tested against cancer cell lines.

### 3.6. Chemical Characterization of Compounds

Dermacozine N (**1**): pink amorphous powder, 1.6 mg; LC-HRESI-MS^n^ (qTOF, Bruker), *m/z* measured 386.1247 [M + H]^+^, 408.1068 [M + Na]+, 772.2448 [2M + H]^+^, 793.2225 [2M + Na]^+^, *∆* = −0.3 ppm for calculated *m/z* of [M + H]^+^ 386.1248; DBE = 17; MF = C_21_H_15_O_3_N_5_. UV λ_max_^EtOH^ = 729, 660, 577, 364, 309 nm. IR^EtOH^ = 3439, 2988, 1662, 1066, 1039, 544, 521, 471, 463 cm^−1^. ^1^H- and ^13^C-NMR data (DMSO-*d*_6_), see Table 1.

Dermacozine O (**2**): ink blue amorphous powder, 3.1 mg; LC-HRESI-MS^n^ (qTOF, Bruker) *m/z* 398.1125 [M + H]+, *∆* = −2.5 ppm for calculated *m/z* of [M + H]^+^ 398.1135, DBE = 18; MF = C_23_H_15_O_4_N_3_. UV λ_max_^EtOH^ = 644, 563, 463, 396(sh), 308 nm. IR^EtOH^ = 2971, 1989, 1949, 1091, 1048, 882, 524, 501, 481, 453 cm^−1^. ^1^H- and ^13^C-NMR data (DMSO-*d*_6_), see Table 1.

Dermacozine P (**3**): purplish amorphous powder, 3.4 mg; LC-HRESI-MS^n^ (Orbitrap, Xcalibur) *m/z* 372.0990 [M + H]^+^, *∆* = 3.0 ppm for calculated *m/z* of [M + H]^+^ 372.0979; DBE = 17; MF = C_21_H_13_O_4_N_3_. UV λ_max_^EtOH^ = 465, 415(sh), 363 nm. IR^EtOH^ = 3440, 3178, 1661, 872, 757, 603, 575, 556, 534, 523, 465, 437, 410 cm^−1^. ^1^H- and ^13^C-NMR data (DMSO-*d*_6_), see Table 1.

### 3.7. Regression Models Used in the Structure Elucidation

We used a linear regression model in the case of dermacozine N (**1**). The experimental* ^13^C-NMR chemical shifts of **1** were correlated to the ACD Labs software calculated ones (ACD/Structure Elucidator, version 2019.2.0, Neural Network Algorithm, solvent used for simulation: DMSO-*d*_6_) in their theoretically possible structures (**A**–**W**). The simple linear regression model showed clear difference between the possible structures keeping with the results of other modalities in the structure elucidation process. The correlation coefficient obtained was R^2^ = 0.99 (See Section 2.1).

In the case of dermacozine O (**2**), we compared its experimental ^13^C-NMR chemical shift values to the ones reported in structures **8**, **9**, and **10** [6] due to their structural similarity. The experimentally not observed ^13^C-NMR shifts (e.g., C-13 and C-15 in **2**) were excluded from the regression analysis, and since in dermacozine M (**14**) C-3 was calculated with ACD Labs software and contains an additional benzoyl ring at C-3, we excluded this structure from the calculations. A multiple regression test was carried out that showed significant similarity between dermacozine O (**2**) and dermacozine E (**8**) based on the *p-*value and *t*-test (see Section 2.2).

In the case of dermacozines P (**3**), linear regression was done between the ACD Labs software calculated (ACD/Structure Elucidator, version 2019.2.0, Neural Network Algorithm, solvent used for simulation: DMSO-*d*_6_) and its experimentally observed ^13^C-NMR chemical shift values. The R^2^ value obtained was R^2^ = 0.98 (See Section 2.3).

*The experimental ^13^C-NMR values for the statistical calculations were measured in DMSO-d_6_ in the case of structures **1**, **2**, and **3**.

## 4. Conclusions

*Dermacoccus abyssi* MT 1.1^T^ is a piezotolerant, halotolerant Actinomycete from the deepest part of the Earth, the Mariana Trench, Challenger Deep. Since the discovery of the strain in 2006, more than a dozen novel, highly colored dermacozines have been isolated from it. This work provides evidence that the bacterium ability of producing new compounds is still not exhausted yet.

Herein, we report on the isolation of three new dermacozines; dermacozine N (**1**), dermacozine O (**2**), and dermacozine P (**3**) when the strain was seed cultured in 20 g/L containing NaCl medium, followed by the cultivation in ISP2 medium, approximating the salinity of its usual habitat of 34.7‰. The difficulty of structure elucidation arising from the polycyclic nature of dermacozines was overcome by the combined approach of utilizing linear/multiple linear regression of experimental and ACD Labs software calculated ^13^C-NMR chemical shifts, (TD-DFT) UV–Vis spectral simulation, 1D/2D NMR, LC-HRESI-MS^n^, biosynthetic route considerations, which step by step offered complementary evidence for our structural assignments.

In dermacozine N (**1**)—whose phenoxazine skeleton is unprecedented in nature to the best of our knowledge—the [*b*]benzopyrazine substitution of the core phenoxazine resulted in UV–Vis absorption maxima in the near infrared (NIR) region. Phenoxazines have been reported to exhibit non-linear optical properties; nevertheless the structure of dermacozine O (**2**) is highly similar to those of dermacozines E (**8**), F (**9**), G (**10**), which were suggested to also exhibit non-linear optic properties in computational chemistry studies. This gives a perspective of investigating **1** and **2** in opto-electronics and second harmonic generation. Given the NIR absorption spectrum of **1**, the reported solvatochromic behavior, and bathochromic shift observed in acidic conditions of related phenoxazine skeletons, **1** would be an outstanding candidate to conduct research on in biosensing chemistry, biomolecular labelling, and *in vivo* metabolic mapping at the cellular level. Compound **1** showed weak activity against melanoma (A2058) and hepatocellular human carcinoma cell lines (HepG2) with IC_50_ values of 51 and 38 μM, respectively. Dermacozine P (**3**) is the oxidized, 5N-demethylated, and C-6 carboxylated derivative of dermacozine B (**5**). Based on the literature data and cytostatic activities of the previously reported dermacozine derivatives and that synthetic modulation of dermacozine-1-carboxamides increased the compounds’ anti-tubulin activity, further anti-tumor studies involving synthetic modulation of the compounds of dermacozine N (**1**), O (**2**), and P (**3**) would be promising.

Fascinating research is being conducted in various fields of chemistry to be able to utilize the novelty of these vividly colored compounds including opto-electronics, computational chemistry, biosensing chemistry, and medicinal chemistry exhibiting exciting results. Whilst the potential use of the colorful dermacozines in chemistry keeps stimulating the interest of researchers, their biological role in the mesmerizing depth of the hadal zone remains unknown.

## Figures and Tables

**Table 1:** NMR spectroscopic data for dermacozine N (**1**) (800 MHz, DMSO-*d*_6_), dermacozine O (**2**) (800 MHz, DMSO-*d*_6_), and dermacozine P (**3**) (600 MHz, *DMSO*-*d*_6_).^†^: Calculated with ACD Labs software, Nural Nework Algrithm, DMSO-*d*_6_ (Chemical shift is not observed).

*N.*	*Dermacozine N (1)*		*Dermacozine O (2)*		*Dermacozine P (3)*	
	*δ*_C_, mult	*δ*_H_, mult (*J* in Hz)	*δ*_C_, mult	*δ*_H_, mult (*J* in Hz)	*δ*_C_, mult	*δ*_H_, mult (*J* in Hz)
1	128.9, C		129.6, C		131.5, C	
2	125.9, CH	7.88 (dd, 7.6, 1.3)	126.6, CH	7.87 (dd, 7.5, 1.1)	134.9, CH	8.74 (dd, 7.0, 1.3)
3	128.4, CH	7.47 (td, 8.3, 7.6)	131.3, CH	7.78 (td, 8.5, 7.5)	132.4, CH	8.21 (td, 7.0, 8.6)
4	118.0, CH	7.55 (dd, 8.3, 1.3)	120.3, CH	7.97 (dd, 8.5, 1.1)	132.6, CH	8.55 (dd, 8.6, 1.3)
4a	134.4, C		134.0, C		142.4, C	
5a	135.5, C		139.5, C		140.7, C	
6	109.8, C		100.4, C		129.8, C ^†^	
7	148.1, C		139.6, C		131.3, CH	8.65 (d, 1.9)
8	149.8, C		134.5, CH	7.21 (d, 9.7)	135.6, C ^†^	
9	105.9, CH	6.79, s	129.7, CH	7.24 (d, 9.7)	135.1, CH	8.95 (d, 1.9)
9a	151.6, C		150.6, C		144.0, C ^†^	
10a	135.1, C		135.3, C		141.2, C	
11	166.3, C		167.2, C		165.5, C	
12		A 7.70, brs		COOH, not observed		COOH, not observed
		B 9.31, brs				
13	168.3, C ^†^		163.6, C ^†^		166.6, C	
14		A 7.65, brs				A 8.03, brs
		B 7.98, brs		11.27, brs		B 9.47, brs
15	143.3, C		163.1, C ^†^		194.4, C	
16	115.0, CH	7.12 (dd, 7.6, 1.5)	122.6, C		136.3, C	
17	127.9, CH	7.19 (ddd, 7.6, 7.4, 1.6)	134.1, C		130.0, CH	7.95 (dd, 7.6, 1.3);
18	125.3, CH	7.15 (ddd, 7.6, 7.4, 1.5)	131.3, CH	7.30 (dd, 7.4, 1.3)	128.7, CH	7.66 (td, 7.6, 1.3)
19	127.2, CH	7.30 (dd, 7.6, 1.6)	128.2, CH	7.47 (td, 7.4, 1.3)	133.5, CH	7.78 (td, 7.6, 1.3)
20	134.8, C		127.9, CH	7.41 (td, 7.4, 1.3)	128.7, CH	7.66 (td, 7.6, 1.3)
21			128.2, CH	7.47 (td, 7.4, 1.3)	130.0, CH	7.95 (dd, 7.6, 1.3);
22			131.3, CH	7.30 (dd, 7.4, 1.3)		
23	39.5, CH_3_	3.68, s	45.9, CH_3_	3.67, s	-	-

## Data Availability

Not applicable.

## Supplementary Materials

The following are available online at https://www.mdpi.com/article/10.3390/md19060325/s1, Figure S1: LC-MS analysis of dermacozine N (**1**) (qTOF, Bruker), Figure S2: MS/MS of dermacozine N (**1**) (qTOF, Bruker), Figure S3A: Proposed MS fragmentation pathway of dermacozine N (**1**) showing loss of a (-ON(C_6_H_4_)-) fragment, Figure S3B: Mass error between the calculated (Chemdraw modeled) and experimental (qTOF) *m/z* ratio of dermacozine N (**1**), Figure S4: UV–Vis spectrum of dermacozine N (**1**) in EtOH, Figure S6: Dermacozine N (**1**) ^1^H-NMR spectrum (800 MHz, DMSO-*d*_6_), Figure S7: ^1^H-^13^C HSQC spectrum of dermacozine N (**1**) (800 MHz, DMSO-*d*_6_), Figure S8: ^1^H-^13^C HMBC (magnitude mode) spectrum of dermacozine N (**1**) (800 MHz, DMSO-*d*_6_), Figure S9: ^1^H-^13^C HMBC spectrum of dermacozine N (**1**) with *J* = 2 Hz (800 MHz, DMSO-*d*_6_), Figure S10: ^1^H-^1^H COSY spectrum of dermacozine N (**1**) (800 MHz, DMSO-*d*_6_), Figure S11: ^1^H-^1^H NOESY spectrum of dermacozine N (**1**) (800 MHz, DMSO-*d*_6_), Figure S12: ^1^H-^15^N HMBC spectrum of dermacozine N (**1**) (800 MHz, DMSO-*d*_6_), Figure S13: LC-MS analysis of dermacozine O (**2**) (qTOF, Bruker), Figure S14: UV-Vis spectrum of dermacozine O (**2**) in EtOH, Figure S15: IR spectrum of dermacozine O (**2**) in EtOH, Figure S16: ^1^H-NMR spectrum of dermacozine O (**2**) (800 MHz, DMSO-*d*_6_), Figure S17: ^1^H-^13^C HSQC spectrum of dermacozine O (**2**) (800 MHz, DMSO-*d*_6_), Figure S18: ^1^H-^13^C HMBC spectrum of dermacozine O (**2**) (800 MHz, DMSO-*d*_6_), Figure S19: Long Range ^1^H-^13^C HMBC correlations of dermacozine O (**2**) with *J* = 2 Hz (800 MHz, DMSO-*d*_6_), Figure S20: ^1^H-^1^H COSY spectrum of dermacozine O (**2**) (800 MHz, DMSO-*d*_6_), Figure S21: ^1^H-^15^N HMBC spectrum of dermacozine O (**2**) (800 MHz, DMSO-*d*_6_), Figure S22: UV-Vis spectrum of dermacozine P (**3**) in EtOH, Figure S23: IR spectrum of dermacozine P (**3**) in EtOH, Figure S24: LC-MS analysis of dermacozine P (**3**) (Orbitrap, Xcalibur), Figure S25: MS/MS analysis of dermacozine P (**3**) (Orbitrap, Xcalibur), Figure S26: ^1^H-NMR spectrum of dermacozine P (**3**) (600 MHz, DMSO-*d*_6_), Figure S27: ^1^H-^13^C HSQC spectrum of dermacozine P (**3**) (600 MHz, DMSO-*d*_6_), Figure S28: ^1^H-^13^C HMBC spectrum of dermacozine P (**3**) (600 MHz, DMSO-*d*_6_), Figure S29: ^1^H-^1^H COSY spectrum of dermacozine P (**3**) (600 MHz, DMSO-*d*_6_), Figure S30: 1D-NOESY spectrum of dermacozine P (**3**) (600 MHz, DMSO-*d*_6_) from irradiation of the signal at 8.03 ppm, Figures S31-S33: ^13^C-NMR *δ* calculated values of dermacozine A–P (**1**–**14**) using the ACD Labs software with Neural Network Algorithm, solvent DMSO-*d*_6_, Figures S34-S38: ^13^C-NMR *δ* calculated values of possible structures (**A**–**W**) of dermacozine N (**1**), using the ACD Labs software with Neural Network Algorithm, solvent DMSO-*d*_6_, Table S1: Comparison between calculated vs. experimental ^13^C-NMR *δ* values of dermacozines A–J (**4**–**13**), Figure S39: Linear regression graphics between ACD Labs (Neural Network Algorithm, DMSO-*d*_6_) calculated vs. experimental ^13^C-NMR *δ* values of dermacozine A-H (**4**–**11**), Figure S40: Linear regression graphics between ACD Labs (Neural Network Algorithm, DMSO-*d*_6_) calculated vs. experimental ^13^C-NMR *δ* values of dermacozine I & J (**12**–**13**), Table S2: Comparison between the experimental ^13^C-NMR *δ* values of dermacozine N (**1**) vs. the calculated ones of possible structures **A**, **D**, **E**, **G**, **H**, **I**, **J**, **K**, **N**, **P**, **Q**, **V**, **W** by ACD Labs (Neural Network Algorithm, DMSO-*d*_6_), Figure S41: Linear regression graphics between ^13^C-NMR *δ* experimental values of dermacozine N (**1**) vs. the ACD Labs calculated ones (Neural Network Algorithm, DMSO-*d*_6_) (**4**–**13**), Figure S42: Linear regression graphics between ^13^C-NMR *δ* experimental values of dermacozine N (**1**) vs. the ACD Labs (Neural Network Algorithm, DMSO-*d*_6_) calculated ones for possible structures of **N**, **P**, **Q**, **V**, **W**, Table S3: Absolute error and the standard deviation of the error from the mean between the calculated (Structure **W**) and the experimental dermacozine N (**1**) ^13^C-NMR chemical shifts, Table S4: Experimental values of ^13^C-NMR *δ* chemical shifts of dermacozines E (**8**), F (**9**), G (**9**), and O (**2**) for multiple regression and *t*-test, Figure S43: Multiple regression analysis between the experimental ^13^C-NMR *δ* values of dermacozines E (**8**), F (**9**), G (**9**) as independent variables and those of dermacozine O (**2**) as dependent variables, Table S5: Residuals between the observed and predicted values of ^13^C-NMR *δ* of dermacozines E (**8**), F (**9**), G (**9**) as independent variables and those of dermacozine O (**2**) as dependent variables, Table S6: Comparison between experimental and the ACD Labs calculated ^13^C-NMR *δ* chemical shift values of dermacozines P (**3**) (Neural Network Algorithm, DMSO-*d*_6_), Figure S44: Linear regression graphics between the experimental ^13^C-NMR *δ* chemical shifts of dermacozine P (**3**) and the ACD Labs calculated ^13^C-NMR *δ* values (Neural Network Algorithm, DMSO-*d*_6_), Table S7: Absolute error and the standard deviation of the error from the mean between the calculated and the experimental dermacozine P (**3**) ^13^C-NMR chemical shifts, Figure S45: Evaluation of the cytotoxic activity of dermacozine N (**1**) against human (A) Melanoma (A2058) and (B) Hepatocellular carcinoma (HepG2) cell lines (IC_50_ graphs), Table S8: Experimental NMR spectroscopic data for dermacozine N (**1**) with HMBC, COSY, and NOESY correlations (800 MHz, DMSO-*d*_6_), Table S9: Experimental NMR spectroscopic data for dermacozine O (**2**) with HMBC and COSY correlations (800 MHz, DMSO-*d*_6_), Table S10: Experimental NMR spectroscopic data for dermacozine P (**3**) with HMBC and COSY correlations (600 MHz, DMSO-*d*_6_), Figure S46: Workflow of dermacozine N (**1**) structure determination, Figure S47: Workflow of dermacozine O (**2**) structure determination, Figure S48: Workflow of dermacozine P (**3**) structure determination, Theme S1: Dermacozine N (**1**) H-9 and NH_2_-12 distance (modeled with Chemdraw, Chem 3D), Theme S2: TLC plate of the initial FD fraction following Kupchan liquid–liquid partitioning, showing the colorful bands where the dermacozines were isolated from (left) and the same TLC plate under UV 360 nm light (right), Theme S3: HPLC chromatogram for the isolation of dermacozine O (**2**), Theme S4: HPLC chromatogram for the isolation of dermacozine P (**3**).
