# Supplementary material for: Dermacozine N, the First Natural Linear Pentacyclic Oxazinophenazine with UV–Vis Absorption Maxima in the Near Infrared Region, along with Dermacozines O and P Isolated from the Mariana Trench Sediment Strain Dermacoccus abyssi MT 1.1T
Source: Mar Drugs. 2021 Jun 3;19(6):325. doi: 10.3390/md19060325 (PMC8226881; doi:10.3390/md19060325)
Supplement: Supplementary file 1 [file marinedrugs-19-00325-s001.zip › marinedrugs-1225744-SI.pdf]

marine drugs

Supporting Information

Dermacozine N, the First Natural Linear Pentacyclic Oxazinophenazine with UV-Vis Absorption Maxima in the Near Infrared Region, along with Dermacozines O and P Isolated from the Mariana Trench Sediment Strain *Dermacoccus abyssi* MT 1.1^{T}

Bertalan Juhasz [1], Dawrin Pech-Puch [2], Jioji N. Tabudravu [3], Bastien Cautain [4], Fernando Reyes [4], Carlos Jiménez [5], Kwaku Kyeremeh [6] and Marcel Jaspars [1]*

1 Marine Biodiscovery Centre, Department of Chemistry, University of Aberdeen, Old Aberdeen, Scotland, AB24 3UE, UK; r01bj16@abdn.ac.uk
2 Departamento de Biología Marina, Universidad Autónoma de Yucatán, Km. 15.5, carretera Mérida-Xmatkuil, A.P. 4-116 Itzimná, C.P. 97100 Mérida, Yucatán, México; dawrin.j.pech@udc.es
3 School of Natural Sciences, Faculty of Science and Technology, University of Central Lancashire, Preston, PR1 2HE, UK; JTabudravu@uclan.ac.uk
4 Fundación MEDINA, Centro de Excelencia en Investigación de Medicamentos Innovadores en Andalucía Avda. del Conocimiento 34, Edificio Centro de Desarrollo Farmacéutico y Alimentario, Parque Tecnológico de Ciencias de la Salud 18016 Granada, Spain; cautainbastien@gmail.com (B.C.); fernando.reyes@medinaandalucia.es (F.R.)
5 Centro de Investigacións Científicas Avanzadas (CICA) e Departmento de Química, Facultade de Ciencias, AE CICA-INIBIC, Universidad da Coruña, 15071 A Coruña, Spain; carlos.jimenez@udc.es
6 Marine and Plant Research Laboratory of Ghana, Department of Chemistry, School of Physical and Mathematical Sciences, University of Ghana, P.O. Box LG 56, Legon-Accra, Ghana; kkyeremeh@ug.edu.gh
* Correspondence: m.jaspars@abdn.ac.uk, Tel.: +44-1224-272-895

marine drugs

Bertalan Juhasz et al.

Contents

Figure S1. LC-MS analysis of dermacozine N (**1**) (qTOF, Bruker)....................................... 6

Figure S2. MS/MS of dermacozine N (**1**) (qTOF, Bruker) 6

Figure S3A. Proposed MS fragmentation pathway of dermacozine N (**1**) showing loss of a (-ON(C_6H_4)-) fragment. 7

Figure S3B. Mass Error between the calculated (Chemdraw modelled) and experimental (qTOF) *m*/*z* ratio of dermacozine N (**1**) 7

Figure S4. UV-Vis spectrum of dermacozine N (**1**) in EtOH 8

Figure S6. Dermacozine N (**1**) ^{1}H-NMR spectrum (800 MHz, DMSO-d_6)............................ 9

Figure S7. ^{1}H-^{13}C HSQC spectrum of dermacozine N (**1**) (800 MHz, DMSO-d_6) 9

Figure S8. ^{1}H-^{13}C HMBC (magnitude mode) spectrum of dermacozine N (**1**)) (800 MHz, DMSO-d_6) Inlets show the H-2 to C-11 carbonyl correlation (top left, phase sensitive mode), the *ortho*-substituted **D**-ring (top right, magnitude mode) and N-methyl C4a, C5a correlation (bottom right, phase sensitive mode 10

Figure S9. ^{1}H-^{13}C HMBC spectrum of dermacozine N (**1**) with 2 Hz Coupling (800 MHz, DMSO-d_6).. 10

Figure S10. ^{1}H-^{1}H COSY spectrum of dermacozine N (**1**) (800 MHz, DMSO-d_6) 11

Figure S11. ^{1}H-^{1}H NOESY spectrum of dermacozine N (**1**) (800 MHz, DMSO-d_6) (Inlets show the N-methyl and H-4 (top), and NH_2-12 to H-9 (middle and lower) correlations) 12

Figure S12. ^{1}H-^{15}N HMBC spectrum of dermacozine N (**1**) (800 MHz, DMSO-d_6) 13

Figure S13. LC-MS analysis of dermacozine O (**2**) (qTOF, Bruker).................................... 13

Figure S14. UV-Vis spectrum of dermacozine O (**2**) in EtOH 14

Figure S15. IR spectrum of dermacozine O (**2**) in EtOH 14

Figure S16. ^{1}H-NMR spectrum of dermacozine O (**2**) (800 MHz, DMSO-d_6).................... 15

Figure S17. ^{1}H-^{13}C HSQC spectrum of dermacozine O (**2**) (800 MHz, DMSO-d_6) 15

Figure S18. ^{1}H-^{13}C HMBC spectrum of dermacozine O (**2**) (800 MHz, DMSO-d_6) (Inlets show NH-14 and C-6, C-16 correlations (top), N-methyl hydrogen atom to C4a and C5a correlations (middle), H2 to C-11 carbonyl correlation (bottom)) 16

Figure S19. Long Range ^{1}H-^{13}C HMBC correlations of Dermacozine O (**2**) with *J* = 2Hz (800 MHz, DMSO-d_6) 16

Figure S20. ^{1}H-^{1}H COSY spectrum of dermacozine O (**2**) (800 MHz, DMSO-d_6) 17

Figure S21. ^{1}H-^{15}N HMBC spectrum of dermacozine O (**2**) (800 MHz, DMSO-*d*6)...........17

Figure S22. UV-Vis spectrum of dermacozine P (**3**) in EtOH...........18

Figure S23. IR spectrum of dermacozine P (**3**) in EtOH...........18

Figure S24. LC-MS analysis of dermacozine P (**3**) (Orbitrap, Xcalibur) 19

Figure S25. MS/MS analysis of dermacozine P (**3**) (Orbitrap, Xcalibur)...........19

Figure S26. ^{1}H-NMR spectrum of dermacozine P (**3**) (600 MHz, DMSO-*d*6)...........20

Figure S27. ^{1}H-^{13}C HSQC spectrum of dermacozine P (**3**) (600 MHz, DMSO-*d*6)...........20

Figure S28. ^{1}H-^{13}C HMBC spectrum of dermacozine P (**3**) (600 MHz, DMSO-*d*6)...........21

Figure S29. ^{1}H-^{1}H COSY spectrum of dermacozine P (**3**) (600 MHz, DMSO-*d*6) 21

Figure S30. 1D-NOESY spectrum of dermacozine P (**3**) (600 MHz, DMSO-*d*6) from irradiation of the signal at 8.03 ppm...........22

Figure S31.^{13}C-NMR δ calculated values of dermacozine A-D (**4-7**) using the ACD Labs software with Neural Network Algorhitm Algorithm, solvent DMSO-*d*6...........22

Figure S32. ^{13}C-NMR δ calculated values of dermacozine E-H (**8-11**) using the ACD Labs Software with Neural Network Algorhitm Algorithm, solvent DMSO-*d*6 23

Figure S33. ^{13}C-NMR δ calculated values of dermacozine I-J (**12-13**) and dermacozine M-P (**14**, **1-3**) using the ACD Labs Software with Neural Network Algorhitm Algorithm, solvent DMSO-*d*6 23

Figure S34. ^{13}C-NMR δ calculated values of possible structures (**A**-**F**) of dermacozine N (**1**), using the ACD Labs Software with Neural Network Algorhitm Algorithm, solvent DMSO-*d*6...........24

Figure S35. ^{13}C-NMR δ calculated values of possible structures (**G**-**L**) of dermacozine N (**1**), using the ACD Labs Software with Neural Network Algorithm, solvent DMSO-*d*6 24

Figure S36. ^{13}C-NMR δ calculated values of possible structures (**M**-**R**) of dermacozine N (**1**), using the ACD Labs Software with Neural Network Algorithm, solvent DMSO-*d*6 25

Figure S37. ^{13}C-NMR δ calculated values of possible structures (**S**-**U**) of dermacozine N (**1**), using the ACD Labs Software with Neural Network Algorithm, solvent DMSO-*d*6 25

Figure S38. ^{13}C-NMR δ calculated values of possible structures (**V** and **W**) of dermacozine N (**1**), using the ACD Labs Software with Neural Network Algorithm, solvent DMSO-*d*6 26

Table S1. Comparison between calculated vs. experimental ^{13}C-NMR δ values of dermacozines A-J (**4-13**) 27

Figure 39. Linear regression graphics between ACD Labs (Neural Network Algorithm) calculated vs. experimental ^{13}C-NMR δ values of dermacozine A-H (**4-11**)...........28

Table S2. Comparison between the experimental ^{13}C-NMR δ values of dermacozine N (**1**) vs. the calculated ones of possible structures **A**, **D**, **E**, **G**, **H**, **I**, **J**, **K**, **N**, **P**, **Q**, **V**, **W** by ACD Labs (Neural Network Algorithm, DMSO-d_6) 30

Figure S41. Linear regression graphics between ^{13}C-NMR δ experimental values of dermacozine N (**1**) vs. the ACD Labs calculated ones (Neural Network Algorithm, DMSO-d_6) (**4-13**)........ 31

Figure S42. Linear regression graphics between ^{13}C-NMR δ experimental values of dermacozine N (**1**) vs. the ACD Labs (Neural Network Algorithm, DMSO-d_6) calculated ones for possible structures **N**, **P**, **Q**, **V**, **W** 32

Table S3. Absolute Error and the standard deviation of the error from the mean between the calculated (Structure **W**) and the experimental dermacozine N (**1**) ^{13}C-NMR chemical shifts 33

Table S4. Experimental values of ^{13}C-NMR δ chemical shifts of dermacozines E (**8**), F (**9**), G (**9**) and O (**2**) for multiple regression and t-test 34

Figure S43. Multiple regression analysis between the experimental ^{13}C-NMR δ values of dermacozines E (**8**), F (**9**), G (**9**) as independent variables and those of dermacozine O (**2**) as dependent variables 37

Table S5. Residuals between the observed and predicted values of ^{13}C-NMR δ of dermacozines E (**8**), F (**9**), G (**9**) as independent variables and those of dermacozine O (**2**) as dependent variables 38

Table S6. Comparison between experimental and the ACD Labs calculated ^{13}C-NMR δ chemical shift values of dermacozines P (**3**) (Neural Network Algorithm, DMSO-d_6)....39

Figure S44. Linear regression graphics between the experimental ^{13}C-NMR δ chemical shifts of dermacozine P (**3**) and the ACD Labs calculated ^{13}C-NMR δ values (Neural Network Algorithm, DMSO-d_6) 40

Table S7. Absolute Error and the standard deviation of the error from the mean between the calculated and the experimental dermacozine P (**3**) ^{13}C-NMR chemical shifts 41

Figure S45. Evaluation of the cytotoxic activity of dermacozine N (**1**) against human A) Melanoma (A2058) and B) Hepatocellular carcinoma (HepG2) cell lines (IC_{50} graphs) .. 42

Table S8. Experimental NMR spectroscopic data for dermacozine N (**1**) with HMBC, COSY and NOESY correlations (800 MHz, DMSO-d_6) 43

Table S9. Experimental NMR spectroscopic data for dermacozine O (**2**) with HMBC and COSY correlations (800 MHz, DMSO-d_6)........ 44

Table S10. Experimental NMR spectroscopic data for dermacozine P (**3**) with HMBC and COSY correlations (600 MHz, DMSO-d_6)........ 45

Figure S46. Workflow of dermacozine N (**1**) structure determination 46

Figure S47. Workflow of dermacozine O (**2**) structure determination. 47

Figure S48. Workflow of dermacozine P (**3**) structure determination 48

Theme S1. Dermacozine N (**1**) H-9 and NH_2 -12 distance (Modelled with Chemdraw, Chem 3D).. 49

Theme S2. TLC plate of the initial FD fraction following Kupchan liquid-liquid partitioning, showing the colorful bands where the dermacozines were isolated from (left) and the same TLC plate under UV 360 nm light (right).. 50

Theme S3. HPLC chromatogram for the isolation of dermacozine O (**2**)......................... 50

Theme S4. HPLC chromatogram for the isolation of dermacozine P (**3**) 51

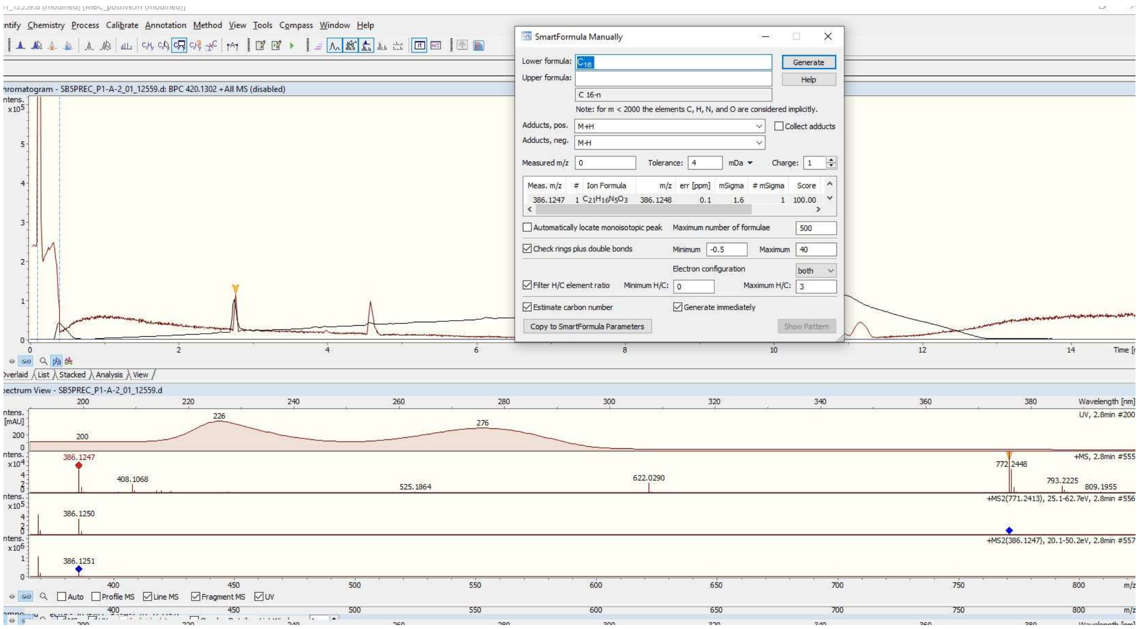

Figure S1. LC-MS analysis of dermacozine N (**1**) (qTOF, Bruker)

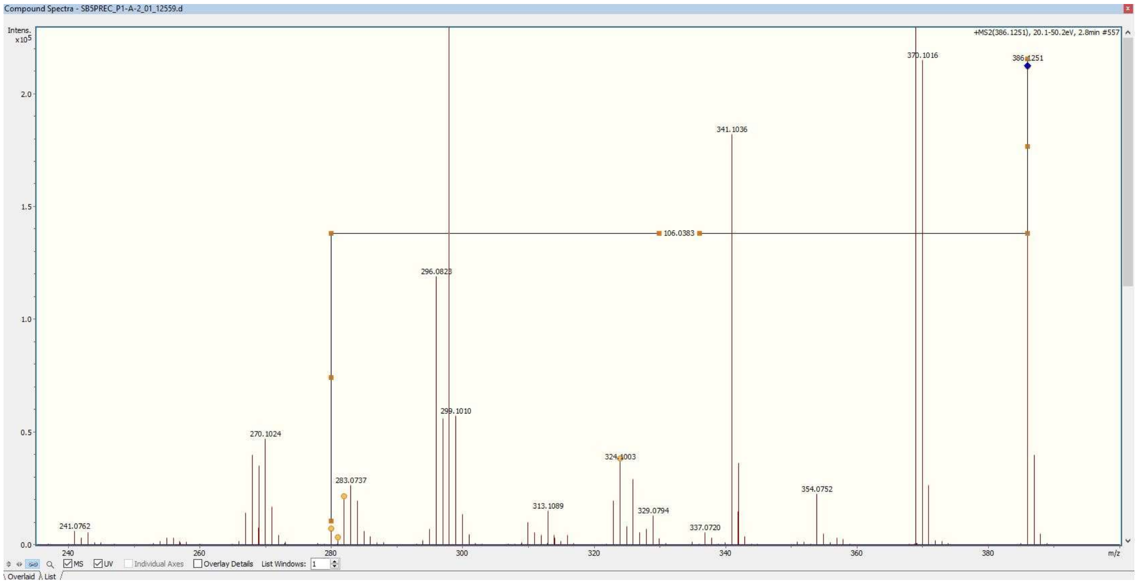

Figure S2. MS/MS of dermacozine N (**1**) (qTOF, Bruker)

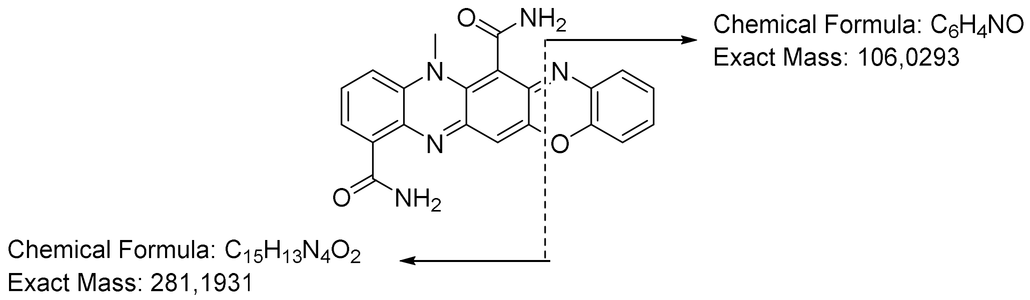

Figure S3A. Proposed MS fragmentation pathway of dermacozine N (**1**) showing loss of a (-ON(C_6H_4)-) fragment.

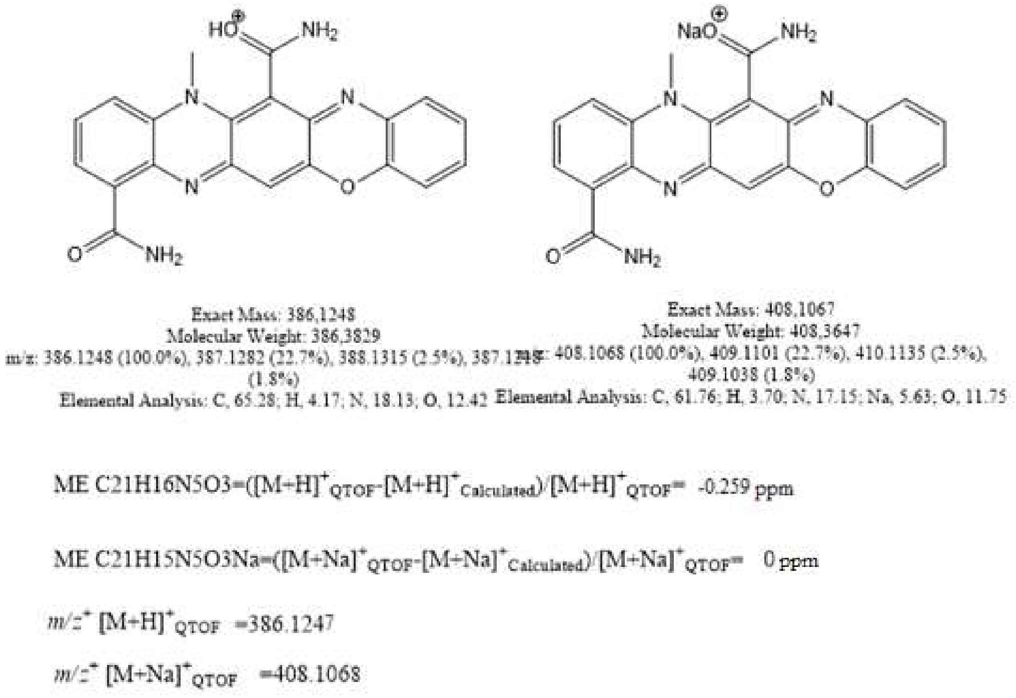

Figure S3B. Mass Error between the calculated (Chemdraw modelled) and experimental (qTOF) *m*/*z* ratio of dermacozine N (**1**)

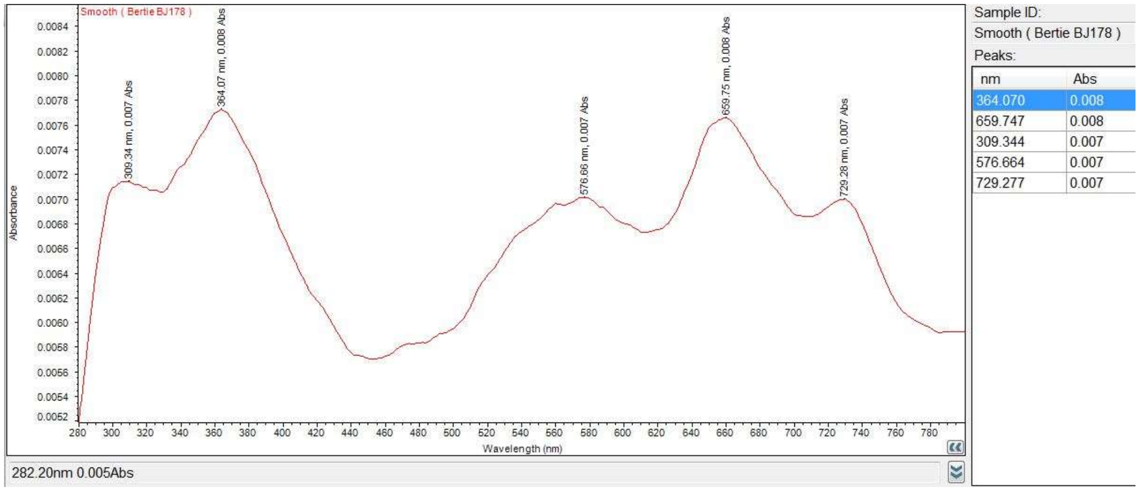

Figure S4. UV-Vis spectrum of dermacozine N (**1**) in EtOH

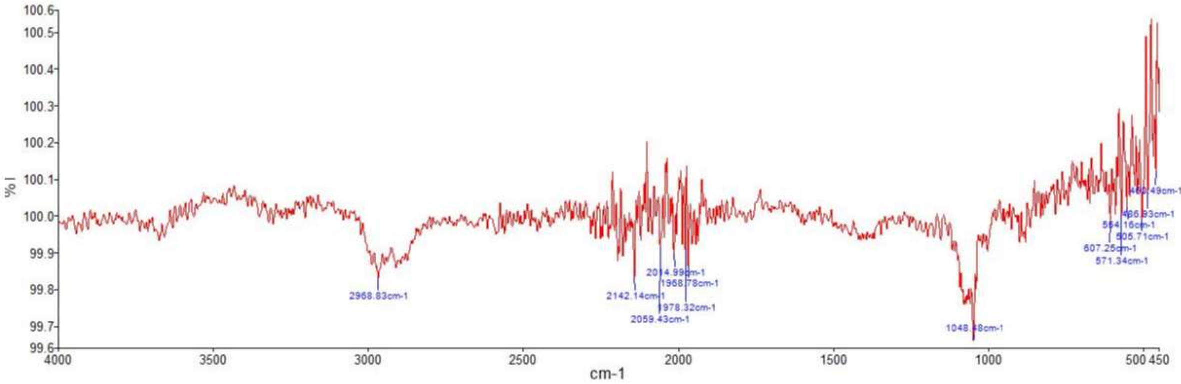

Figure S5. IR spectrum of dermacozine N (**1**) in EtOH

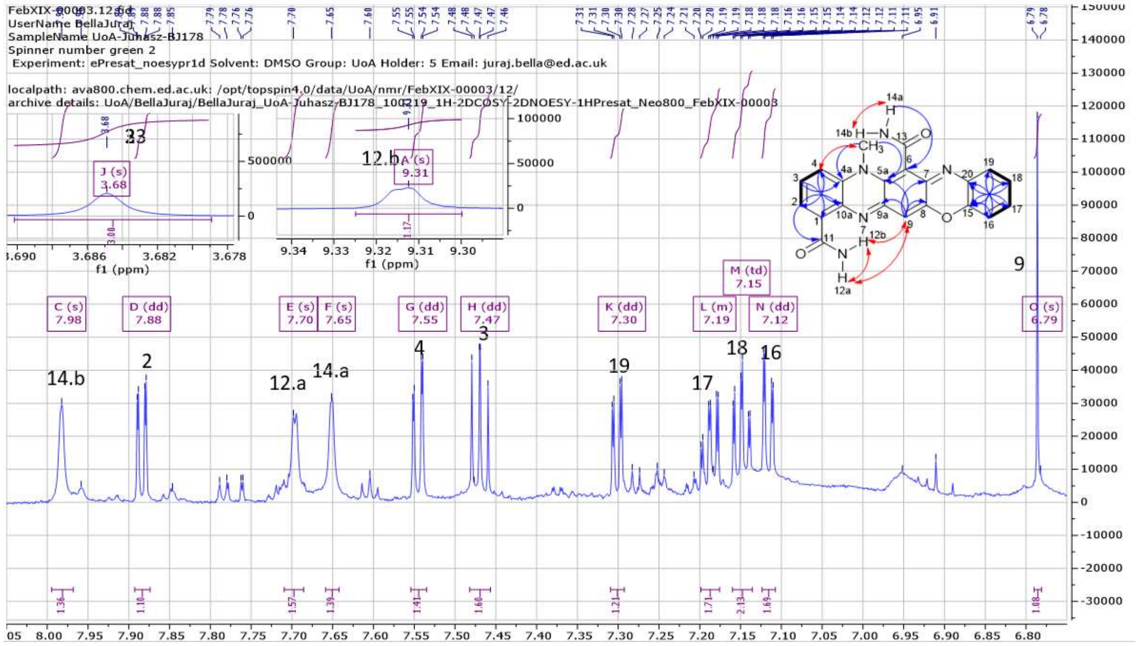

Figure S6. Dermacozine N (**1**) ^{1}H-NMR spectrum (800 MHz, DMSO-d_6)

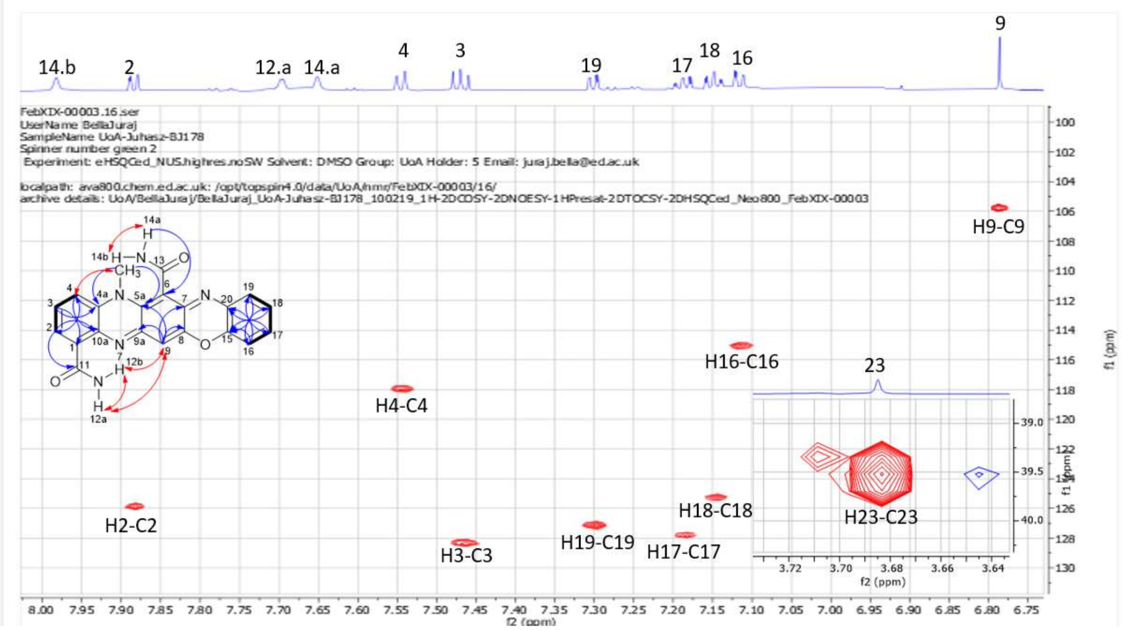

Figure S7. ^{1}H-^{13}C HSQC spectrum of dermacozine N (**1**) (800 MHz, DMSO-d_6)

(Inlet shows the N-methyl group)

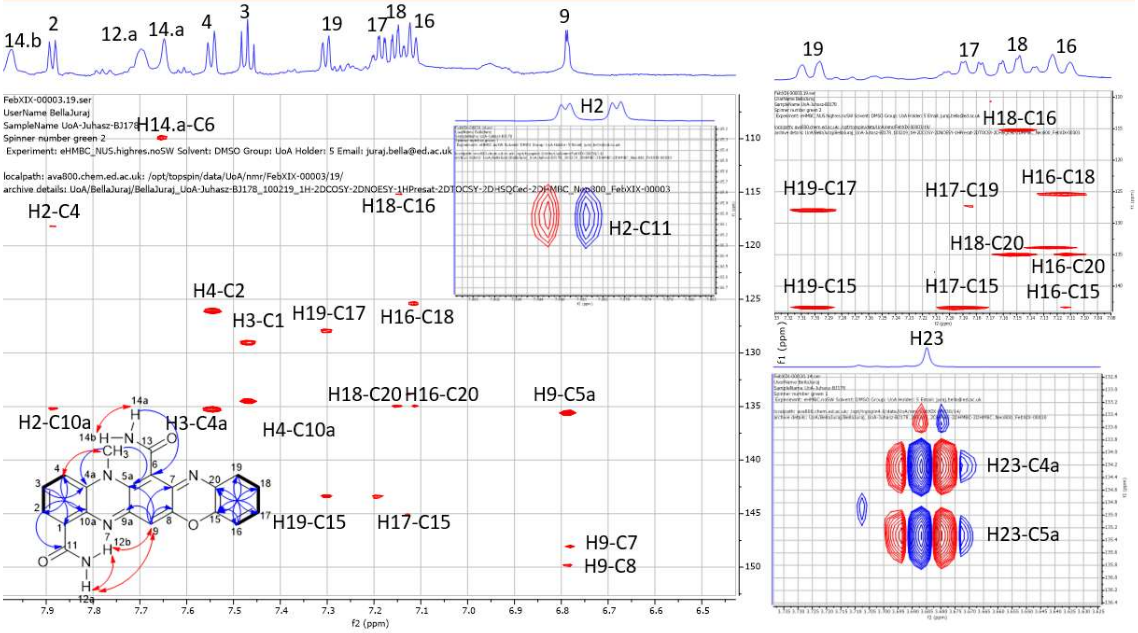

Figure S8. ^{1}H-^{13}C HMBC (magnitude mode) spectrum of dermacozine N (**1**)) (800 MHz, DMSO-d_6) Inlets show the H-2 to C-11 carbonyl correlation (top left, phase sensitive mode), the *ortho*-substituted **D**-ring (top right, magnitude mode) and N-methyl C4a, C5a correlation (bottom right, phase sensitive mode

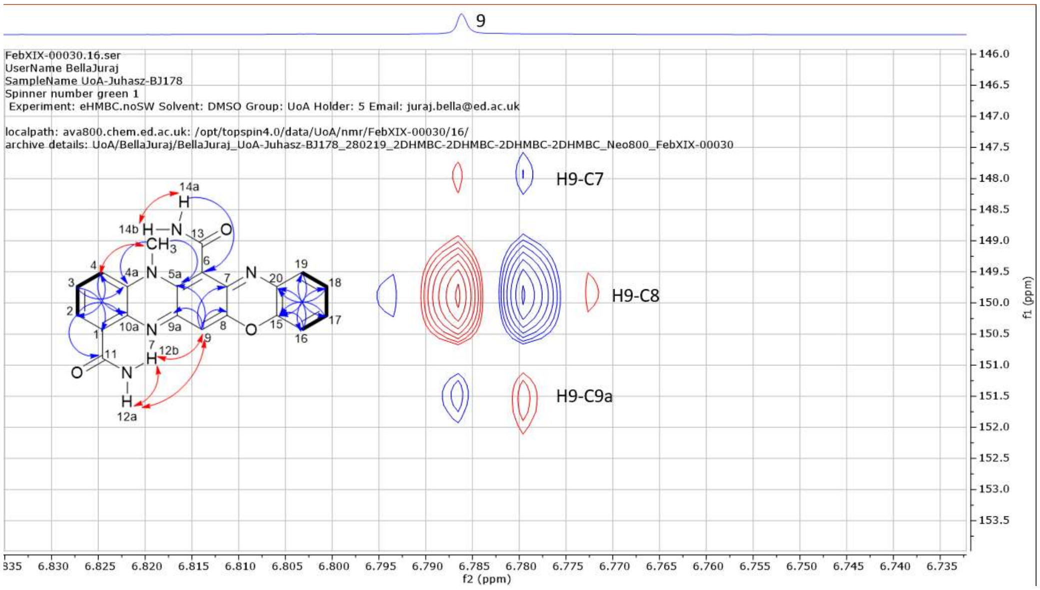

Figure S9. ^{1}H-^{13}C HMBC spectrum of dermacozine N (**1**) with 2 Hz Coupling (800 MHz, DMSO-d_6)

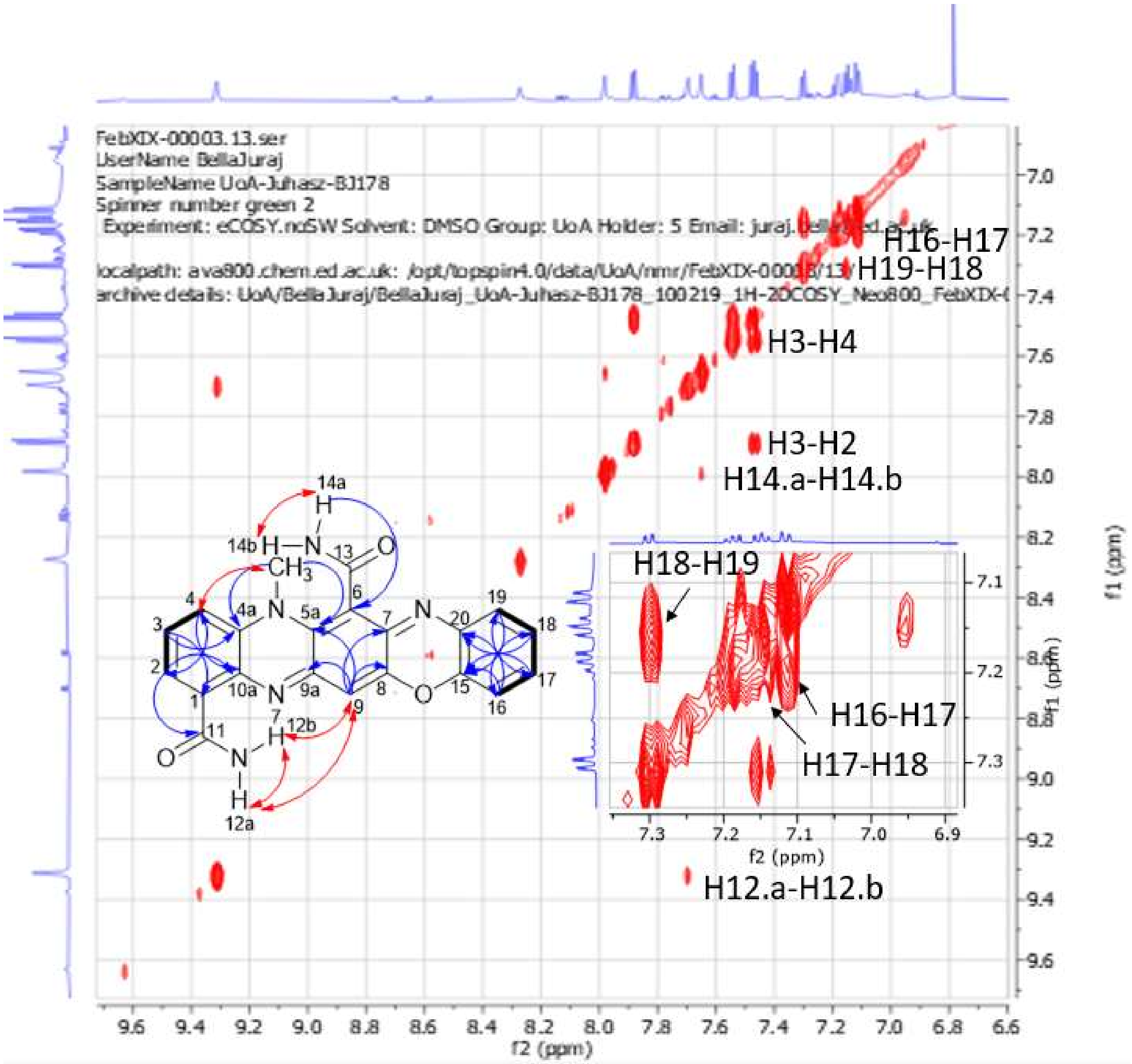

Figure S10. ^1H-^1H COSY spectrum of dermacozine N (**1**) (800 MHz, DMSO-*d*6)

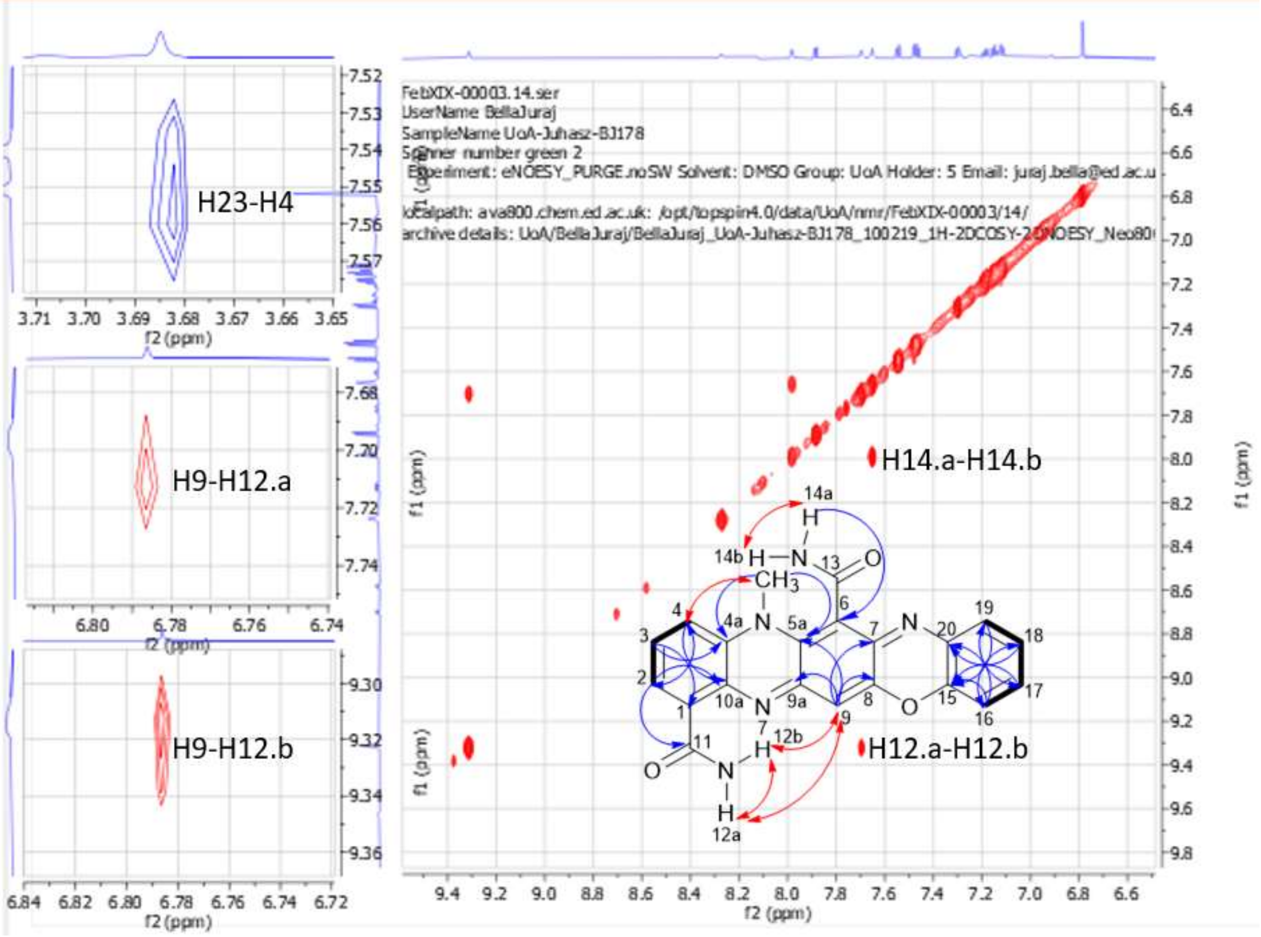

Figure S11. ^{1}H-^{1}H NOESY spectrum of dermacozine N (**1**) (800 MHz, DMSO-d_6)

(Inlets show the N-methyl and H-4 (top), and NH_2-12 to H-9 (middle and lower) correlations)

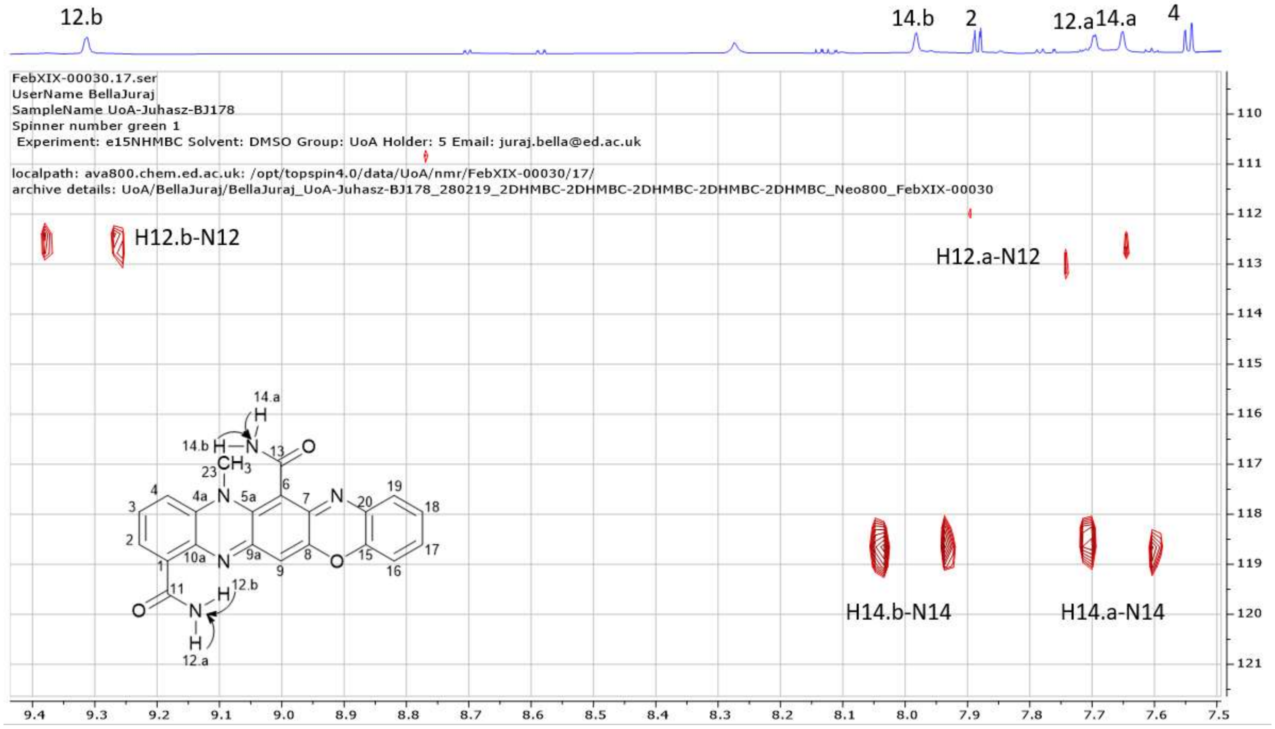

Figure S12. ^1H-^{15}N HMBC spectrum of dermacozine N (**1**) (800 MHz, DMSO-d_6)

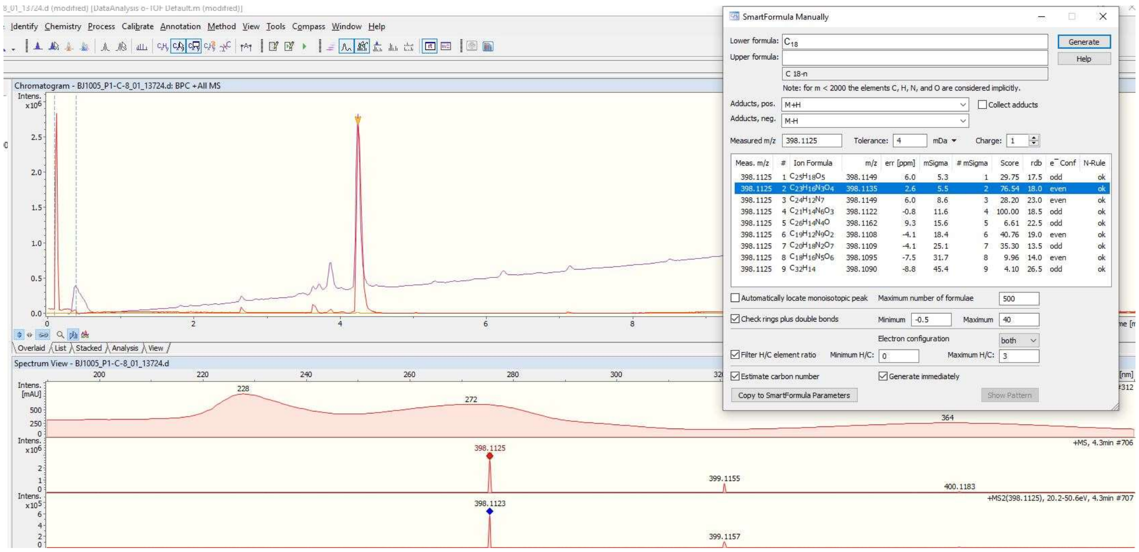

Figure S13. LC-MS analysis of dermacozine O (**2**) (qTOF, Bruker)

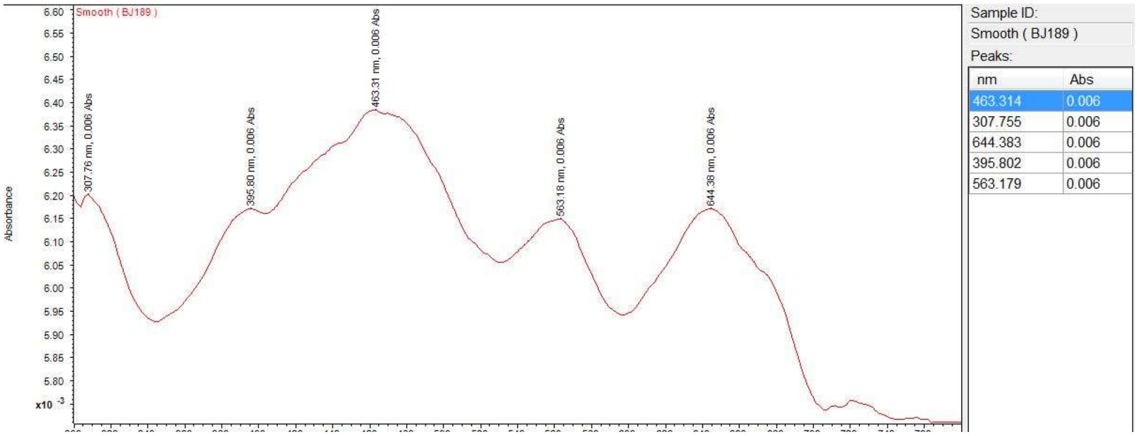

Figure S14. UV-Vis spectrum of dermacozine O (**2**) in EtOH

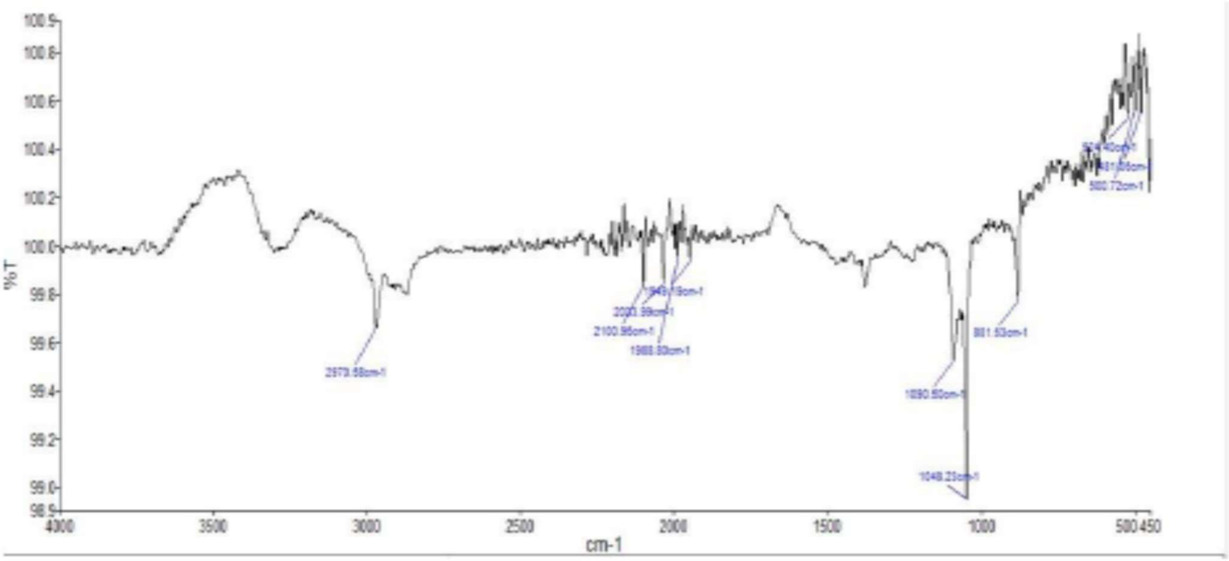

Figure S15. IR spectrum of dermacozine O (**2**) in EtOH

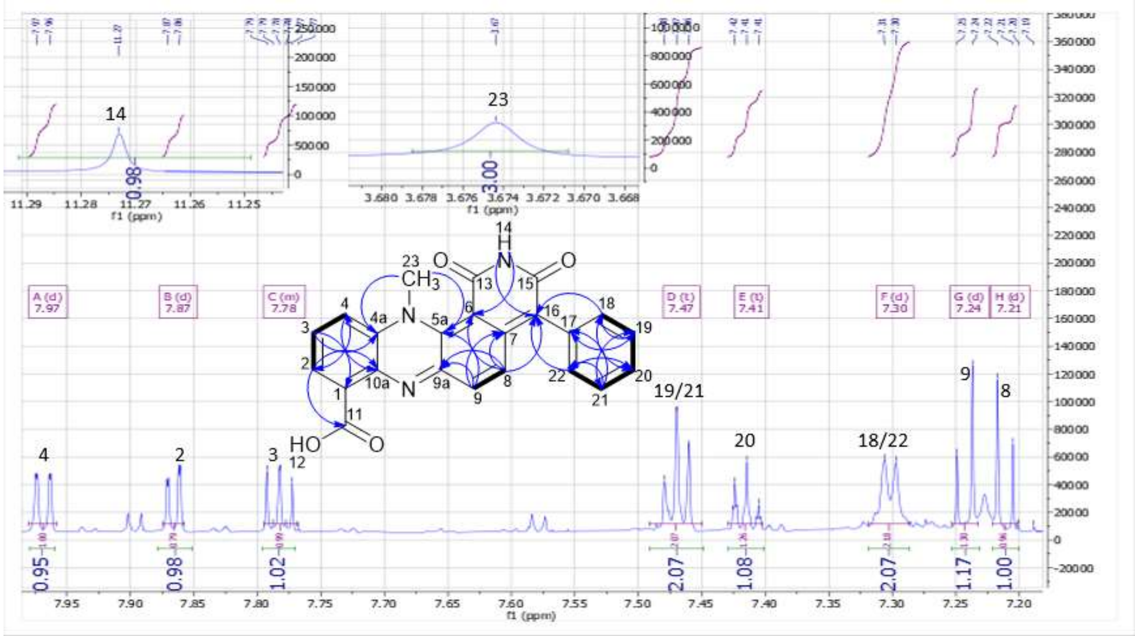

Figure S16. ^{1}H-NMR spectrum of dermacozine O (**2**) (800 MHz, DMSO-d_6)

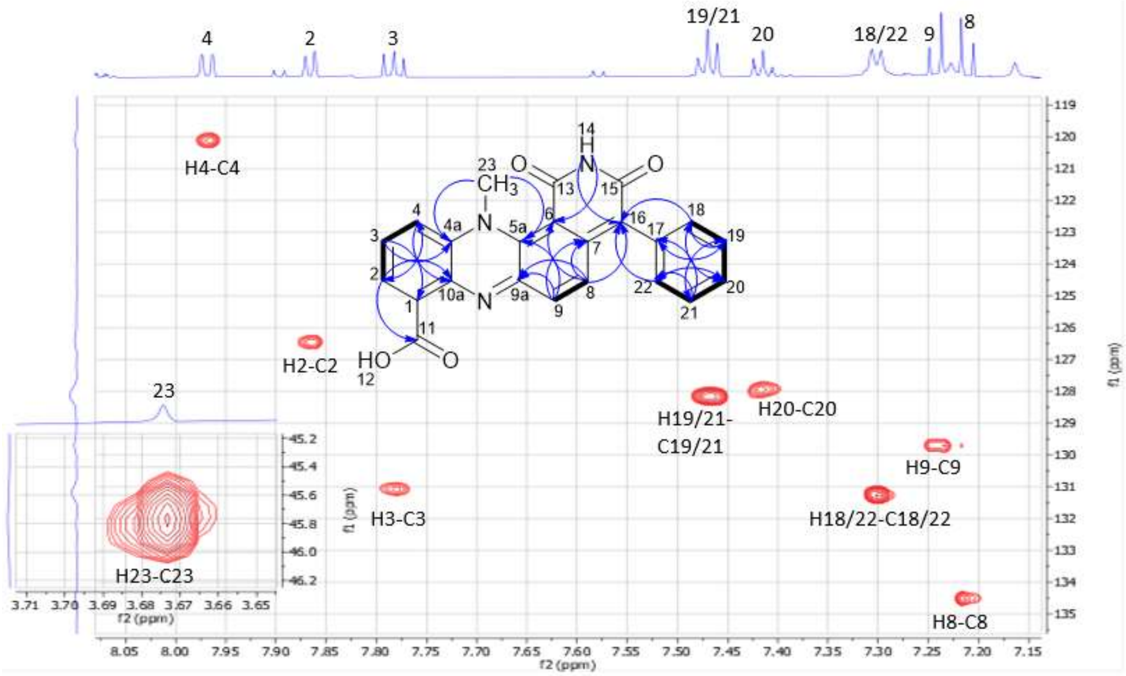

Figure S17. ^{1}H-^{13}C HSQC spectrum of dermacozine O (**2**) (800 MHz, DMSO-d_6)

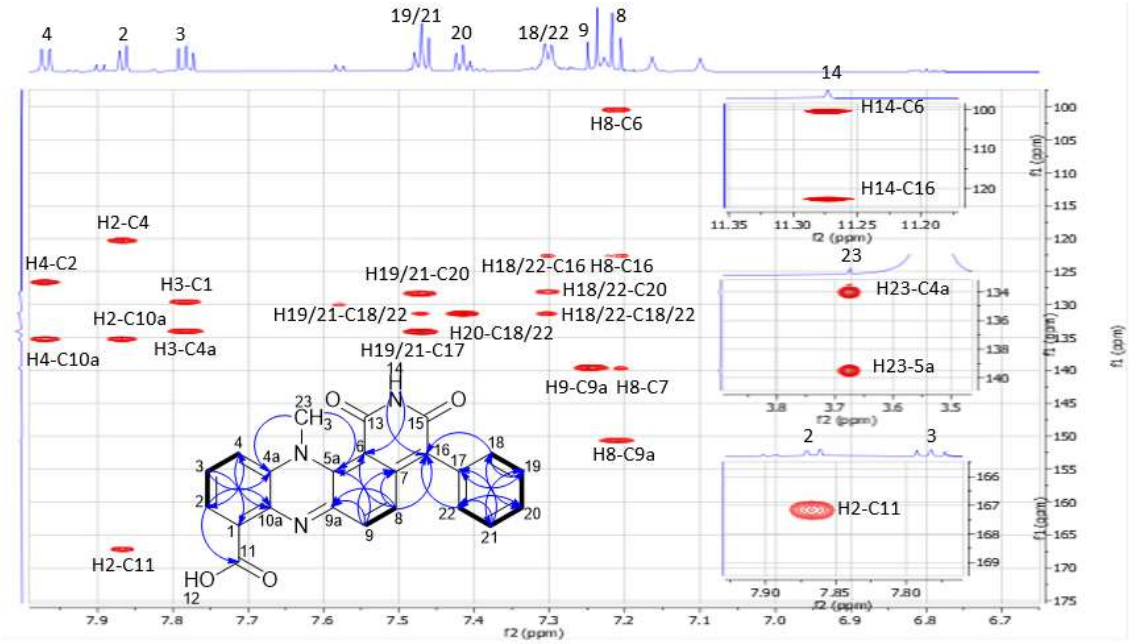

Figure S18. ^{1}H-^{13}C HMBC spectrum of dermacozine O (**2**) (800 MHz, DMSO-*d*6)
(Inlets show NH-14 and C-6, C-16 correlations (top), N-methyl hydrogen atom to C4a and C5a correlations (middle), H2 to C-11 carbonyl correlation (bottom))

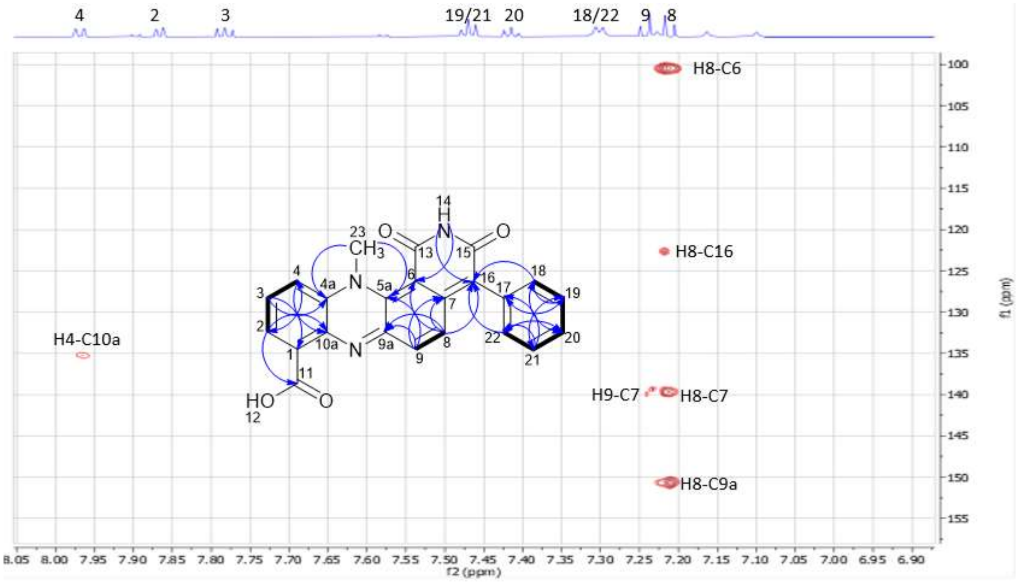

Figure S19. Long Range ^{1}H-^{13}C HMBC correlations of Dermacozine O (**2**) with *J* = 2Hz (800 MHz, DMSO-*d*6)

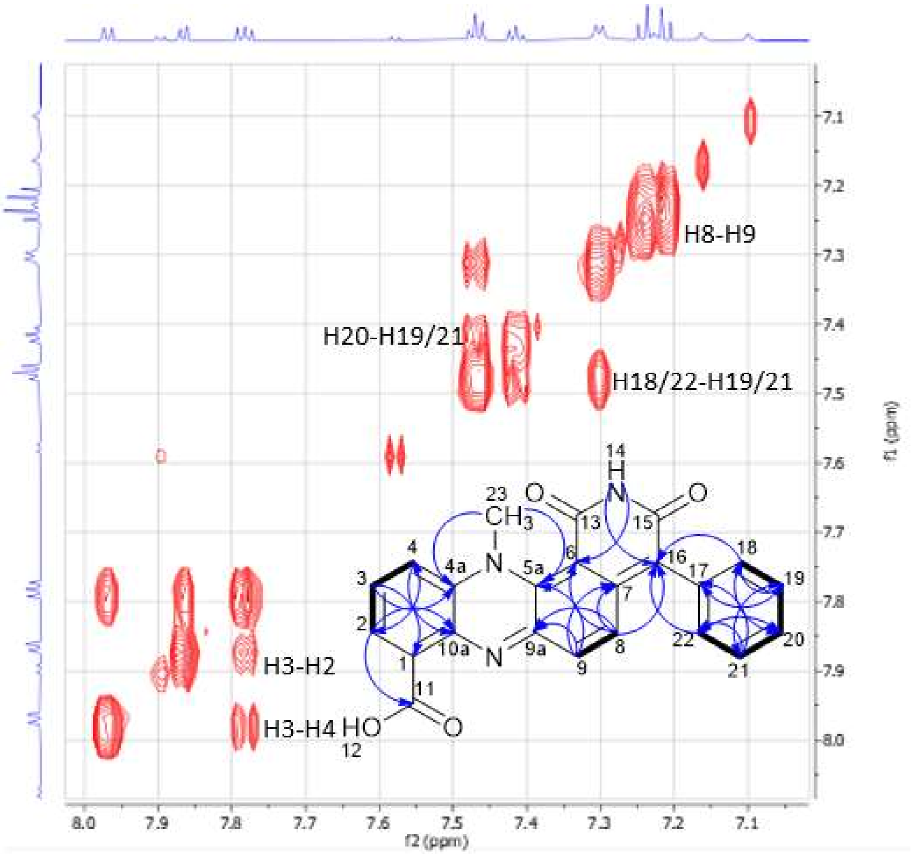

Figure S20. ^{1}H-^{1}H COSY spectrum of dermacozine O (**2**) (800 MHz, DMSO-d_6)

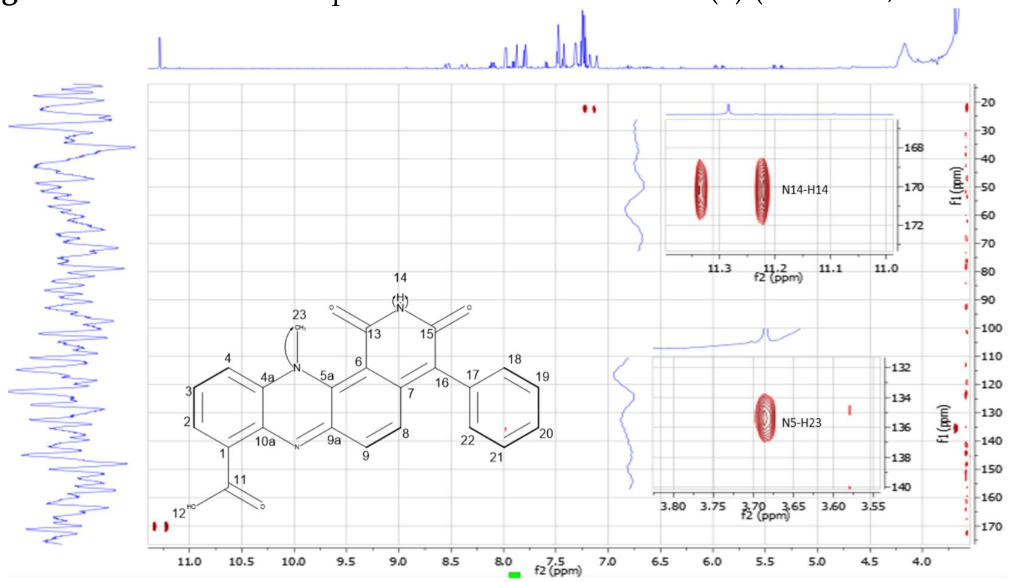

Figure S21. ^{1}H-^{15}N HMBC spectrum of dermacozine O (**2**) (800 MHz, DMSO-d_6)

Inlets show the N-methyl nitrogen (top) and the cyclic carboximide nitrogen (bottom)

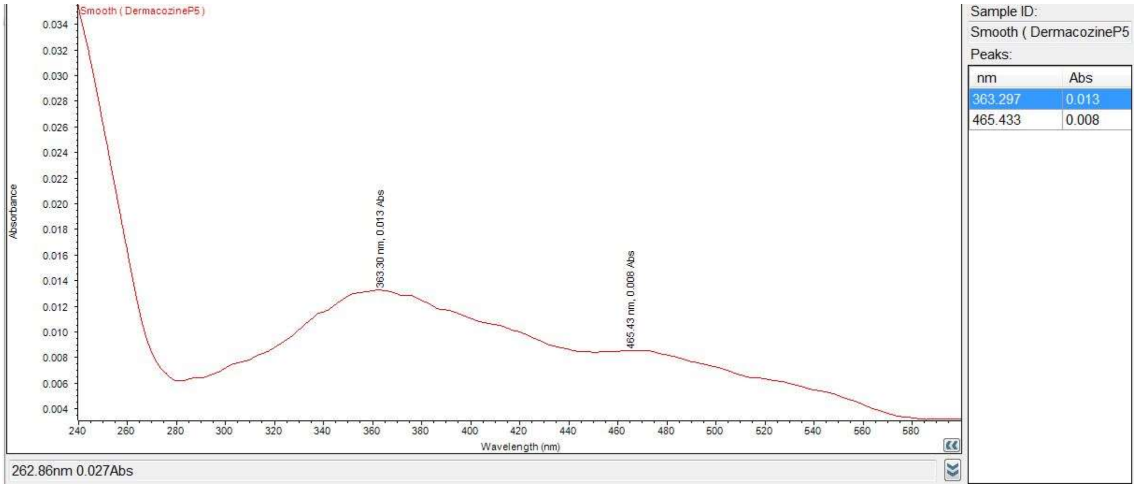

Figure S22. UV-Vis spectrum of dermacozine P (**3**) in EtOH

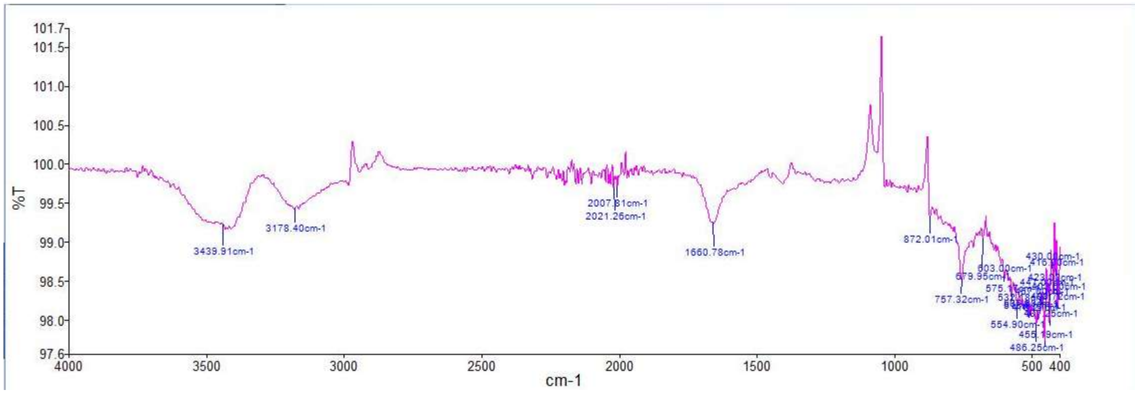

Figure S23. IR spectrum of dermacozine P (**3**) in EtOH

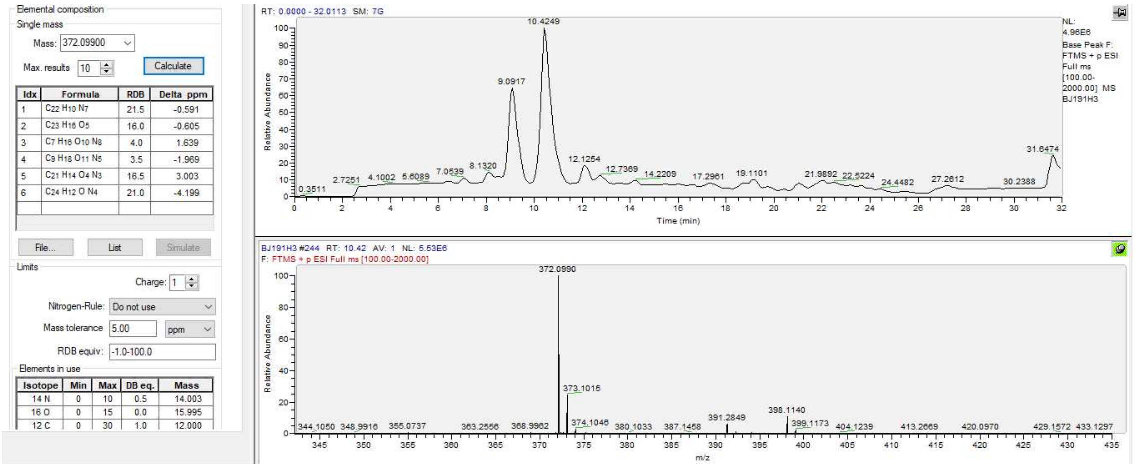

Figure S24. LC-MS analysis of dermacozine P (**3**) (Orbitrap, Xcalibur)

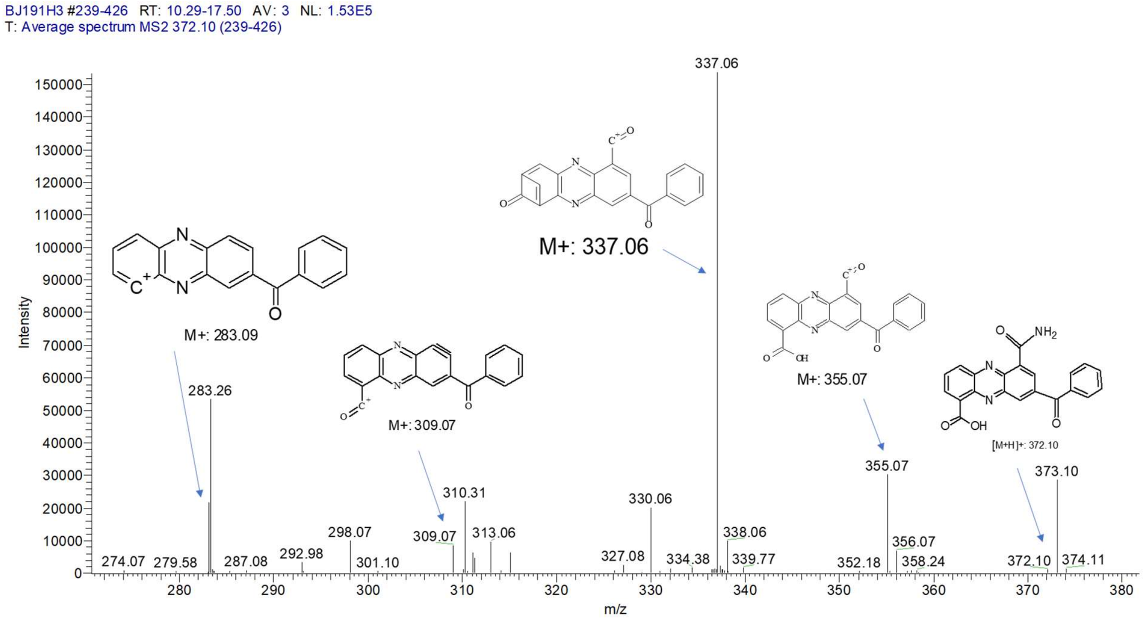

Figure S25. MS/MS analysis of dermacozine P (**3**) (Orbitrap, Xcalibur)

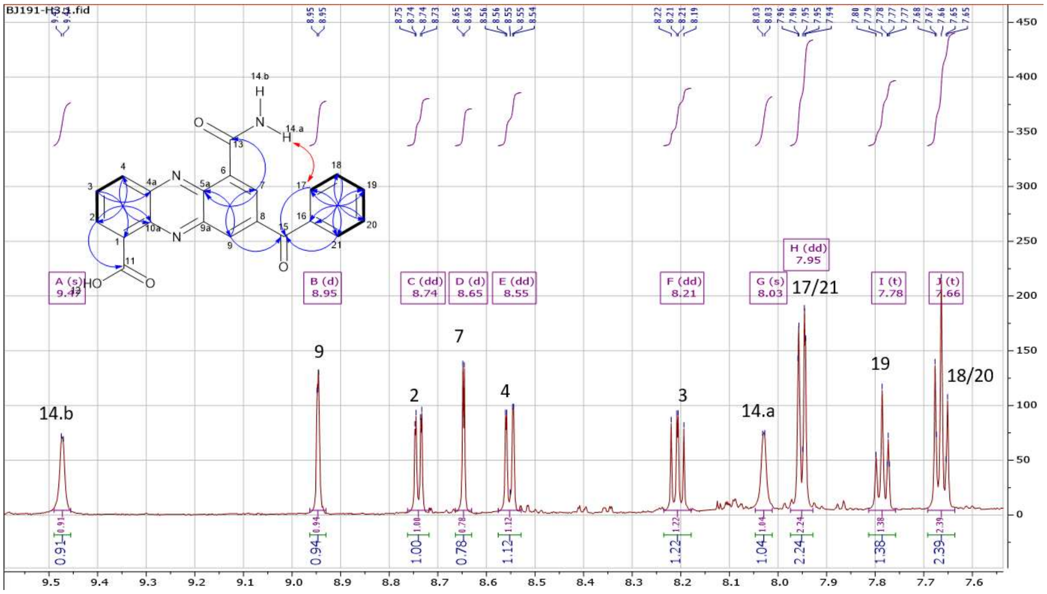

Figure S26. ^{1}H-NMR spectrum of dermacozine P (**3**) (600 MHz, DMSO-d_6)

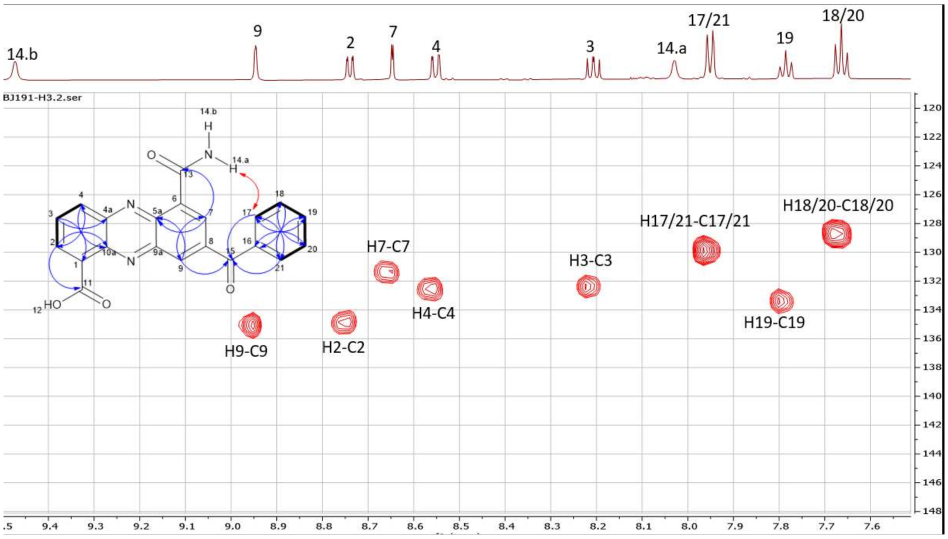

Figure S27. ^{1}H-^{13}C HSQC spectrum of dermacozine P (**3**) (600 MHz, DMSO-d_6)

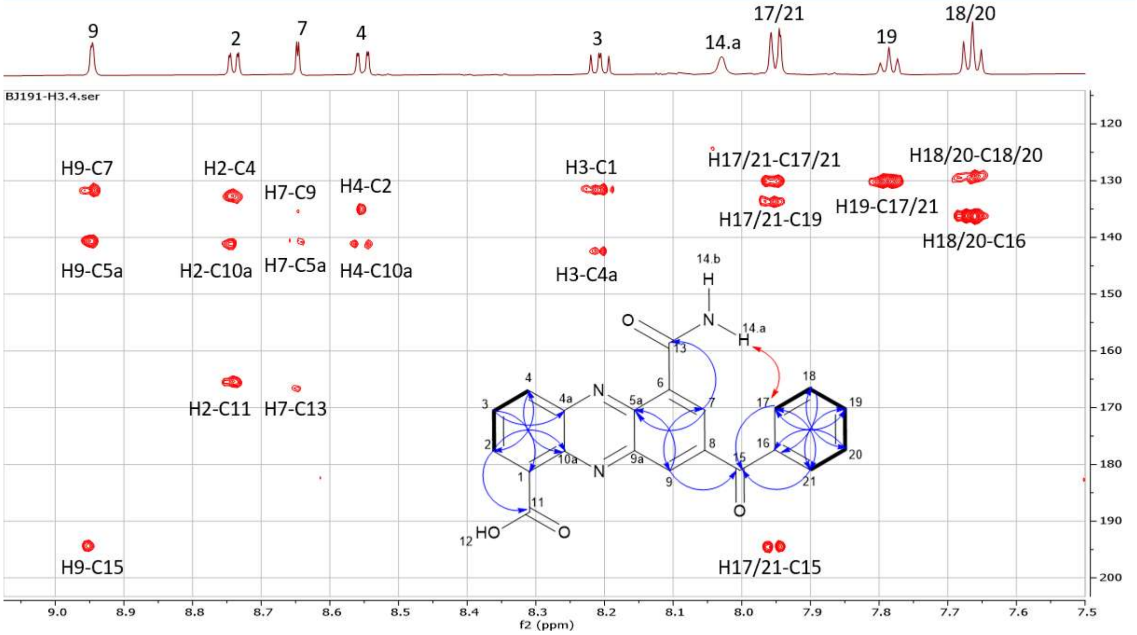

Figure S28. ^{1}H-^{13}C HMBC spectrum of dermacozine P (**3**) (600 MHz, DMSO-d_6)

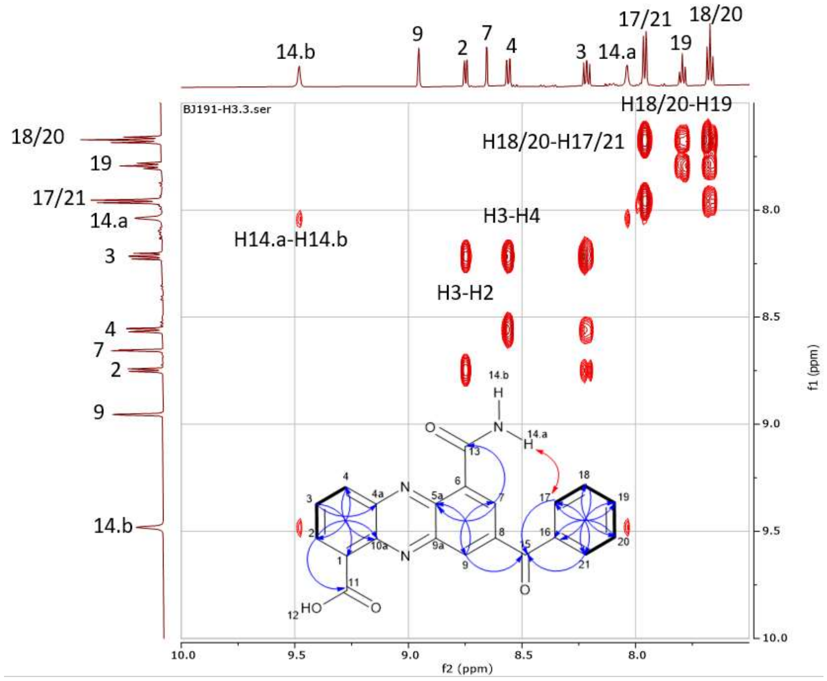

Figure S29. ^{1}H-^{1}H COSY spectrum of dermacozine P (**3**) (600 MHz, DMSO-d_6)

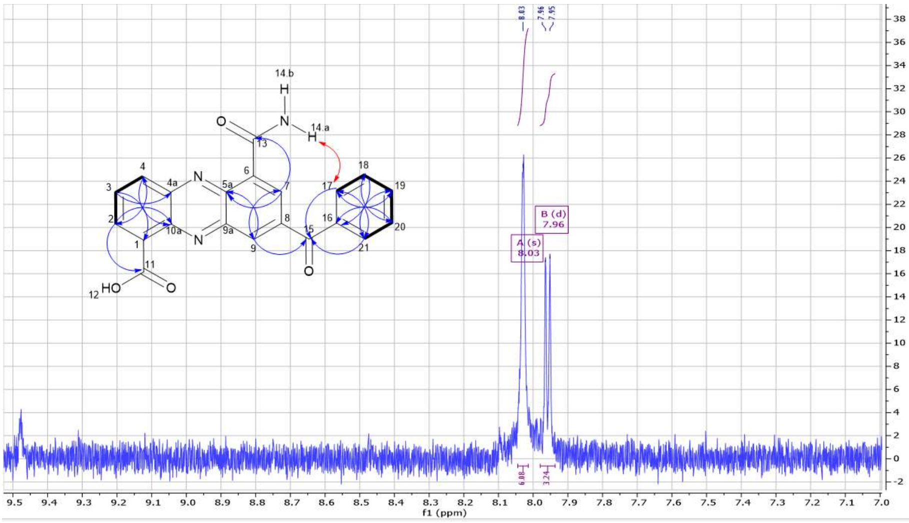

Figure S30. 1D-NOESY spectrum of dermacozine P (**3**) (600 MHz, DMSO-d_6) from irradiation of the signal at 8.03 ppm.

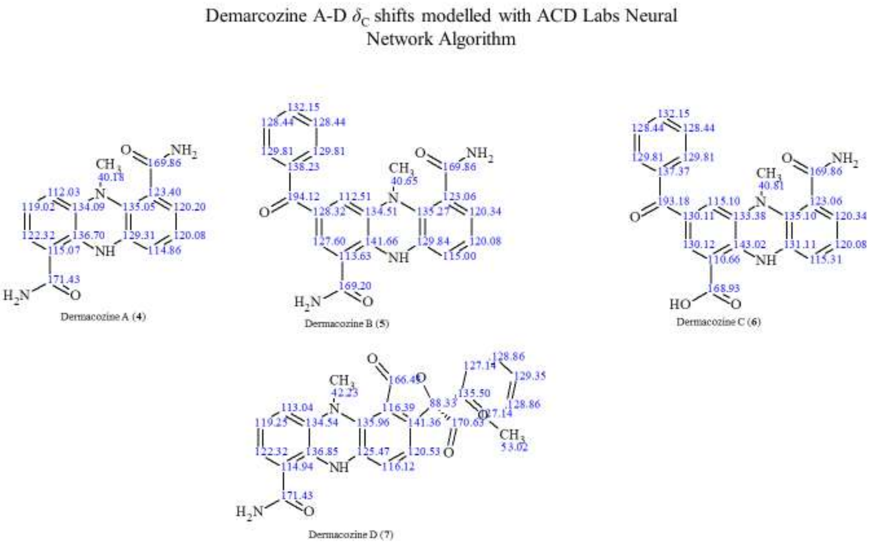

Figure S31. ^{13}C-NMR δ calculated values of dermacozine A-D (**4**-**7**) using the ACD Labs software with Neural Network Algorhitm Algorithm, solvent DMSO-d_6

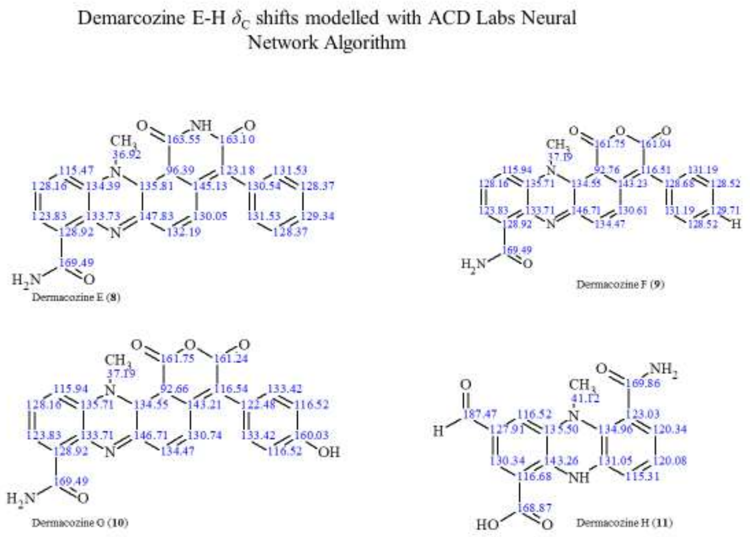

Figure S32. ^{13}C-NMR δ calculated values of dermacozine E-H (**8**-**11**) using the ACD Labs Software with Neural Network Algorhitm Algorithm, solvent DMSO-d_6

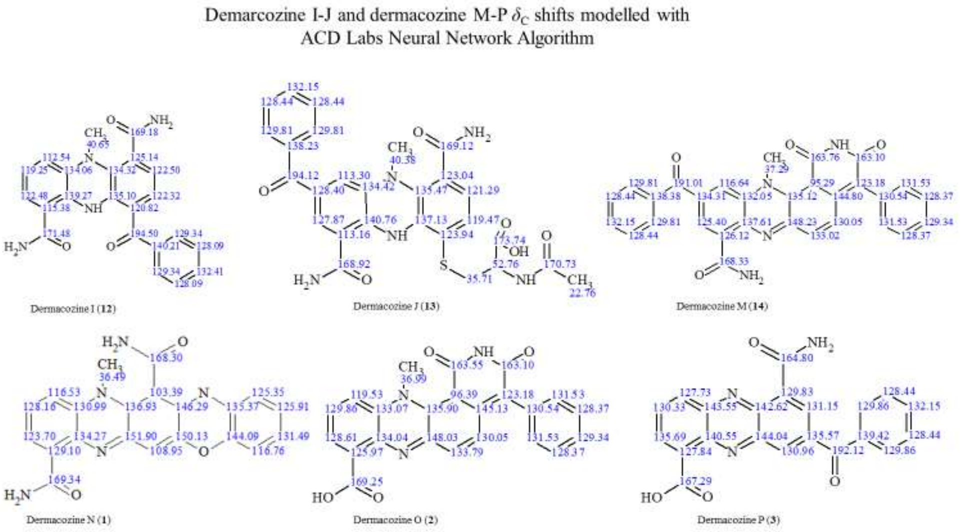

Figure S33. ^{13}C-NMR δ calculated values of dermacozine I-J (**12**-**13**) and dermacozine M-P (**14**, **1**-**3**) using the ACD Labs Software with Neural Network Algorhitm Algorithm, solvent DMSO-d_6

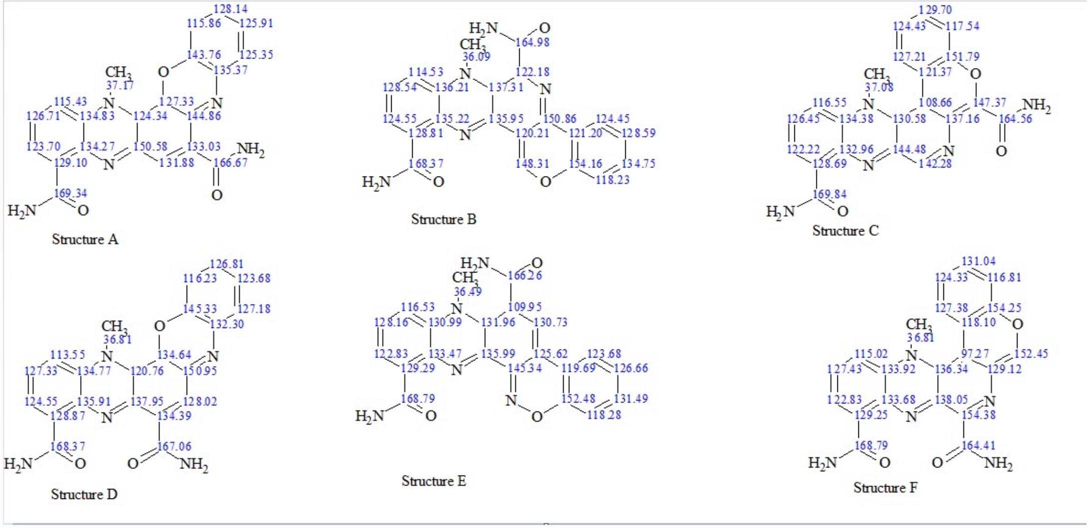

Figure S34. ^{13}C-NMR δ calculated values of possible structures (**A**-**F**) of dermacozine N (**1**), using the ACD Labs Software with Neural Network Algorhitm Algorithm, solvent DMSO-d_6

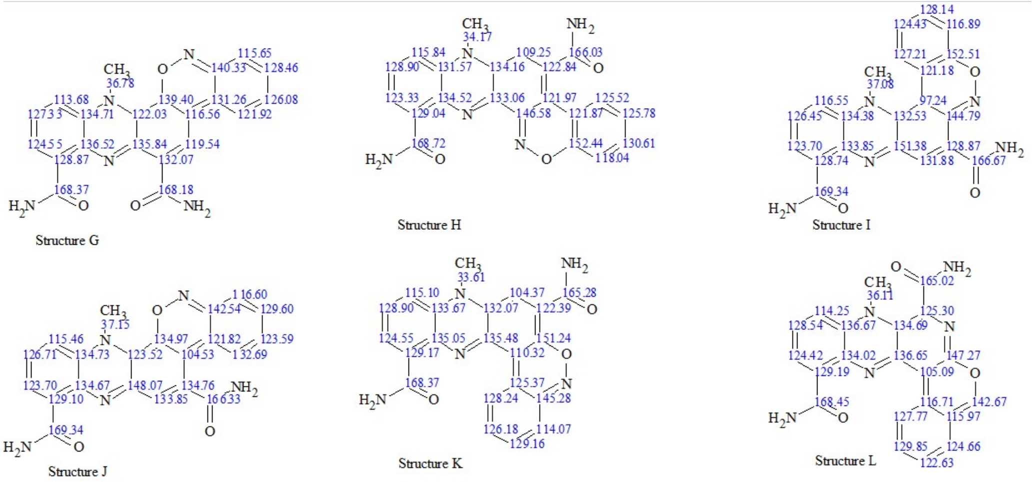

Figure S35. ^{13}C-NMR δ calculated values of possible structures (**G**-**L**) of dermacozine N (**1**), using the ACD Labs Software with Neural Network Algorithm, solvent DMSO-d_6

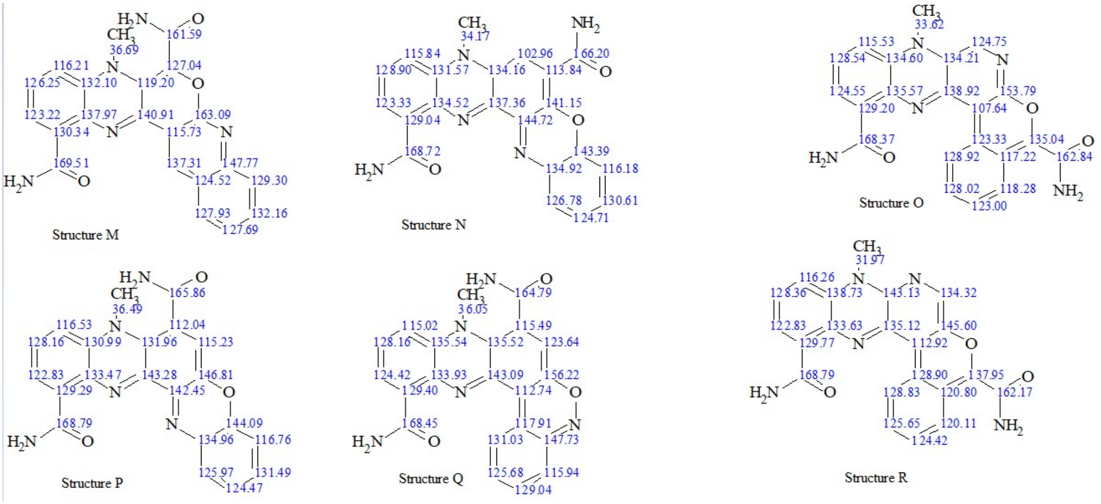

Figure S36. ^{13}C-NMR δ calculated values of possible structures (**M**-**R**) of dermacozine N (**1**), using the ACD Labs Software with Neural Network Algorithm, solvent DMSO-*d*6

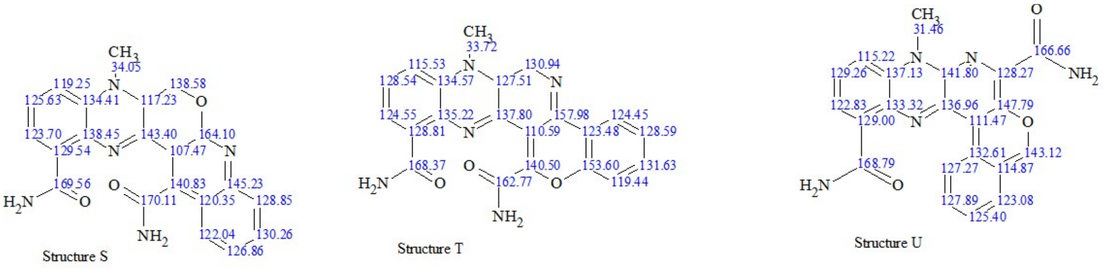

Figure S37. ^{13}C-NMR δ calculated values of possible structures (**S**-**U**) of dermacozine N (**1**), using the ACD Labs Software with Neural Network Algorithm, solvent DMSO-*d*6

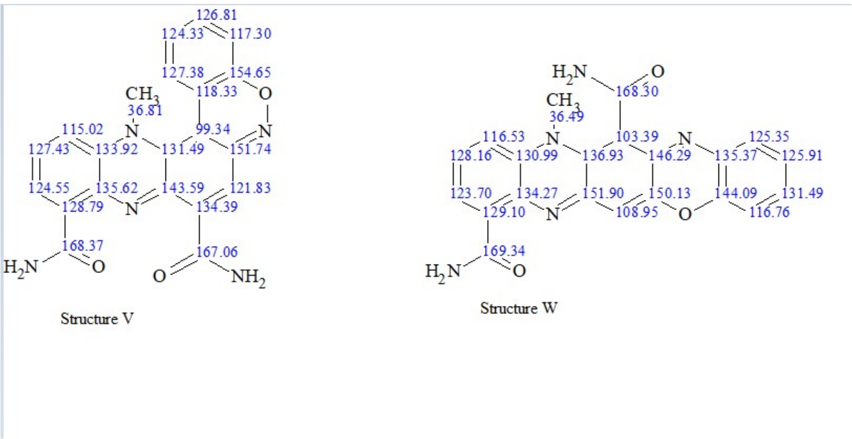

Figure S38. ^{13}C-NMR δ calculated values of possible structures (**V** and **W**) of dermacozine N (**1**), using the ACD Labs Software with Neural Network Algorithm, solvent DMSO-*d*6

Dermacozine Atom Numbers	Experimental $\delta^{13}C$ Dermacozine A	Calculated $\delta^{13}C$ Dermacozine A	Experimental $\delta^{13}C$ Dermacozine B	Calculated $\delta^{13}C$ Dermacozine B	Experimental $\delta^{13}C$ Dermacozine C	Calculated $\delta^{13}C$ Dermacozine C	Experimental $\delta^{13}C$ Dermacozine D	Calculated $\delta^{13}C$ Dermacozine D	Experimental $\delta^{13}C$ Dermacozine E	Calculated $\delta^{13}C$ Dermacozine E
1	112.3	115.1	111.2	113.6	107.4	110.7	110.9	114.9	131.7	128.9
2	121.2	122.3	126.6	127.6	129.2	130.1	120.3	122.3	127.1	123.8
3	119.8	119.0	128.4	128.3	127.9	130.1	120.2	119.3	130.7	128.2
4	116.0	112.0	114.7	112.5	114.3	115.1	117.4	113.0	119.6	115.5
4a	138.8	134.1	138.9	134.5	138.8	133.4	138.7	134.5	133.7	134.4
5a	133.8	135.1	133.5	135.3	133.3	135.1	137.4	136.0	139.0	135.8
6	124.5	123.4	124.7	123.1	124.6	123.1	109.9	116.4	99.8	96.4
7	122.5	120.2	123.9	120.3	124.3	120.3	140.3	141.4	139.6	145.1
8	121.6	120.1	121.9	120.1	121.6	120.1	117.3	120.5	133.8	130.1
9	113.7	114.9	114.8	115.0	115.3	115.3	117.2	116.1	130.2	132.2
9a	138.0	129.3	135.8	129.8	134.5	131.1	138.6	125.5	150.2	147.8
10a	140.6	136.7	144.6	141.7	145.7	143.0	140.9	136.9	135.2	133.7
11	171.1	171.4	170.7	169.2	169.1	168.9	171.0	171.4	166.2	169.5
N-methyl	40.1	40.2	40.0	40.7	40.0	40.8	43.5	42.2	45.9	36.9

Dermacozine Atom Numbers	Experimental $\delta^{13}C$ Dermacozine F	Calculated $\delta^{13}C$ Dermacozine F	Experimental $\delta^{13}C$ Dermacozine G	Calculated $\delta^{13}C$ Dermacozine G	Experimental $\delta^{13}C$ Dermacozine H	Calculated $\delta^{13}C$ Dermacozine H	Experimental $\delta^{13}C$ Dermacozine I	Calculated $\delta^{13}C$ Dermacozine I	Experimental $\delta^{13}C$ Dermacozine J	Calculated $\delta^{13}C$ Dermacozine J
1	131.9	128.9	131.8	128.9	117.6	116.7	114.8	115.4	111.0	113.2
2	127.2	123.8	127.3	123.8	132.8	130.3	121.3	122.5	125.8	127.9
3	130.9	128.2	130.9	128.2	127.3	127.9	121.1	119.3	128.2	128.4
4	119.5	115.9	119.7	115.9	108.5	116.5	115.8	112.5	114.3	113.3
4a	133.8	135.7	133.6	135.7	137.9	135.5	138.5	134.1	138.1	134.4
5a	141.9	134.6	141.7	134.6	133.0	135.0	134.8	134.3	133.4	135.5
6	95.2	92.8	95.4	92.7	123.8	123.0	125.4	125.1	123.5	123.0
7	142.7	143.2	142.5	143.2	122.3	120.3	120.1	122.5	122.6	121.3
8	134.0	130.6	133.7	130.7	120.9	120.1	124.2	122.3	126.6	119.5
9	130.4	134.5	129.9	134.5	113.2	115.3	116.9	120.8	117.8	123.9
9a	150.4	146.7	150.3	146.7	136.0	131.1	141.9	135.1	136.8	137.1
10a	135.4	133.7	135.4	133.7	145.7	143.3	136.5	139.3	143.2	140.8
11	166.4	169.5	166.3	169.5	168.7	168.9	169.7	171.5	169.9	168.9
N-methyl	46.0	37.2	46.3	37.2	39.3	41.1	40.0	40.7	39.7	40.4

Table S1. Comparison between calculated vs. experimental ^{13}C-NMR δ values of dermacozines A-J (**4-13**)

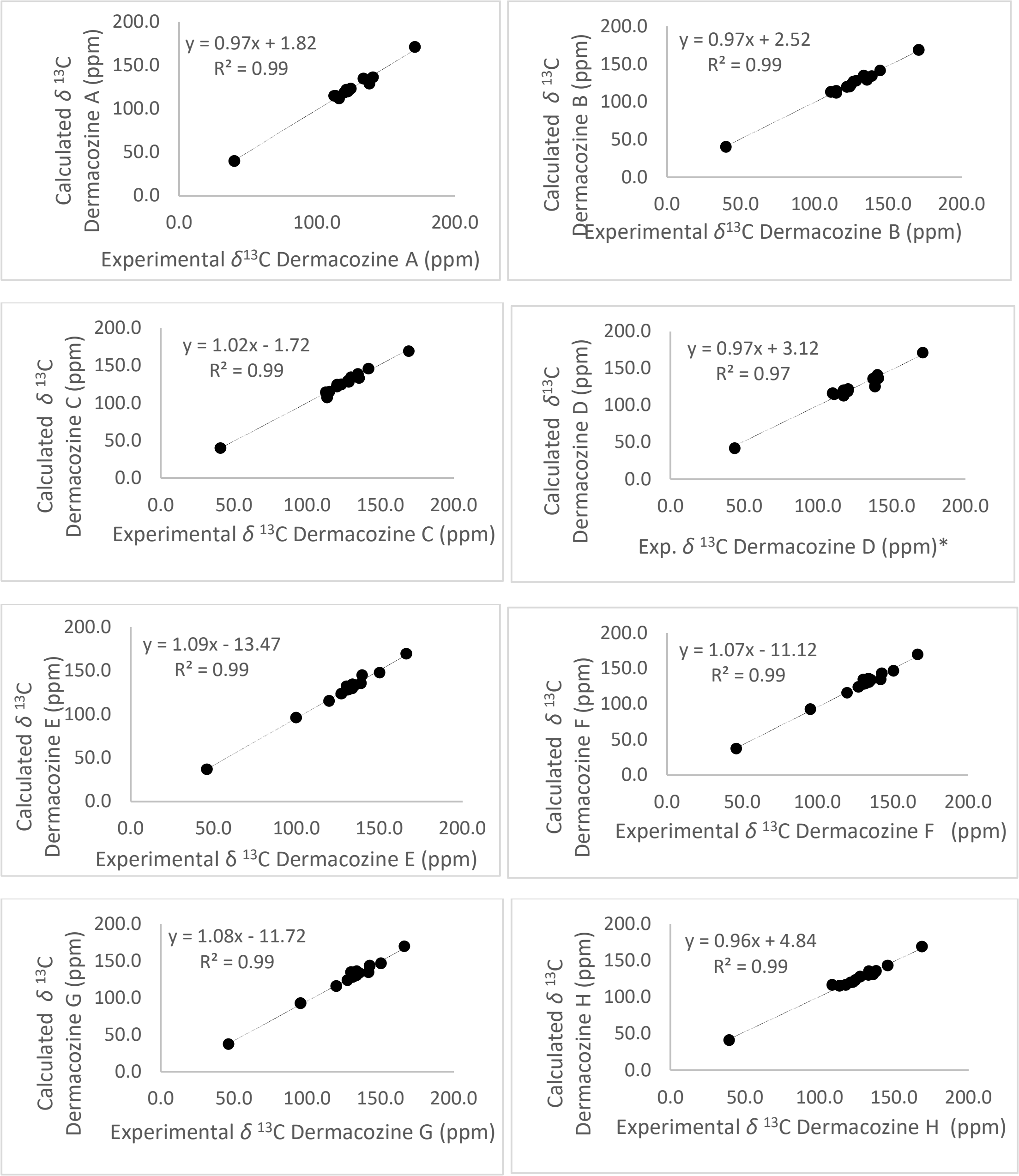

Figure 39. Linear regression graphics between ACD Labs (Neural Network Algorithm) calculated vs. experimental ^{13}C-NMR δ values of dermacozine A-H (**4-11**)

*Dermacozine D experimental values were recorded in Chloroform-*d*.

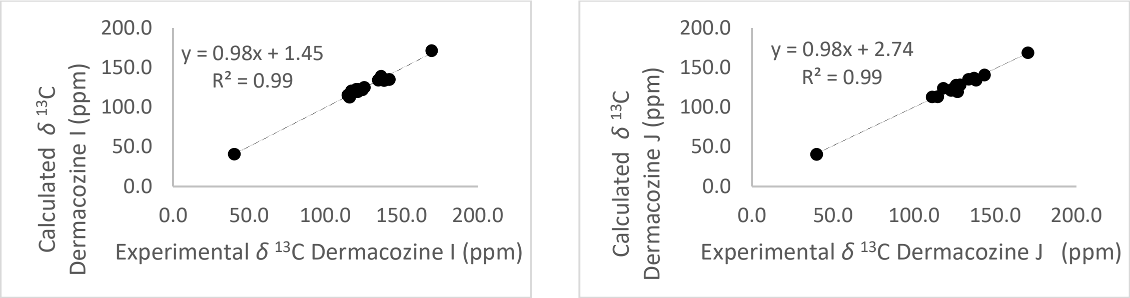

Figure 40. Linear regression graphics between ACD Labs (Neural Network Algorithm) calculated vs. experimental ^{13}C-NMR δ values of dermacozine I & J (**12-13**)

Dermacozine N Atom Numbers	Experimental δ ^{13}C Dermacozine N	Calculated δ ^{13}C Structure A	Calculated δ ^{13}C Structure D	Calculated δ ^{13}C Structure E	Calculated δ ^{13}C Structure G	Calculated δ ^{13}C Structure H	Calculated δ ^{13}C Structure I	Calculated δ ^{13}C Structure J	Calculated δ ^{13}C Structure K	Calculated δ ^{13}C Structure N	Calculated δ ^{13}C Structure P	Calculated δ ^{13}C Structure Q	Calculated δ ^{13}C Structure V	Calculated δ ^{13}C Structure W
1	128.9	129.1	128.9	129.3	128.9	129.0	128.7	129.1	129.2	129.0	129.3	129.4	128.8	129.1
2	125.9	123.7	124.6	122.8	124.6	123.3	123.7	123.7	124.6	123.3	122.8	124.4	124.6	123.7
3	128.4	126.7	127.3	128.2	127.3	128.9	126.5	126.7	128.9	128.9	128.2	128.2	127.4	128.2
4	118.0	115.4	113.6	116.5	113.7	115.8	116.6	115.5	115.1	115.8	116.5	115.0	115.0	116.5
4a	134.4	134.8	134.8	131.0	134.7	131.6	134.4	134.7	133.7	131.6	131.0	135.5	133.9	131.0
5a	135.5	124.3	120.8	132.0	122.0	134.2	132.5	123.5	132.1	134.2	132.0	135.5	131.5	136.9
6	109.8	127.3	134.6	110.0	139.4	109.3	97.2	135.0	104.4	103.0	112.0	115.5	99.3	103.4
7	148.1	144.9	151.0	130.7	116.6	122.8	144.8	104.5	122.4	113.8	115.2	123.6	151.7	146.3
8	149.8	133.0	128.0	125.6	119.5	122.0	128.9	134.8	151.2	141.2	146.8	156.2	121.8	150.1
9	105.9	131.9	134.4	145.3	132.1	146.6	131.9	133.9	110.3	144.7	142.5	112.7	134.4	109.0
9a	151.6	150.6	138.0	136.0	135.8	133.1	151.4	148.1	135.5	137.4	143.3	143.1	143.6	151.9
10a	135.1	134.3	135.9	133.5	136.5	134.5	133.9	134.7	135.1	134.5	133.5	133.9	135.6	134.3
11	166.3	169.3	168.4	168.8	168.4	168.7	169.3	169.3	168.4	168.7	168.8	168.5	168.4	169.3
15	143.3	143.8	145.3	152.5	140.3	152.4	152.5	142.5	145.3	143.4	144.1	147.7	154.7	144.1
16	115.0	115.9	116.2	118.3	115.7	118.0	116.9	116.6	114.1	116.2	116.8	115.9	117.3	116.8
17	127.9	128.1	126.8	131.5	128.5	130.6	128.1	129.6	129.2	130.6	131.5	129.0	126.8	131.5
18	125.3	125.9	123.7	126.7	126.1	125.8	124.4	123.6	126.2	124.7	124.5	125.7	124.3	125.9
19	127.2	125.4	127.2	123.7	121.9	125.5	127.2	132.7	128.2	126.8	126.0	131.0	127.4	125.4
20	134.8	135.4	132.3	119.7	131.3	121.9	121.2	121.8	125.4	134.9	135.0	117.9	118.3	135.4
21	39.5	37.2	36.8	36.5	36.8	34.2	37.1	37.2	33.6	34.2	36.5	36.1	36.8	36.5

Table S2. Comparison between the experimental ^{13}C-NMR δ values of dermacozine N (**1**) vs. the calculated ones of possible structures **A**, **D**, **E**, **G**, **H**, **I**, **J**, **K**, **N**, **P**, **Q**, **V**, **W** by ACD Labs (Neural Network Algorithm, DMSO-d_6)

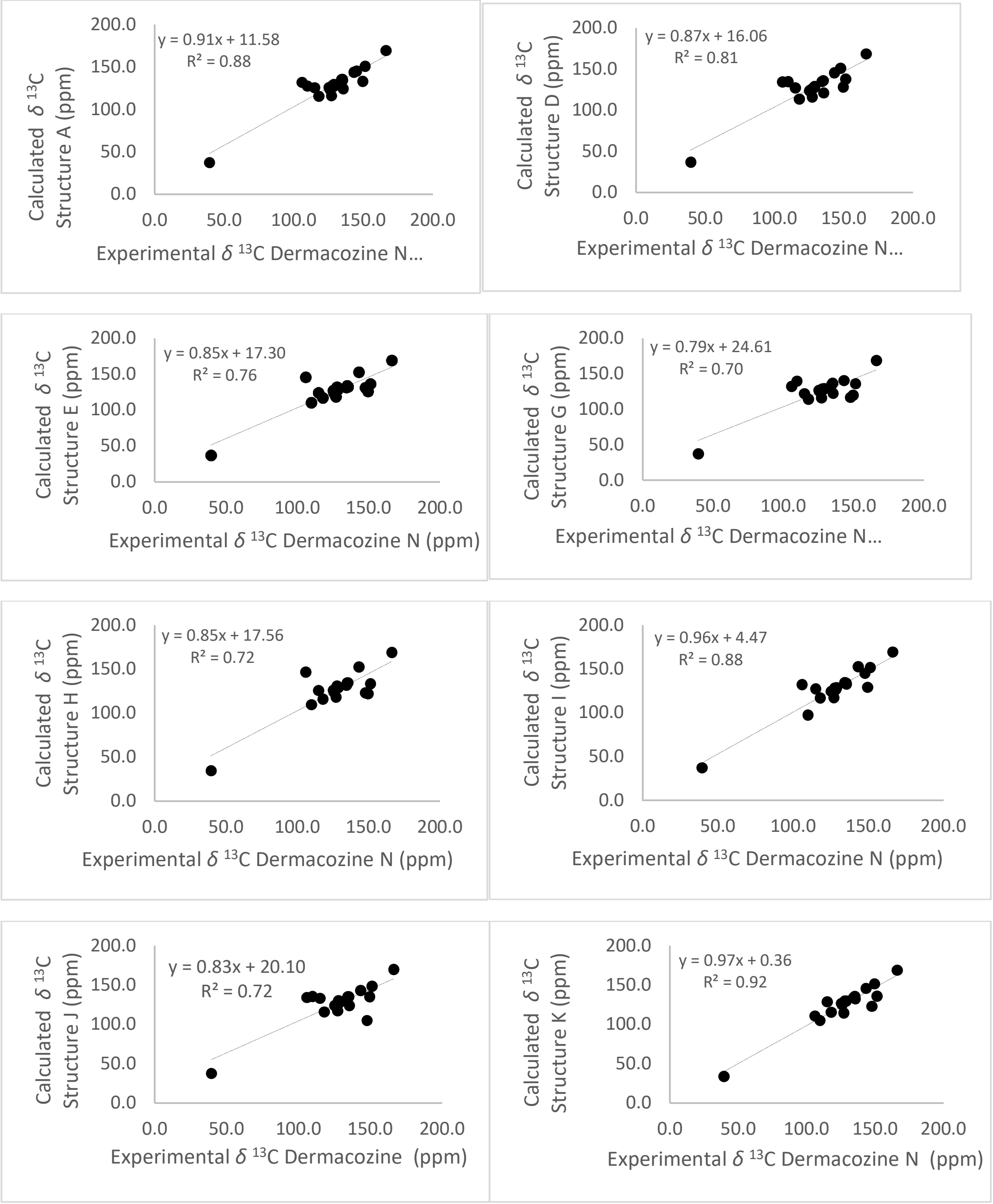

Figure S41. Linear regression graphics between ^{13}C-NMR δ experimental values of dermacozine N (**1**) vs. the ACD Labs calculated ones (Neural Network Algorithm, DMSO-d_6) (**4-13**)

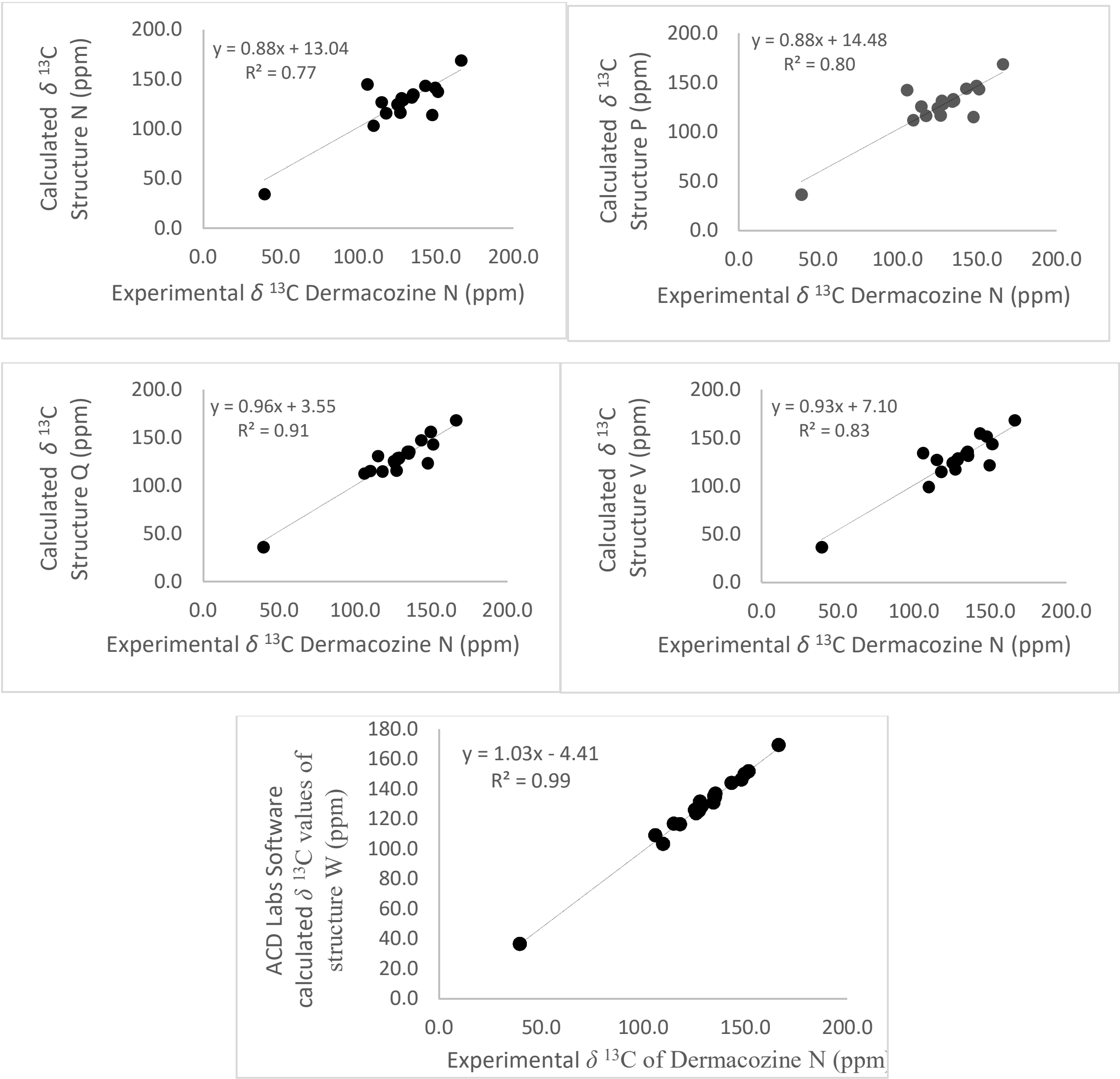

Figure S42. Linear regression graphics between ^{13}C-NMR δ experimental values of dermacozine N (**1**) vs. the ACD Labs (Neural Network Algorithm, DMSO-d_6) calculated ones for possible structures **N**, **P**, **Q**, **V**, **W**

Dermacozine N Atom Numbers	Experimental δ ^{13}C Dermacozine N	Calculated δ ^{13}C Structure W	Absolute error between the Experimental and Calculated data points (ppm)	Standard Deviation from the mean(*σ)
1	128.9	129.1	-0.2	-0.1
2	125.9	123.7	2.2	0.9
3	128.4	128.2	0.2	0.1
4	118	116.5	1.5	0.6
4a	134.4	131	3.4	1.4
5a	135.5	136.9	-1.4	-0.6
6	109.8	103.4	6.4	2.7
7	148.1	146.3	1.8	0.7
8	149.8	150.1	-0.3	-0.1
9	105.9	109	-3.1	-1.3
9a	151.6	151.9	-0.3	-0.1
10a	135.1	134.3	0.8	0.3
11	166.3	169.3	-3	-1.3
15	143.3	144.1	-0.8	-0.3
16	115	116.8	-1.8	-0.7
17	127.9	131.5	-3.6	-1.5
18	125.3	125.9	-0.6	-0.3
19	127.2	125.4	1.8	0.7
20	134.8	135.4	-0.6	-0.2
20	134.8	135.4	-0.6	-0.2
21	39.5	36.5	3	1.3

Table S3. Absolute Error and the standard deviation of the error from the mean between the calculated (Structure **W**) and the experimental dermacozine N (**1**) ^{13}C-NMR chemical shifts

Nr.	Dermacozine E (**8**) δ ^{13}C	Dermacozine F (**9**) δ ^{13}C	Dermacozine G (**10**) δ ^{13}C	Dermacozine O (**2**) δ ^{13}C
1	131.7	131.9	131.8	129.6
2	127.1	127.2	127.3	126.6
3	130.7	130.9	130.9	131.3
4	119.6	119.5	119.7	120.3
4a	133.7	133.8	133.6	134.0
5a	139.0	141.9	141.7	139.5
6	99.8	95.2	95.4	100.4
7	139.6	142.7	142.5	139.6
8	133.8	134.0	133.7	134.5
9	130.2	130.4	129.9	129.7
9a	150.2	150.4	150.3	150.6
10a	135.2	135.4	135.4	135.3
11	166.2	166.4	166.3	167.2
16	121.7	119.7	119.8	122.6
17	134.2	134.5	124.3	134.1
18	131.2	131.4	132.3	131.3
19	128.1	128.3	115.2	128.2
20	127.7	127.9	158.3	127.9
21	128.1	128.3	115.2	128.2
22	131.2	131.4	132.3	131.3
23	45.9	46.0	46.3	45.9

Table S4. Experimental values of ^{13}C-NMR δ chemical shifts of dermacozines E (**8**), F (**9**), G (**9**) and O (**2**) for multiple regression and t-test

SUMMARY OUTPUT								
Regression Statistics								
Multiple R	0.999602							
R Square	0.999205							
Adjusted R Square	0.999064							
Standard Error	0.687836							
Observations	21							
ANOVA								
	df	*SS*	*MS*	*F*	*ignificance F*			
Regression	3	10105.71	3368.569	7119.931	1.55E-26			
Residual	17	8.043009	0.473118					
Total	20	10113.75						
	Coefficients	*andard Err*	*t Stat*	*P-value*	*Lower 95%*	*Upper 95%*	*ower 95.0%*	*pper 95.0%*
Intercept	-0.44071	0.939796	-0.46895	0.645066	-2.42351	1.542083	-2.42351	1.542083
Dermacozine E (8) δ 13C	1.107188	0.113396	9.763937	2.19E-08	0.867944	1.346432	0.867944	1.346432
Dermacozine F (9) δ 13C	-0.1067	0.11218	-0.95118	0.354841	-0.34338	0.129976	-0.34338	0.129976
Dermacozine G (10) δ 13C	0.00425	0.018576	0.22881	0.821746	-0.03494	0.043442	-0.03494	0.043442

Hypotheses:

H$_0$:(null hypothesis) = the structure of dermacozine E (**8**) and/or dermacozine F (**9**) and/or dermacozine G (**10**) are not showing significant similarity to dermacozine O (**2**) (δ_C values of dermacozine E and/or dermacozine F and/or dermacozine G are independent on the corresponding δ_C values of dermacozine O).

H$_a$:(alternative hypothesis) = the structure of dermacozine E (**8**) and/or dermacozine F (**9**) and/or dermacozine G (**10**) are showing significant similarity to dermacozine O (**2**) (δ_C values of dermacozine E and/or dermacozine F and/or dermacozine G are dependent on the corresponding δ_C values Dermacozine O).

Hypothesis testing:

df (degrees of freedom) = n(number of δ_C compared)-p(number of dependent variables)-1 = 17

α = .05

CI = 95%

$t_{\alpha/2}$ rejection region **(RR)** (two tailed t-test table value) = $t_{\alpha/2} \leq -2.11$ and $+2.11 \leq t_{\alpha/2}$

$t_{\alpha/2}$ values calculated, multiple regression algorithm:

$t_{\alpha/2}$ calculated value for the δ_C values in ppm of dermacozine E (**8**) = + 9.76;

$t_{\alpha/2}$ calculated value for the δ_C values in ppm of dermacozine F (**9**) = − 0.95;

$t_{\alpha/2}$ calculated value for the δ_C values in ppm of dermacozine G (**10**) = + 0.23;

As for dermacozine F (**9**) and dermacozine G (**10**), (calculated) $t_{\alpha/2}$ is between − 2.11 and +2.11(two tailed t-test table value for α = .05; *df* = 17), which is essentially means that − $t_{\alpha/2}$ (t-test table value) < $t_{\alpha/2}$ dermacozine F (**9**) (calculated) < + $t_{\alpha/2}$ (t-test table value) and − $t_{\alpha/2}$(t-test table value) < $t_{\alpha/2}$ dermacozine G (**10**) (calculated) < + $t_{\alpha/2}$ (t-test table value). (Calculated $t_{\alpha/2}$ values for both molecules are not within the **RR** region of the t-test distribution). Therefore, we cannot reject the null hypothesis for dermacozine F (**9**) and dermacozine G (**10**). As a consequence, the δ_C values of these compounds are independent on the δ_C values of the structure of dermacozine O (**2**). However, when we were investigating $t_{\alpha/2}$ of dermacozine E (**8**) (calculated) – where there is only one substituent difference, an $-NH_2$ difference at position C-11 of which can change carbon shifts compared to corresponding carbon shifts in dermacozine O (**2**), we can see that + 9.76 $t_{\alpha/2}$ (calculated $t_{\alpha/2}$ dermacozine E) >> + 2.11 $t_{\alpha/2}$ t(two tailed t-test table value. Therefore, $t_{\alpha/2}$ (calculated $t_{\alpha/2}$ dermacozine E (**8**)) falls into the rejection

region **(RR)** of the t-distribution, and as a consequence, we can reject the null hypothesis for dermacozine E (**8**) and we can accept the alternative hypothesis.

Conclusion of hypothesis testing:

The δ_C values of dermacozine O (**2**) significantly dependent on dermacozine E (**8**). Therefore, we can conclude at 95% Confidence Level that the structure of dermacozine E (**8**) is the most similar structure to dermeacozine O (**2**) among the structures of dermacozine E (**8**), F (**9**) and G (**10**), based on their corresponding δ_C values investigated with multiple regression and a two-tailed t-test.

Figure S43. Multiple regression analysis between the experimental ^{13}C-NMR δ values of dermacozines E (**8**), F (**9**), G (**9**) as independent variables and those of dermacozine O (**2**) as dependent variables

RESIDUAL OUTPUT		
Observation	*Predicted Dermacozine O (2) δ 13C*	*Residuals*
1	131.9	-2.3
2	127.3	-0.7
3	130.9	0.4
4	119.7	0.6
5	133.9	0.1
6	138.9	0.6
7	100.3	0.1
8	139.5	0.1
9	134.0	0.5
10	130.4	-0.7
11	150.4	0.2
12	135.4	-0.1
13	166.5	0.7
14	122.0	0.6
15	134.3	-0.2
16	131.4	-0.1
17	128.2	0.0
18	128.0	-0.1
19	128.2	0.0
20	131.4	-0.1
21	45.7	0.2

Table S5. Residuals between the observed and predicted values of ^{13}C-NMR δ of dermacozines E (**8**), F (**9**), G (**9**) as independent variables and those of dermacozine O (**2**) as dependent variables

Atom Number	Experimental δ ^{13}C values of Dermacozine P	ACD Labs Calculated (Neural Network Algorithm) δ ^{13}C values of Dermacozine P
1	131.5	127.8
2	134.9	135.7
3	132.4	130.3
4	132.6	127.7
4a	142.4	143.6
5a	140.7	142.6
7	135.1	131.2
9	131.3	131.0
10a	142.4	140.5
11	165.5	167.3
13	166.6	164.8
15	194.4	192.1
16	136.3	139.4
17	130.0	129.9
18	128.7	128.4
19	133.5	132.2
20	128.7	128.4

Table S6. Comparison between experimental and the ACD Labs calculated ^{13}C-NMR δ chemical shift values of dermacozines P (**3**) (Neural Network Algorithm, DMSO-d_6)

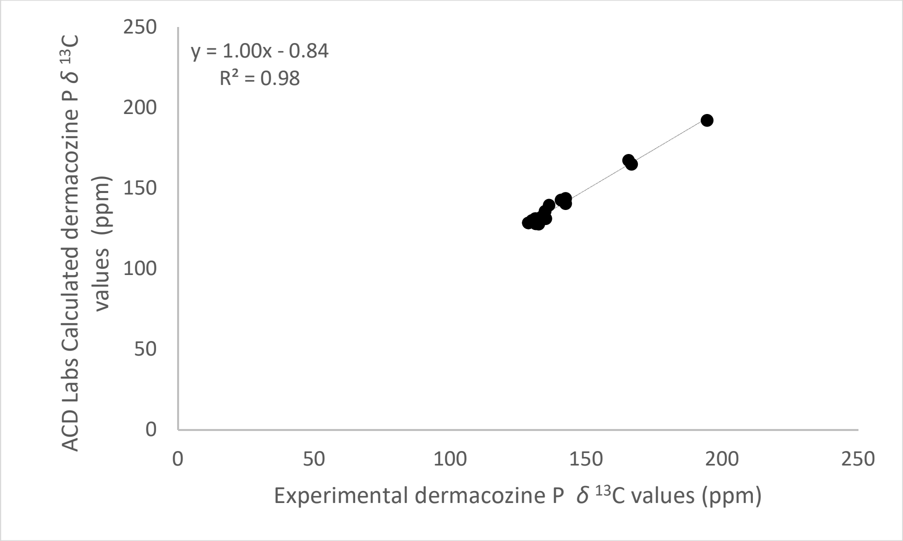

Figure S44. Linear regression graphics between the experimental ^{13}C-NMR δ chemical shifts of dermacozine P (**3**) and the ACD Labs calculated ^{13}C-NMR δ values (Neural Network Algorithm, DMSO-d_6)

Atom Number	Experimental δ ^{13}C values of Dermacozine P	ACD Labs Calculated (Neural Network Algorithm) δ ^{13}C values of Dermacozine P	Absolute Error (ppm)	Standard Deviation from the mean(*σ)
1	131.5	127.8	3.7	1.7
2	134.9	135.7	-0.8	-0.4
3	132.4	130.3	2.1	1.0
4	132.6	127.7	4.9	2.2
4a	142.4	143.6	-1.2	-0.5
5a	140.7	142.6	-1.9	-0.9
7	131.3	131.2	0.1	0.0
9	135.1	131	4.1	1.9
10a	142.4	140.5	1.9	0.9
11	165.5	167.3	-1.8	-0.8
13	166.6	164.8	1.8	0.8
15	194.4	192.1	2.3	1.0
16	136.3	139.4	-3.1	-1.4
17	130	129.9	0.1	0.0
18	128.7	128.4	0.3	0.1
19	133.5	132.2	1.3	0.6
20	128.7	128.4	0.3	0.1

Table S7. Absolute Error and the standard deviation of the error from the mean between the calculated and the experimental dermacozine P (**3**) ^{13}C-NMR chemical shifts

A) B)

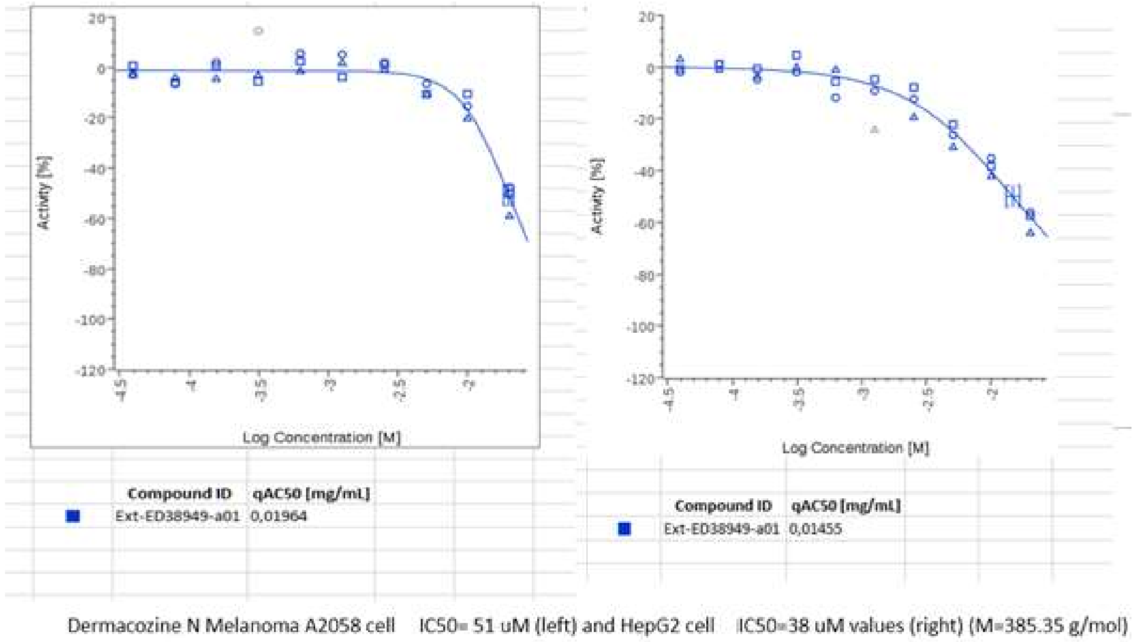

Figure S45. Evaluation of the cytotoxic activity of dermacozine N (**1**) against human A) Melanoma (A2058) and B) Hepatocellular carcinoma (HepG2) cell lines (IC_{50} graphs)

Bertalan Juhasz et al.

No.	Dermacozine N (1)				
	δC, mult	δH, mult (J in Hz)	HMBC	COSY	NOESY
1	128.9, C		3		
2	125.9, CH	7.88 (dd, 7.6, 1.3)	4	3	
3	128.4, CH	7.47 (td, 8.3, 7.6)		4, 2	
4	118.0, CH	7.55 (dd, 8.3, 1.3)	2	3	
4a	134.4, C		3, 23		
5a	135.5, C		9, 23		
6	109.8, C		14		
7	148.1, C		9		
8	149.8, C		9		
9	105.9, CH	6.79, s			12.A, 12.B
9a	151.6, C		9		
10a	135.1, C		2, 4		
11	166.3, C		2		
12		A 7.70, brs		12.B	12.B
		B 9.31, brs		12.A	12.A
13	168.3, C†				
14		A 7.65, brs		14.B	14.B
		B 7.98, brs		14.A	14.A
15	143.3, C		16, 17, 19		
16	115.0, CH	7.12 (dd, 7.6, 1.5)	18	17	
17	127.9, CH	7.19 (ddd, 7.6, 7.4, 1.6)	19	16, 18	
18	125.3, CH	7.15 (ddd, 7.6, 7.4, 1.5)	16	17, 19	
19	127.2, CH	7.30 (dd, 7.6, 1.6)	17	19	
20	134.8, C		16, 18		
21					
22					
23	39.5, CH_3	3.68, s			4

Table S8. Experimental NMR spectroscopic data for dermacozine N (**1**) with HMBC, COSY and NOESY correlations (800 MHz, DMSO-d_6)

†: Calculated with ACD Labs Software, Neural Network Algorithm, DMSO-d_6 (Chemical shift is not observed)

No.	*Dermacozine O* (**2**)			
	δ_C, mult	δ_H, mult (*J* in Hz)	HMBC	COSY
1	129.6, C		3	
2	126.6, CH	7.87 (dd, 7.5, 1.1)	4	3
3	131.3, CH	7.78 (td, 8.5, 7.5)		2, 4
4	120.3, CH	7.97 (dd, 8.5, 1.1)	2	3
4a	134.0, C		3, 23	
5a	139.5, C		9, 23	
6	100.4, C		8, 14	
7	139.6, C		8, 9	
8	134.5, CH	7.21 (d, 9.7)		9
9	129.7, CH	7.24 (d, 9.7)		8
9a	150.6, C		8,9	
10a	135.3, C		2,4	
11	167.2, C		2	
12		COOH, not observed		
13	163.6, C†			
14		11.27, bs		
15	163.1, C†			
16	122.6, C		8, 14, 18, 22	
17	134.1, C		19, 21	
18	131.3, CH	7.30 (dd, 7.4, 1.3)	19, 20	19/21
19	128.2, CH	7.47 (td, 7.4, 1.3)	18, 21	18/22, 20
20	127.9, CH	7.41 (td, 7.4, 1.3)	18, 21	19, 21
21	128.2, CH	7.47 (td, 7.4, 1.3)	19, 22	18/22, 20
22	131.3, CH	7.30 (dd, 7.4, 1.3)	20, 21	21/19
23	45.9, CH_3	3.67, s		

Table S9. Experimental NMR spectroscopic data for dermacozine O (**2**) with HMBC and COSY correlations (800 MHz, DMSO-d_6)

†: Calculated with ACD Labs Software, Neural Network Algorithm, DMSO-d_6 (Chemical shift is not observed)

No.	Dermacozine P (3)				
	δ_C, mult	δ_H, mult (J in Hz)	HMBC	COSY	Selective-NOESY, irrad. at 8.03 ppm
1	131.5, C		3		
2	134.9, CH	8.74 (dd, 7.0, 1.3)	4	3	
3	132.4, CH	8.21 (td, 7.0, 8.6)		4, 2	
4	132.6, CH	8.55 (dd, 8.6, 1.3)	2	3	
4a	142.4, C		3		
5a	140.7, C		7, 9		
6	129.8, C†				
7	131.3, CH	8.65 (d, 1.9)	9		
8	135.6, C†				
9	135.1, CH	8.95 (d, 1.9)	7		
9a	144.0, C†				
10a	141.2, C		2, 4		
11	165.5, C				
12		COOH,<br>not observed			
13	166.6, C		7		
14		A 8.03, brs<br>B 9.47, brs			17/21
15	194.4, C		9, 17, 21		
16	136.3, C		17, 18, 20, 21		
17	130.0, CH	7.95 (dd, 7.6, 1.3)	18, 19	18/20	
18	128.7, CH	7.66 (td, 7.6, 1.3)	20	19, 17/21	
19	133.5, CH	7.78 (td, 7.6, 1.3)	17, 21	18/20	
20	128.7, CH	7.66 (td, 7.6, 1.3)	18	19, 17/21	
21	130.0, CH	7.95 (dd, 7.6, 1.3)	17, 19	18/20	
22					
23	-	-	-	-	

Table S10. Experimental NMR spectroscopic data for dermacozine P (**3**) with HMBC and COSY correlations (600 MHz, DMSO-d_6)

†: Calculated with ACD Labs Software, Neural Network Algorithm, DMSO-d_6 (Chemical shift is not observed)

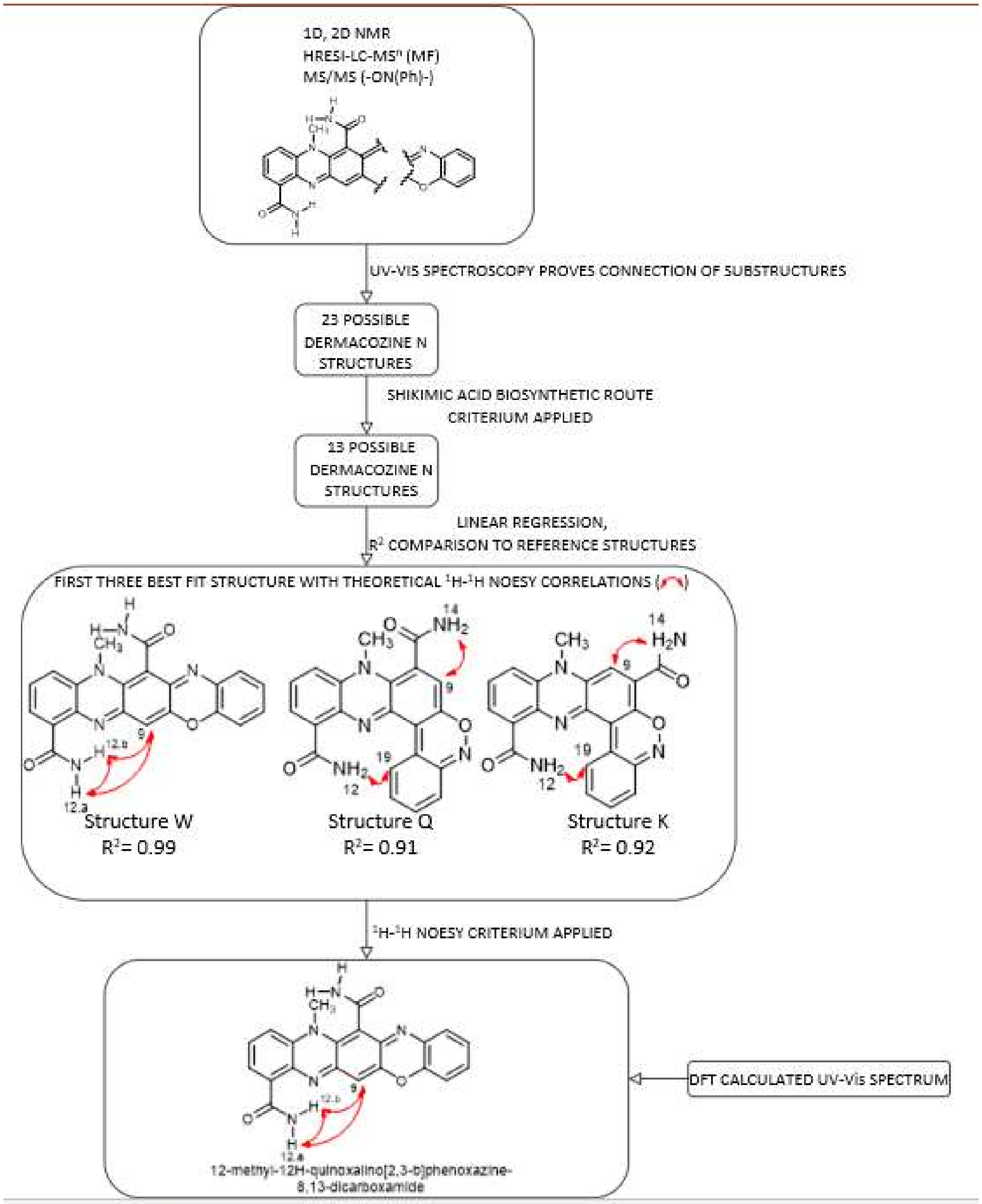

Figure S46. Workflow of dermacozine N (**1**) structure determination

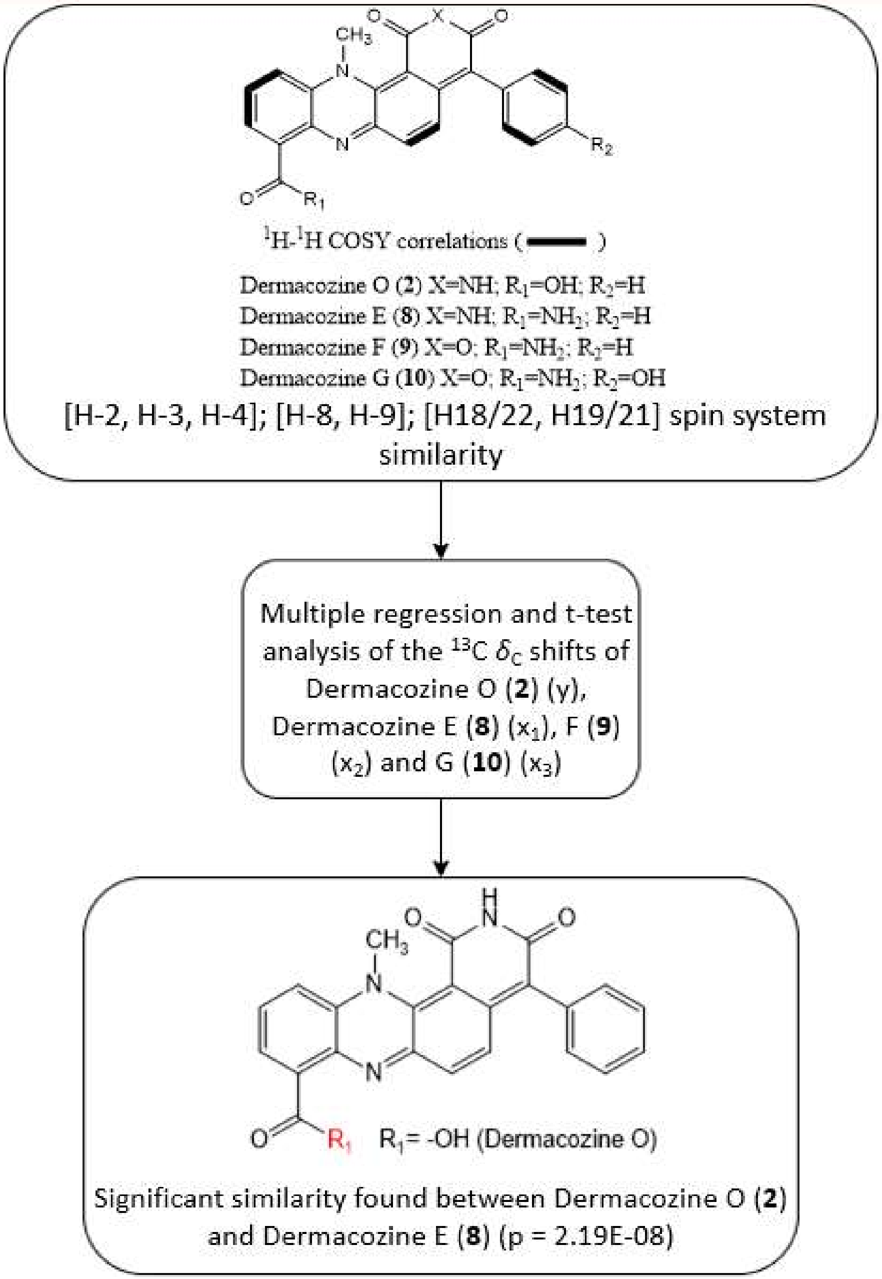

Figure S47. Workflow of dermacozine O (**2**) structure determination.

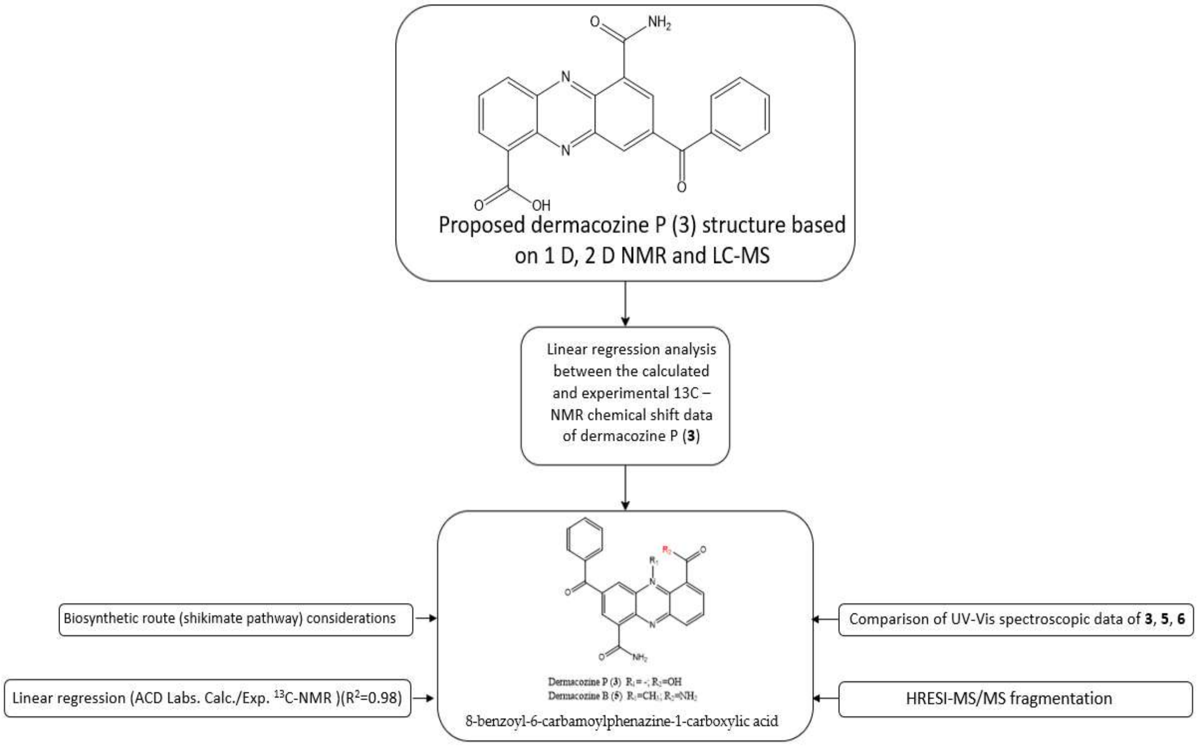

Figure S48. Workflow of dermacozine P (**3**) structure determination

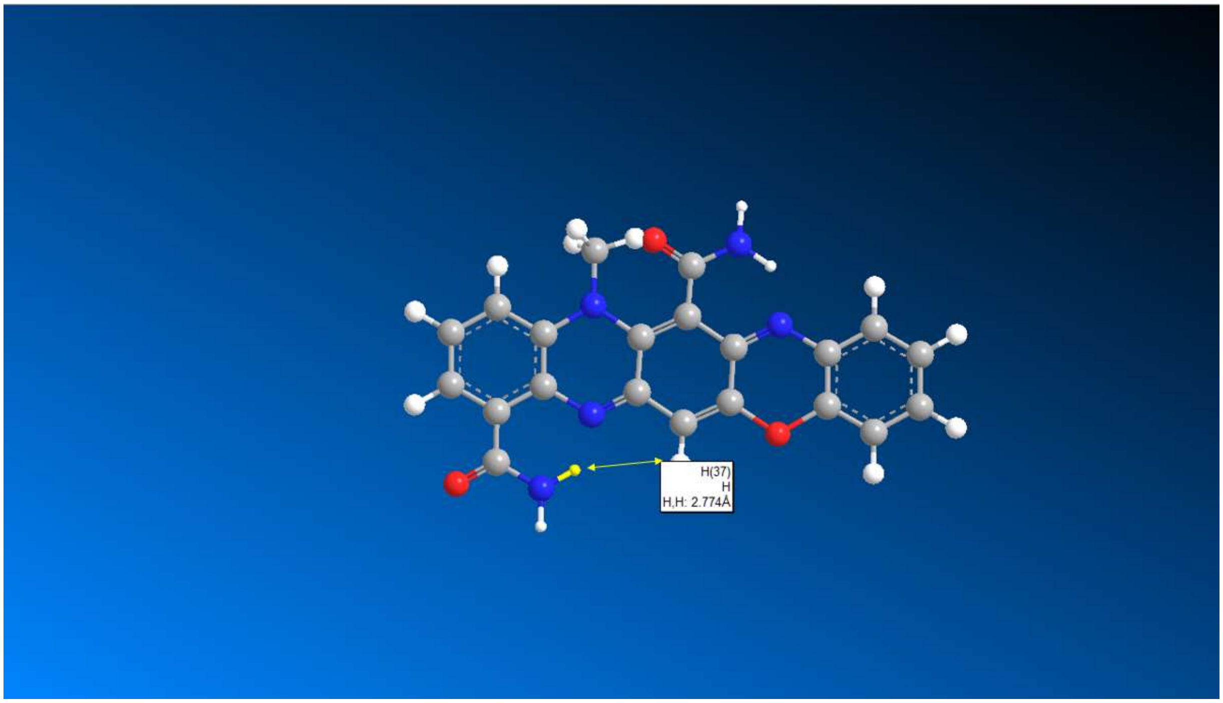

Theme S1. Dermacozine N (**1**) H-9 and NH_2-12 distance (Modelled with Chemdraw, Chem 3D)

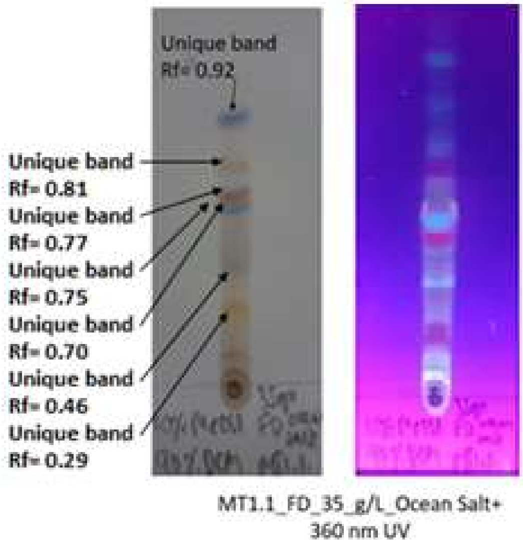

Theme S2. TLC plate of the initial FD fraction following Kupchan liquid-liquid partitioning, showing the colorful bands where the dermacozines were isolated from (left) and the same TLC plate under UV 360 nm light (right)

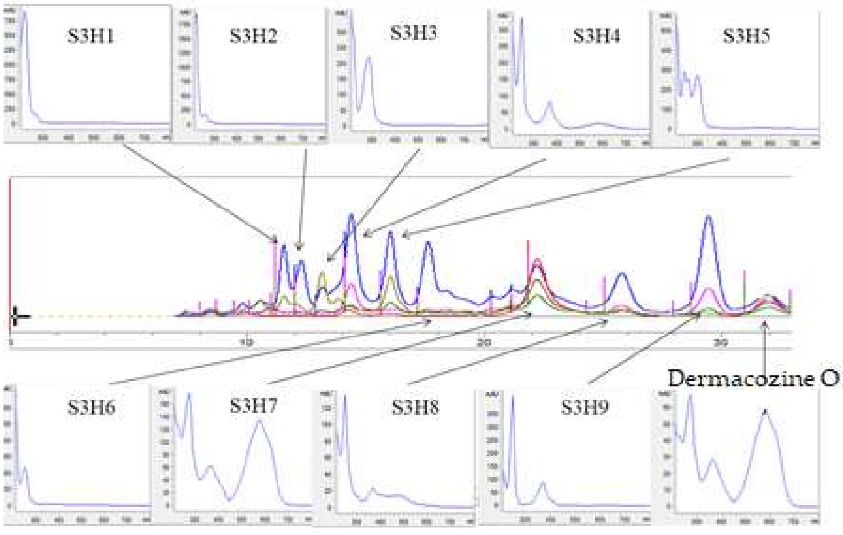

Theme S3. HPLC chromatogram for the isolation of dermacozine O (**2**)

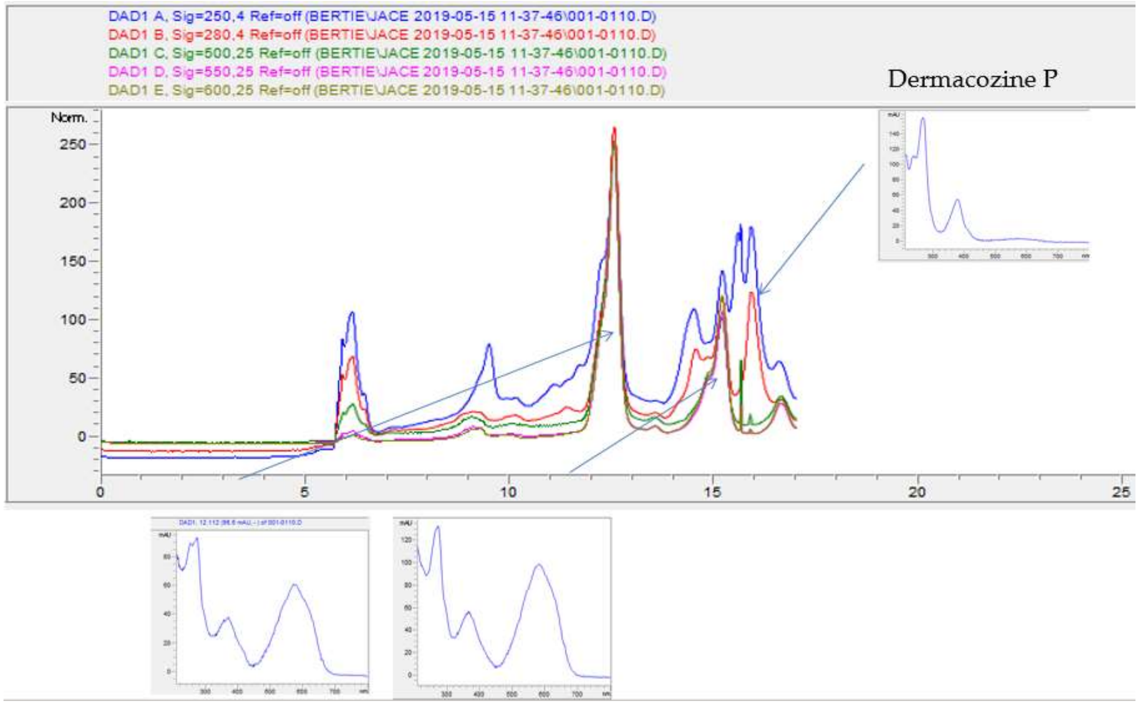

Theme S4. HPLC chromatogram for the isolation of dermacozine P (**3**)
